# Zinc catalysed electrophilic C–H borylation of heteroarenes†

**DOI:** 10.1039/d1sc01883c

**Published:** 2021-05-13

**Authors:** Matthew E. Grundy, Kang Yuan, Gary S. Nichol, Michael J. Ingleson

**Affiliations:** EaStCHEM School of Chemistry, University of Edinburgh Edinburgh EH9 3FJ UK michael.ingleson@ed.ac.uk

## Abstract

Cationic zinc Lewis acids catalyse the C–H borylation of heteroarenes using pinacol borane (HBPin) or catechol borane (HBCat). An electrophile derived from [IDippZnEt][B(C_6_F_5_)_4_] (IDipp = 1,3-bis(2,6-diisopropylphenyl)imidazol-2-ylidene) combined with *N*,*N*-dimethyl-*p*-toluidine (DMT) proved the most active in terms of C–H borylation scope and yield. Using this combination weakly activated heteroarenes, such as thiophene, were amenable to catalytic C–H borylation using HBCat. Competition reactions show these IDipp–zinc cations are highly oxophilic but less hydridophilic (relative to B(C_6_F_5_)_3_), and that borylation proceeds *via* activation of the hydroborane (and not the heteroarene) by a zinc electrophile. Based on DFT calculations this activation is proposed to proceed by coordination of a hydroborane oxygen to the zinc centre to generate a boron electrophile that effects C–H borylation. Thus, Lewis acid binding to oxygen sites of hydroboranes represents an under-developed route to access reactive borenium-type electrophiles for C–H borylation.

## Abstract 2

Cationic zinc Lewis acids catalyse the C–H borylation of heteroarenes using pinacol borane (HBPin) or catechol borane (HBCat).

## Graphical abstract

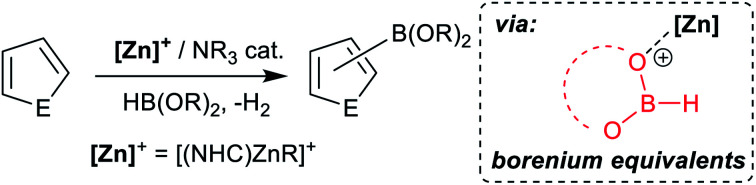

## Introduction

As it enables rapid construction of complex molecules, C–H functionalisation has become increasingly important.^1,2^ Among the most useful moieties made *via* C–H functionalisation are C–B containing units, owing in part to the power of the Suzuki–Miyaura reaction.^3,4^ This has made organoboranes ubiquitous nucleophiles and provides continued incentive to develop new routes to organoboranes,^5^ particularly *via* C–H borylation.^6^ Presently, the most common transformations of this type are based on noble metal catalysts, most often iridium.^7–11^ Base metal catalytic alternatives are preferable,^12,13^ with arguably the most powerful processes developed to date using cobalt catalysts.^14,15^ However, both cobalt and iridium have extremely low permitted daily exposure (PDE) values of 50 μg and 100 μg (oral intake).^16^ Alternative C–H borylation catalysts based on earth abundant elements that have higher PDE values are desirable. The last decade has seen some notable progress in this area, including in Fe catalysed arene C–H borylation,^17–21^ and in catalytic C–H borylation using boron based frustrated Lewis pairs (FLPs) and/or borenium cations.^22–26^ To date, these catalytic processes are either limited in scope or require the arene substrate to be present in super stoichiometric amounts. Addressing these limitations remains a significant challenge, the solution to which would be facilitated by identifying novel mechanisms to catalyse (hetero)arene C–H borylation.

**Fig. 1:**
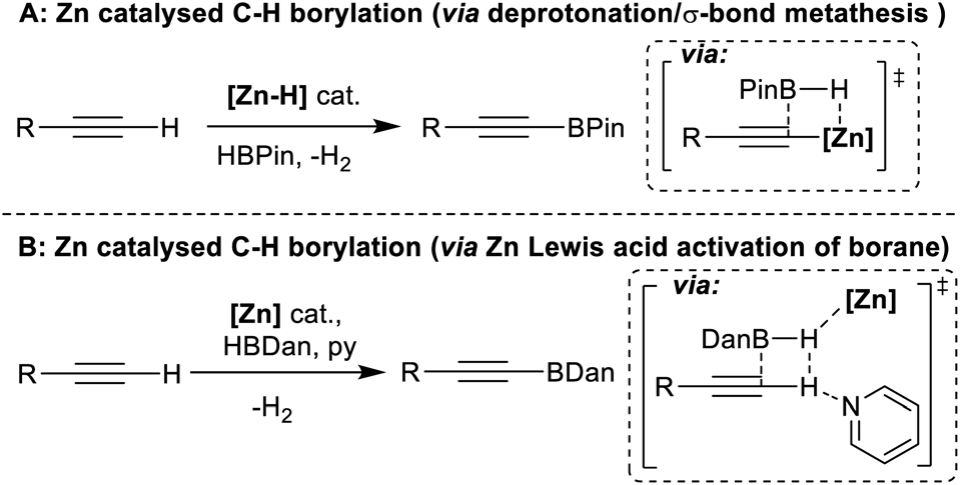
Proposed mechanisms for zinc catalysed alkyne C–H borylation.

One earth abundant high PDE element that has had limited use in catalysing arene borylation is zinc.^27–30^ While zinc catalysed C–H borylation of alkynes has been reported,^30,31^ no zinc complex that catalyses the C–H borylation of (hetero)arenes using hydroboranes has been reported to our knowledge. As zinc complexes are distinct to Fe/Co (zinc is redox neutral during catalysis) and to boron Lewis acids, different borylation mechanisms maybe accessible. Comparing zinc and boron Lewis acids, zinc Lewis acids are “harder”, *i.e.* more oxophilic and less hydridophilic, and contain more polarised ^*δ*+^Zn–H^*δ*−^ and ^*δ*+^Zn–C^*δ*−^ bonds (than B–H/B–C). The latter facilitated NHC–zinc catalysed alkyne C–H borylation with pinacolborane (HBPin), with B–C bond formation proposed to occur *via* alkyne deprotonation by a zinc hydride and then σ-bond metathesis of the Zn–C species with HBPin (Fig. 1A).^31^ However, another C–H borylation mechanism may be more relevant for substrates less acidic than alkynes, with no (hetero)arene deprotonation by a zinc-hydride reported to date to the best of our knowledge.^32^ One alternative mechanism involves activation of a hydroborane by a zinc Lewis acid. This would enhance electrophilicity at boron and form a functional equivalent of a borenium cation, species well documented to borylate π systems.^33^ Such a mechanism was outlined in zinc catalysed alkyne borylation with HBDan (HBDan = 1,8-naphthalenediaminatoborane), with zinc proposed to interact with the B–H unit (Fig. 1B), with pyridine then enabling C–H deprotonation.^30^ This can be viewed as a frustrated Lewis pair (FLP) borylation mechanism.^23^ It should be noted that related intermediates (*e.g.* [Zn]⋯H⋯SiR_3_) have been proposed in zinc catalysed C–H silylation of (hetero)arenes.^34,35^

**Fig. 2:**
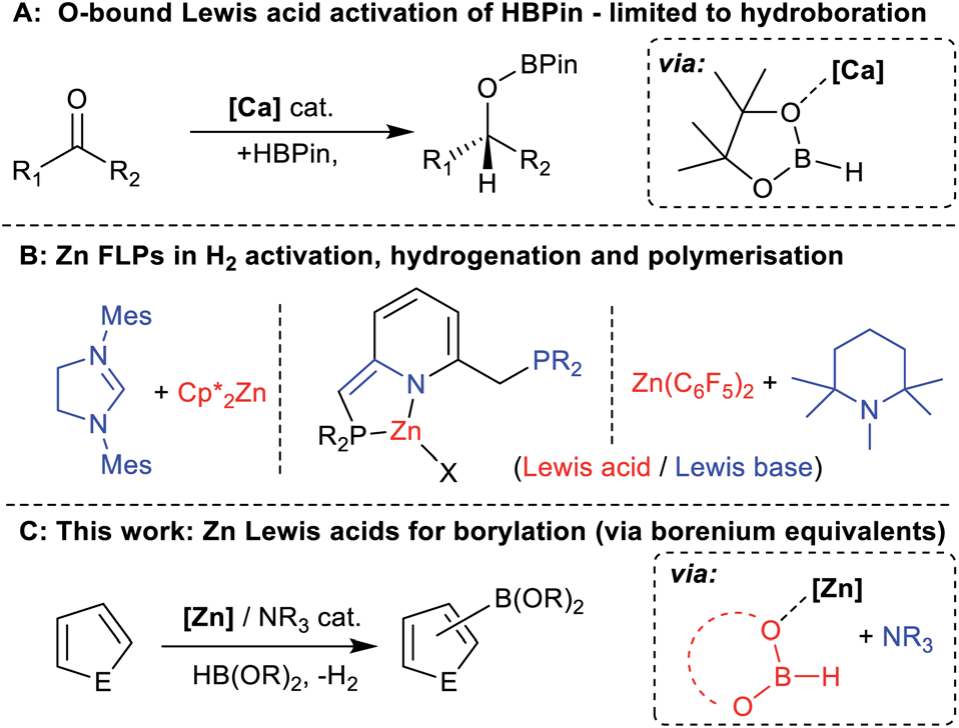
(A) Hydroboration mediated by oxophilic Lewis acids. (B) Previous work on zinc based FLPs. (C) This work, [Zn]/NR_3_ catalysed C–H borylation of a range of heteroarenes.

Hydroboranes, such as HBPin, have multiple Lewis basic sites, specifically the B–H and 2x O, thus Lewis acids also can coordinate at an oxygen of HBPin. Indeed, Lewis acid coordination to oxygen has been proposed in the hydroboration of ketones catalysed by highly oxophilic Lewis acids (*e.g.* Ca and Mg complexes, Fig. 2A).^36^ Regardless of binding site, to enable Lewis acid coordination to weakly Lewis basic hydroboranes requires exclusion of significantly stronger Lewis bases. However, bases often play a crucial role in facilitating deprotonation steps during C–H borylation using main group electrophiles.^23,26^ Therefore, identifying a Brønsted base that when combined with a zinc Lewis acid functions as a FLP maybe essential to facilitate C–H deprotonation and ultimately borylation (particularly given the lack of precedence for Zn–H units acting as effective Brønsted bases towards heteroarenes).^32^ While some notable work on zinc based FLPs has been reported, their use to date has been limited (Fig. 2B),^37^ and they have not been applied in arene C–H functionalisation to our knowledge.

Herein we present the first, to our knowledge, report of zinc complexes that catalyse the C–H borylation of heteroarenes using HBPin and HBCat. This process utilises NHC-supported cationic zinc complexes. Mechanistic studies are consistent with C–H borylation proceeding through coordination of a zinc Lewis acid to an oxo site of a hydroborane to generate a borenium equivalent. When combined with the optimal base this leads to a catalytic C–H borylation methodology applicable to less activated heteroarenes (*e.g.* thiophenes) than normally observed using main group (redox inactive) catalysts.

## Results and discussion

### Borylation with pinacolborane (HBPin)

**Fig. 3:**
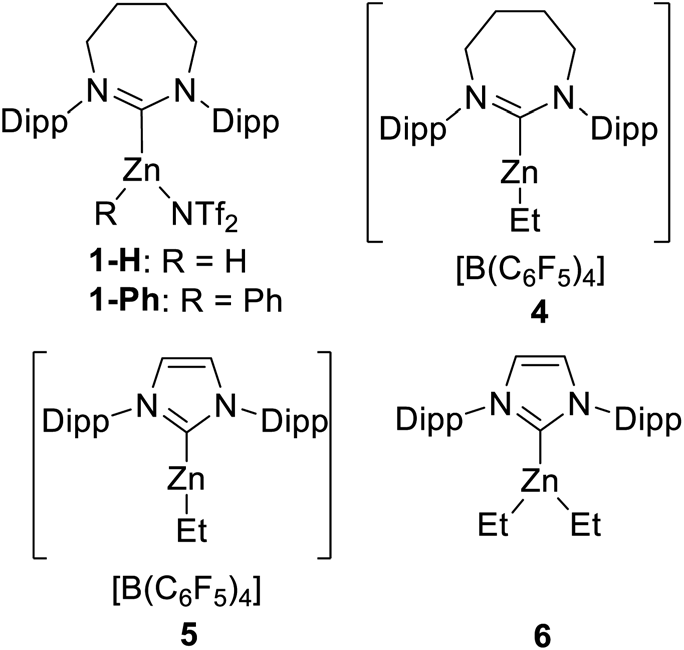
Complexes **1**, **4–6**.

Due to its efficacy in the C–H borylation of terminal alkynes,^31^ 7DippZnH(NTf_2_) (**1-H**, Fig. 3) was trialled for catalysing heteroarene C–H borylation (7Dipp = 1,3-bis(2,6-diisopropyl-phenyl)-4,5,6,7-tetrahydro-1,3-diazepin-2-ylidene). N–Me–Indole (**2a**) was chosen as the initial substrate due to its high nucleophilicity (*N* = +5.75 on the Mayr nucleophilicity index) and high reactivity in S_E_Ar reactions.^38^ The stoichiometric reaction of **1-H** (generated *in situ* from **1-Ph** and HBPin)^31^ with **2a** and HBPin led to C–H borylation (by ^1^H and ^11^B NMR spectroscopy), with effectively full conversion of **2a** to the C3 borylated indole (**3a**) at 100 °C (Table 1, entry 1). ^1^H NMR spectroscopy confirmed the presence of **1-H** in the reaction mixture post full consumption of **2a** indicating the feasibility of turnover. At 10 mol% loading of **1-Ph** 78% borylation of **2a** in chlorobenzene (PhCl) was observed over 36 h (entry 2).

**Table 1:** Catalyst optimization

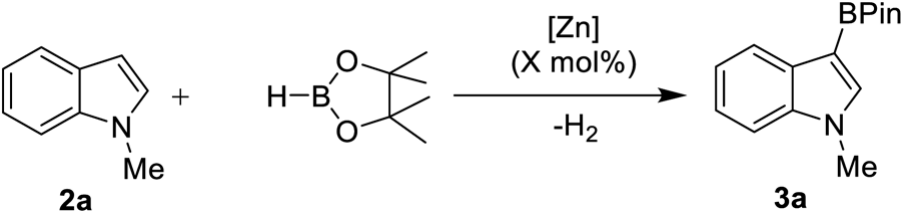						
Entry	[Cat]	Catalyst loading/mol%	*T*/°C	Solv.	Time/h	Conv.^a^/%
1	**1-H**	100	100	PhCl	18	100
2	**1-Ph**	10	100	PhCl	36	78^b^
3	**4**	10	80	PhCl	36	47
4	**5**	10	80	PhCl	18	97
5	**5**	10	80	C_6_D_6_	18	80
6	**5**	10	80	C_7_H_8_	18	91
7	**5**	5	80	PhCl	18	94
8	**5**	5	80	PhCl	10	82
9	**6**	10	80	PhCl	18	0
10	ZnCF^c^	5	80	PhCl	18	0
11	—	—	80	PhCl	18	0
12	**5**	5	80	PhCl	18	62^d^
13	**5**	5	80	PhCl	10	92^e^
14	**[8]+** f	10	80	PhCl	18	0
15	LBH_3_^g^	5	80	PhCl	18	0
16	IDipp	10	80	PhCl	18	0

a Conversions estimated *in situ* by ^1^H NMR spectroscopy of diagnostic product resonances *versus* those of N–Me–indole.

b 2.3 equivalents of HBPin used.

c ZnCF = Zn(C_6_F_5_)_2_.

d With 1 equivalent of DBP added.

e With 5 mol% of *N*,*N*-dimethyl-*p*-toluidine added.

f [**8**][B(C_6_F_5_)_4_] was generated *in situ* before addition of HBpin/**2a**.

g L = THF used as a 1 M THF solution.

As NTf_2_ is known to coordinate to NHC–zinc cations in haloarene solvents we decided to probe anion effects.^31^ The methodology of Dagorne and co-workers^39^ was utilised to access [7DippZnEt][B(C_6_F_5_)_4_] (**4**) and [IDippZnEt][B(C_6_F_5_)_4_] (**5**) (Fig. 3) by alkyl abstraction using [CPh_3_][B(C_6_F_5_)_4_] from the respective NHCZnEt_2_ precursors. Compound **5** has been shown previously by Dagorne *et al.* to be active in catalytic hydrosilylation with reactivity presumably proceeding *via* [Zn]⋯H⋯SiR_3_ species that react as silicon based electrophiles.^39^ Assessment of **4** and **5** as (pre)catalysts showed that both effect catalytic C–H borylation and are more active than the NTf_2_ analogue, with compound **5** providing the better outcome (entries 3 and 4). Further testing showed that changing the solvent to C_6_D_6_ or toluene using **5** had minimal effect (entries 5 and 6), whilst the loading of **5** could be decreased to 5 mol% with high yielding borylation still observed (entries 7 and 8). Use of the neutral complex IDippZnEt_2_ (**6**) as a catalyst showed that the cationic nature of **5** is important for catalytic borylation (entry 9). While use of a NHC free, neutral zinc Lewis acid, Zn(C_6_F_5_)_2_, also led to no borylation of **2a** (entry 10). In this case significant Zn–C/H–B metathesis (to form C_6_F_5_–BPin) was observed along with the precipitation of an insoluble solid, presumably ZnH_2_. Finally, in the absence of a zinc catalyst no borylation was observed (entry 11).

**Fig. 4:**
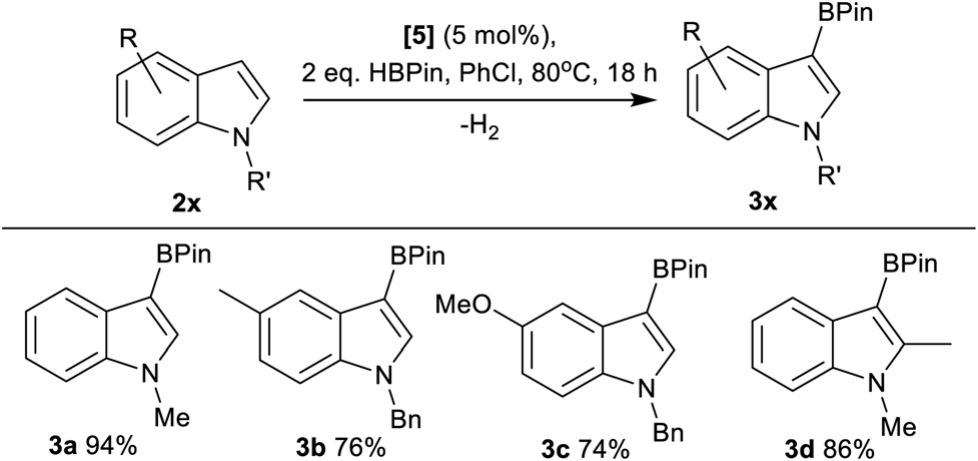
C3–H borylation, yields by NMR spectroscopy *versus* an internal standard.

Next, the scope of borylation using **5** was surveyed, and found to be limited to activated indoles (*e.g.***3a–3d**, Fig. 4), with unactivated indoles (*e.g.* brominated indoles) not borylated. It should be noted that products from the reduction of **2x**, N–R–indolines, were also observed by NMR spectroscopy as minor by-products in a number of cases alongside formation of **3x**. The borylation of other less nucleophilic substrates using **5**/HBPin was unsuccessful, with borylation not observed for anisole, 2-methylthiophene and 2-methylfuran under these conditions. Furthermore, N–Me–pyrrole, 3,4-ethylene-dioxythiophene and 6-fluoro-N–Bn–indole gave only 5%, 14% and 4% borylation, respectively, after 18 h at 80 °C.

**Scheme 1:**
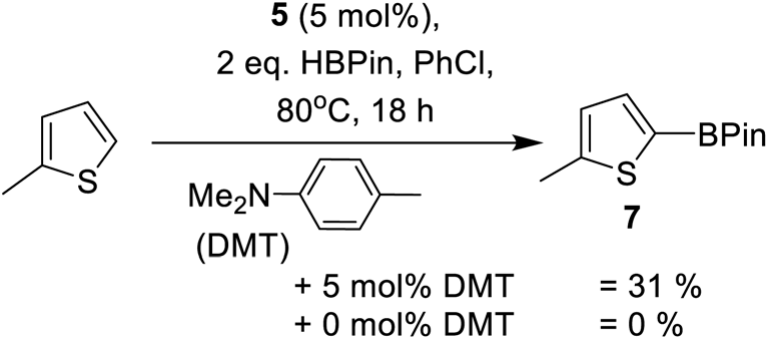
DMT dependent borylation of 2-methylthiophene with HBPin.

**Scheme 2:**
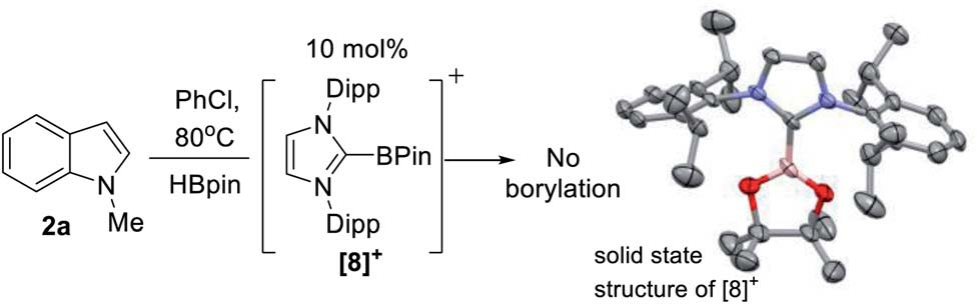
Left) No borylation observed using [IDippBPin]^+^. (Right) The structure of the cationic portion of [**8**][NTf_2_], ellipsoids at 50% and H atoms omitted.

Other main group catalysed arene C–H borylation reactions are facilitated by addition of a Brønsted base that deprotonates the arene in a concerted or stepwise S_E_Ar mechanism.^23,26^ Indeed, as noted by Oestreich and co-workers, the *in situ* formation of indolines during indole borylation may well be vital in enabling catalytic C–H borylation of indoles.^25*b*^ This is potentially due to indolines being stronger Brønsted bases (than indoles) and thus enable access to a FLP type borylation mechanism.^23^ Therefore we investigated basic additives in this C–H borylation reaction. The addition of a hindered base, 2,6-di-*tert*-butyl-4-methyl-pyridine (DBP), to preclude any Zn ← N_py_ bond formation, was found to reduce the conversion of **2a** to **3a** (Table 1, entry 12). This is attributed to DBP sequestering a proton from an S_E_Ar reaction forming a weaker Brønsted acid (relative to that otherwise present during catalytic electrophilic borylation). This is hypothesised to slow a dehydrocoupling step of [Base-H]^+^ with a Zn–H or a B–H species (forming H_2_) that leads to regeneration of an on-cycle electrophile. In contrast, the addition of the weaker base *N*,*N*-dimethyl-*p*-toluidine (DMT, Scheme 1) at a 1 : 1 ratio with respect to **5** led to an acceleration in the borylation of **2a** (Table 1, entry 8 *vs.* 13). In addition, this combination enabled the borylation of the less nucleophilic substrate 2-methyl thiophene with formation of 31% of **7** (Scheme 2) after 18 h. In contrast, no formation of **7** was observed in the absence of DMT (by ^1^H NMR spectroscopy). This confirms the importance of DMT, presumably functioning as a Brønsted base during the borylation process. Furthermore, it is a rare example of a main group electrophile catalysing the C–H borylation of a less activated heteroarene, such as 2-methyl thiophene (*N* = +1.35),^40^ using HBPin directly.^23^ However, the low yield of **7** after 18 h with **5**/DMT indicates a limit in the heteroarene nucleophiles viable for borylation using this system. Thus mechanistic insight was sought to enable expansion of scope.

### Mechanistic studies

The first key question to answer was if the catalysis was Zn mediated or simply Zn initiated (*e.g.* forming a boron electrophile which is an on-cycle species, *e.g.* a [PinB(base)]^+^/base FLP).^23^ It should be noted that while [B(C_6_F_5_)_4_]^−^ is used as the counterion in **5**, no decomposition of this anion to B(C_6_F_5_)_3_ (which can catalyse electrophilic C–H borylation and silylation) was observed based on ^19^F and ^11^B NMR spectroscopy.^41^ Furthermore, C–H borylation also proceeded with NTf_2_ as the counterion (*e.g.* with **1-H**), disfavouring a process mediated by B(C_6_F_5_)_3_. At this point in the study a zinc Lewis acid acting as a hydride acceptor enabling formation of a borenium cation could not be precluded. Indeed, crystals of borenium ion [IDippBPin][NTf_2_] ([**8**][NTf_2_]) were isolated from one catalytic reaction mixture, albeit in a small amount. This indicates that borenium electrophiles can be formed under catalytic conditions (it should be noted [PinB(L)]^+^ boreniums are relatively weak hydridophiles, thus hydride transfer from PinBH(L) to a zinc cation is feasible – *vide infra*).^42^**[8]+** is presumably formed by NHC dissociation from zinc, NHC coordination to HBPin, followed by hydride abstraction from (NHC)HBPin – all reported steps.^31,43^ The structure of **[8]+** (Scheme 2, right) is unremarkable compared to [(NHC)BCat]^+^,^44^ excluding shorter B–O bonds in **[8]+** due to the improved π donor ability of pinacol relative to catechol. Importantly, a control reaction using 10 mol% of **[8]+**, generated *in situ* from (IDipp)HBPin/B(C_6_F_5_)_3_ (by ^19^F/^11^B NMR spectroscopy), as catalyst led to no borylation of **2a** with HBPin under identical catalytic conditions (table 1 entry 14). Furthermore, 5 mol% BH_3_-THF (1 M in THF, entry 15), and 10 mol% of IDipp (entry 16), as potential initiators led to no borylation of **2a** under the catalytic conditions.

This indicates a zinc complex is an on cycle species, with a [IDippZnH]^+^ species proposed to be key using **5**. This is due to metathesis of the Zn–Et unit in **5** with HBPin being rapid relative to C–H borylation (formation of EtBPin occurs before formation of significant amounts of **3a**). However, despite numerous attempts, in our hands formation of an isolable [NHCZnH][B(C_6_F_5_)_4_] species from combinations of **5** (or **4**) with HBPin (and *via* other routes) proved elusive. Formulation as a zinc hydride was supported by studies combining **5** with HBPin and DBPin; using HBPin new singlets grow in as metathesis proceeds at 2.7 and 1.5 ppm in the ^1^H NMR spectrum, tentatively assigned as Zn–H. Notably, these resonances are not observed in the ^1^H NMR spectrum when using DBPin, although the by-product from metathesis, EtBPin, is present. The putative hydride chemical shifts are comparable to other cationic NHC–ZnH species in which hydrides are bridging two zinc centres, suggesting an oligomeric structure for putative [IDippZnH]^+^.^45^

**Scheme 3:**
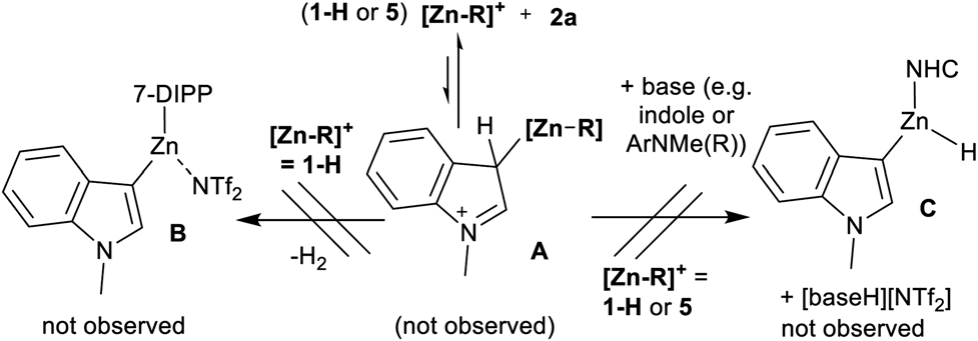
Attempted metalation reactions between [Zn-R]^+^ and **2a**.

Zinc catalysed C–H borylation can proceed *via* a Zn electrophile interacting with **2a** or HBPin. With the former, this would form a species related to **A** (Scheme 3). In this the Brønsted acidity of the indole–C3 proton will be enhanced.

Deprotonation (by Zn–H or another base, *e.g.* N–Me–indoline) would form a zinc indolyl complex (*e.g.***B** or **C**). Subsequent metathesis with HBPin then would generate the indole boronic ester, **3a**. With no Zn–H species derived from **5** isolable in our hands **1-H** was used in stoichiometric reactions. Combining **1-H** with **2a** led to no C–H metalation, even under forcing conditions. Furthermore, combining **5**, **2a** and DMT (1 : 1 : 1) also led to no C–H zincation of **2a** on heating to 80 °C. These observations disfavour a borylation mechanism in which activation of N–Me–indole occurs first by interaction with a Lewis acidic zinc-species.

**Scheme 4:**
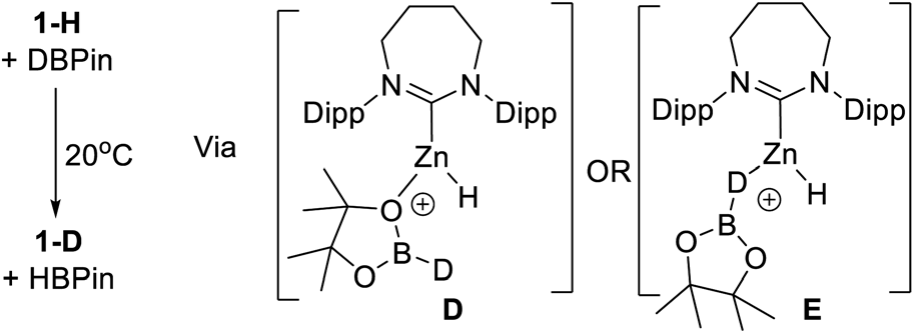
H/D exchange at room temperature *via* possible intermediates **D** or **E**.

It has been previously observed that **1-H** reacts with HBPin on heating to give unidentified zinc containing species,^31*b*^ indicating an interaction between a NHC–Zn Lewis acid and HBPin can occur. To probe the interaction between **1-H** and HBPin further, **1-H** was reacted with DBPin which led to H/D scrambling at room temperature. The Zn–H resonance of **1-H** (3.66 ppm) decreases in intensity with the concurrent appearance of a singlet resonance, assigned as Zn-D in **1-D**, in the ^2^H NMR spectrum (3.69 ppm). Additionally, the ^11^B NMR spectrum now showed two species, one assigned as H-BPin (doublet) and the other D-BPin (broad singlet). This confirms the borane and complex **1-H** interact at room temperature. Hydrogen scrambling between **1-H** and HBPin can feasibly occur *via* H–B or O- bound isomers (*e.g.***D** or **E**, Scheme 4).

**Scheme 5:**
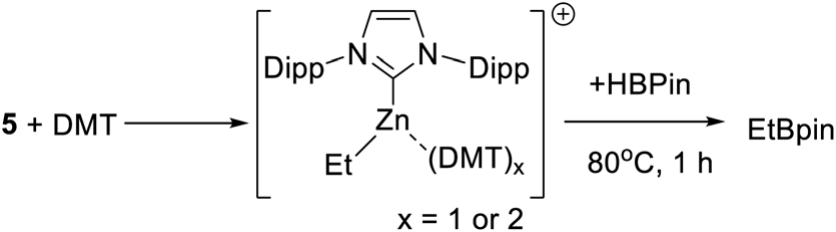
Metathesis of Zn–C/H-BPin in the presence of DMT.

As C–H borylation is more effective in the presence of DMT the reactivity of DMT towards **5** also was explored. The addition of varying equivalents (from 0.5 to 2 equivalents) of DMT to **5** led to a shift in the ^1^H resonances of both, indicating an interaction between DMT and the zinc centre. The NMR data was consistent with formation of the Lewis adduct [5-(DMT)_*x*_][B(C_6_F_5_)_4_] (*x* = 1 or 2, with free and bound DMT in rapid exchange on the NMR timescale), however, this compound could not be isolated in our hands. Notably, the presence of DMT does not prevent reactivity with HBPin, heating a 1 : 1 **5**/DMT mixture with HBPin for 1 h at 80 °C led to complete H–B/Zn–C metathesis (Scheme 5) as judged by consumption of all Zn–Et resonances and formation of EtBPin (by NMR spectroscopy). This indicates that [IDippZnEt]^+^/DMT combinations permit interaction of the Zn centre with HBPin, either *via* displacement of DMT or *via* higher coordinate zinc species. No borocations of general formula [PinB(DMT)_*n*_]^+^ (*n* = 1 or 2) were observed in mixtures of **5**/DMT/HBPin (by ^11^B NMR spectroscopy).

**Scheme 6:**
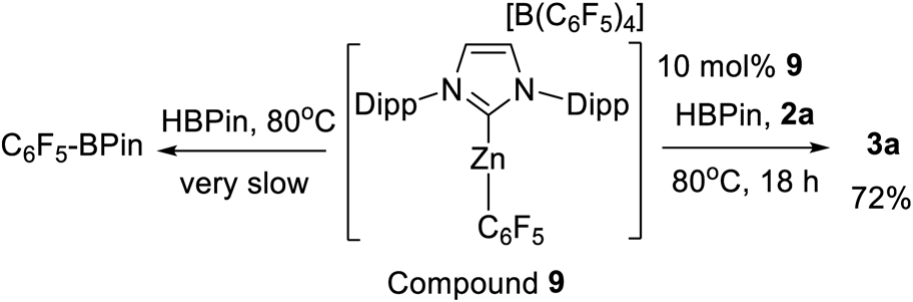
Left) **9** undergoes slow metathesis with HBPin. (Right) C–H borylation catalysed by **9**.

The failure to observe any C–H zincation of N–Me–indole with **1-H** suggested that a Zn–H moiety may not be required for borylation catalysis. Instead borylation may proceed by an electrophilic borenium equivalent, such as **D** or **E** (Scheme 4). To investigate this hypothesis the reported complex [IDippZnC_6_F_5_][B(C_6_F_5_)_4_] (**9**, Scheme 6) was synthesized from **5** by Et/C_6_F_5_ exchange using B(C_6_F_5_)_3_.^39^ Compound **9** was selected as the C_6_F_5_ group both enhances electrophilicity at zinc (relative to **5**) and dramatically retards the rate of Zn–C/H–B metathesis using HBPin. Using 10 mol% of **9** as catalyst 72% conversion of **2a** to **3a** was observed after 18 h at 80 °C. In contrast to **5**, Zn–C_6_F_5_/H-BPin metathesis is very slow (by ^11^B and ^19^F NMR spectroscopy), with **9** the major zinc species persisting in solution for all of the 18 h under catalytic conditions. As Zn–C/H–B metathesis from **9** would form an identical Zn–H complex to that derived from metathesis of **5** this should give a much lower borylation conversion (at the very low level of Zn–C_6_F_5_/H-BPin metathesis observed for **9**) if only a Zn–H species is active and **9** is inactive for borylation. Therefore this indicates that **9** is active and that catalytic borylation does not require a Zn–H species.

Compound **9** also formed an observable (by NMR spectroscopy) adduct with HBPin. Mixtures of HBPin and **9** resulted in significant broadening of the normally well resolved doublet of HBPin in the ^11^B NMR spectrum. Shifting of the Zn–C_6_F_5_ resonances in the ^19^F NMR spectrum was also observed. Analysis by variable temperature NMR spectroscopy led to the observation of two distinct sets of Zn–C_6_F_5_ resonances at low temperature. In contrast, cooling **9** in the absence of HBPin led to no additional resonances in the ^19^F NMR spectrum, confirming that the new resonances are due to an interaction between **9** and HBPin. Combined these results support an interaction between HBPin and a cationic NHC–Zn species, such as **1-H** or **9**, as the key initial step in C–H borylation.

As discussed above, the interaction of HBPin with NHC–Zn complexes could be as a B–H–[Zn] or a B–O–[Zn] unit (*e.g.***D** or **E**, Scheme 4). Following Hall's approach to probe for O bound Lewis acid activation in crotylboration,^46^ the hydroborane 9-borabicyclo(3.3.1)nonane (HBBN) was used in place of HBPin. Notably, this led to no borylation despite Zn–C/H-BBN metathesis from **5** occurring (based on formation of EtBBN by ^11^B NMR spectroscopy). This suggests that an H-bound interaction (*e.g.* [Zn]–H–B) may not be energetically accessible or not be productive for borylation in this system and that an O-Lewis basic site in the hydroborane may be essential. To further probe the oxophilicity of the zinc Lewis acids the Gutmann-Beckett method was used.^47^ Measurements by Dagorne and co-workers^39^ showed that low coordinate NHCZn cations display considerable Lewis acidity toward Et_3_PO. Consistent with this, **1-H** and **1-Ph** induced a larger downfield ^*δ*31^P shift compared to ZnPh_2_ (Table 2, entries 1, 3 and 4), with **1-H** more Lewis acidic towards Et_3_PO than **1-Ph**. However, while both **1-Ph** and **1-H** had a greater *δ*Δ^31^P than [IDippZnMe][B(C_6_F_5_)_4_] (entry 2), they were less Lewis acidic (based on the *δ*Δ^31^P value) than **9**, which itself is less Lewis acidic based on this scale than B(C_6_F_5_)_3_ (entries 5 and 6).

**Table 2:** Δ*δ*^31^P chemical shift of Et_3_PO on addition of electrophiles

Entry	Complex	Δ*δ*^31^P{^1^H}/ppm
1	ZnPh_2_	12.2
2	[IDippZnMe][B(C_6_F_5_)_4_]	17.2^a^
3	7DippZnPhNTf_2_ (**1-Ph**)	19.7
4	7DippZnHNTf_2_ (**1-H**)	22.0
5	[IDippZnC_6_F_5_][B(C_6_F_5_)_4_] (**9**)	24.7
6	B(C_6_F_5_)_3_	26.4

a Data from Dagorne and co-workers.^39^

**Scheme 7:**
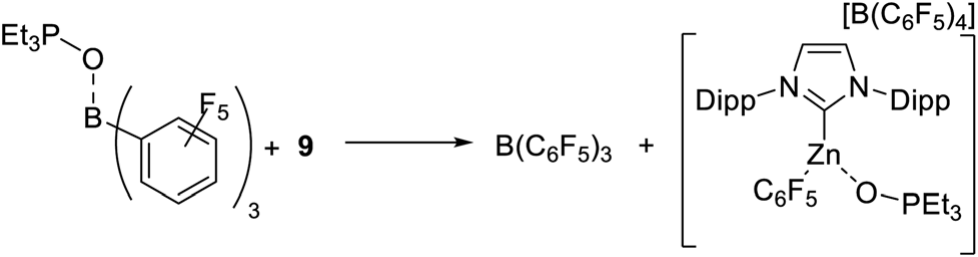
Competition experiments between B(C_6_F_5_)_3_ and **9** for the binding of Et_3_PO.

The fact that HBPin/B(C_6_F_5_)_3_ mixtures do not lead to the borylation of highly nucleophilic arenes (such as N–R–indoles) in contrast to mixtures of HBPin with **1-H** or **9**, suggested a weaker interaction/activation of HBPin by B(C_6_F_5_)_3_. The latter however is inconsistent with the relative Δ*δ*^31^P values from Et_3_PO binding. To more accurately assess the relative Lewis acidity of **9** and B(C_6_F_5_)_3_ towards Et_3_PO stoichiometric competition reactions were performed. These were undertaken due to the inherent limitations of the Gutmann-Beckett method, *e.g.* for comparing the Lewis acidity of disparate species (such as boron based *vs*. zinc based Lewis acids).^48,49^ Notably, adding **9** to Et_3_PO–B(C_6_F_5_)_3_ led to complete Et_3_PO transfer, *i.e.* formation of free B(C_6_F_5_)_3_ and the Et_3_PO adduct of **9** (by ^11^B, ^19^F and ^31^P NMR spectroscopy, Scheme 7). As expected the reverse reaction, adding free B(C_6_F_5_)_3_ to **9**-OPEt_3_, led to no reaction (by NMR spectroscopy). This indicates that the binding of Et_3_PO to **9** is thermodynamically preferred over binding to B(C_6_F_5_)_3_. This is in contrast to the Lewis acidity ordering predicted by the *δ*Δ^31^P values and emphasises that caution has to be taken applying the Gutmann-Beckett method to assess disparate Lewis acids.

**Fig. 5:**
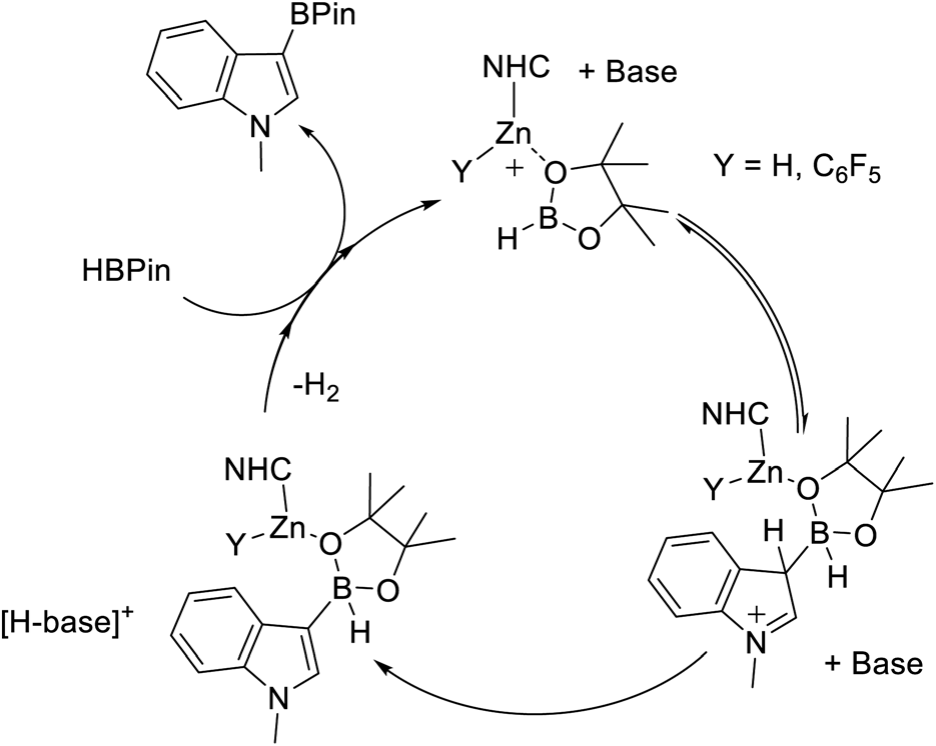
Proposed mechanism proceeding *via* O-bound HBPin. Base is shown non-coordinated to zinc throughout, it is feasible that it is coordinated to zinc at various points in the proposed cycle.

The observation of greater oxophilicity for **9** relative to B(C_6_F_5_)_3_ still does not preclude a Zn–H-BPin (*e.g.***E**) intermediate being key during borylation. To gain insight into the Lewis acidity towards hydride of these zinc cations, **5** and **9** were combined separately with [NEt_4_][HB(C_6_F_5_)_3_]. This led to no reaction in both cases. This is not attributed to hydride transfer inhibition by steric bulk (*i.e.* a kinetic barrier), as the closely related reaction between **9** and Et_3_PO–B(C_6_F_5_)_3_ occurs rapidly. Instead it indicates a lower hydridophilicity for these NHC–zinc cations relative to B(C_6_F_5_)_3_. Combined this data suggests that the greater Lewis acidity of the NHC–Zn cations utilised herein towards oxo-Lewis bases (relative to B(C_6_F_5_)_3_) leads to stronger binding to HBPin presumably through an oxygen site. A strong interaction between Zn and HBPin can be expected to lead to a significant enhancement of Lewis acidity at boron in species such as **D**. Thus we propose that the active electrophile is **D** (or a closely related species), which reacts with a heteroarene, and aided by a base forms a C–B bond. Subsequently multiple steps, *e.g.* dehydrocoupling and RBPin for HBPin exchange, would ultimately lead to C–H borylation (Fig. 5). If an O-bound borane–Zn adduct is a key intermediate then alteration of the borane structure will impact the Lewis acidity of the key electrophile (*e.g.***D**) and modulate the C–H borylation scope – provided a [Zn]–O–B adduct is still formed.

### Borylation with catecholborane (HBCat)

**Scheme 8:**
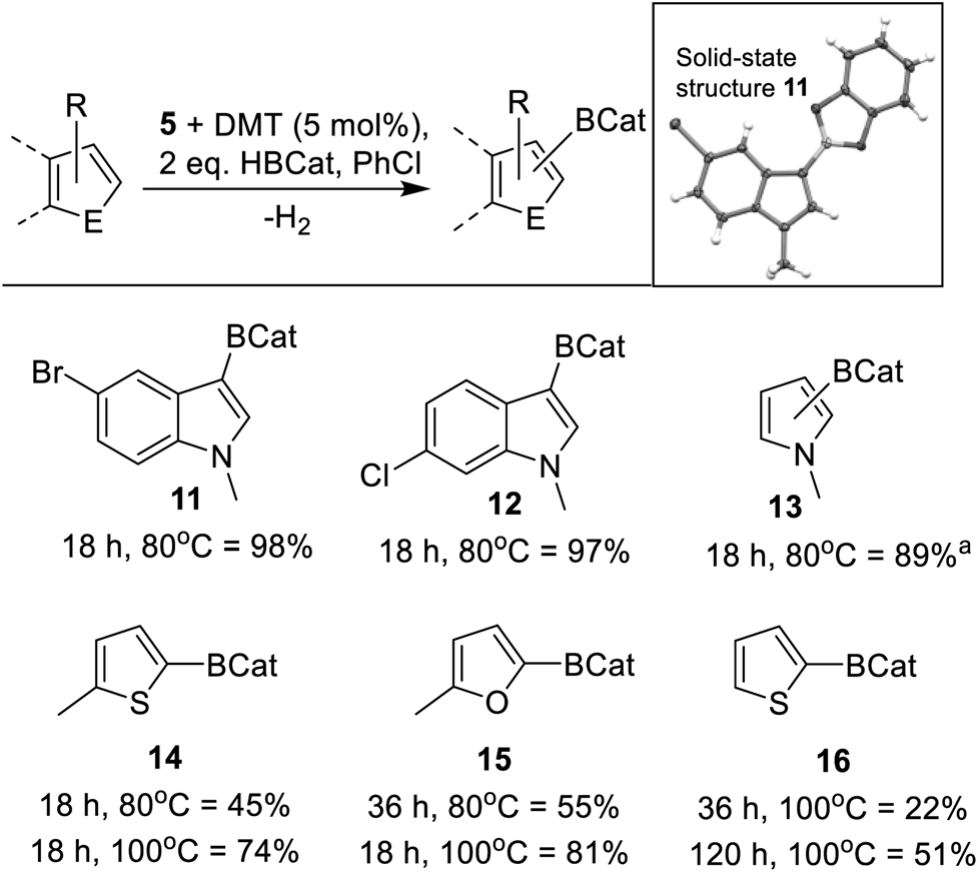
Substrate scope using **5**/DMT in borylation with HBCat (yields using dibromomethane as an internal standard). Inset top, solid state structure of **11** shown with 50% ellipsoid probability. *a* = *a* mixture of the 2-BCat and 3-BCat isomers were formed in a 73 : 16 ratio.

Based on the mechanistic studies the borane HBPin was replaced with HBCat. The use of HBCat increased the substrate scope, with less activated indoles, such as halogenated indoles and N–Me–pyrrole, now undergoing C3–H borylation to form **11–13** (Scheme 8) in excellent yield using 5 mol% of both **5** and DMT. It should be noted that the use of DBP also retarded catalysis with HBCat, while DMT resulted in an improved outcome relative to the base free system. Furthermore, increasing the loading of DMT from 5 mol% to 10 mol% (while keeping **5** at 5 mol%) led to no improvement in the borylation of 2-methyl-thiophene, thus all borylation reactions using HBCat are performed using 5 mol% of both **5** and DMT. With 2-methyl thiophene the use of HBCat resulted in increased conversion relative to that using HBPin, with **14** formed in 45% after 18 h at 80 °C. The use of longer reaction times and/or higher temperature led to greater conversion (Scheme 8). Using 5 mol% **5**/DMT other less activated substrates, such as 2-methyl furan and even thiophene (Mayr *N* value = −1.01) were borylated with HBCat to form **15** and **16**, respectively (Scheme 8). While thiophene only underwent borylation slowly, it is still a rare example of a significantly less nucleophilic heteroarene undergoing borylation with a main group catalyst. To our knowledge, all other main group catalysts reported to date only borylate activated (by substitution with an electron donating group) thiophenes.^23^ Finally, it should be noted that in stoichiometric reactions reported to date [CatB(amine)]^+^ does not borylate thiophene.^50^ This highlights the enhanced borylating reactivity of this zinc mediated borylation process.

Mechanistically, we propose an analogous process to that discussed for HBPin (Fig. 5), involving binding of a NHC–Zn cation to the oxygen of HBCat, forming a borenium equivalent, that reacts with the heteroarene. Deprotonation by an amine base will then be followed by dehydrocoupling and RBCat/HBCat exchange to complete the cycle. The zinc species is presumably an NHCZn–H cation as combination of **5** (or **9**) with HBCat led to rapid metathesis (as indicated by the rapid formation of EtBCat (or C_6_F_5_BCat) by ^11^B NMR spectroscopy). While no [CatB(DMT)]^+^ species were observed *in situ* (by ^11^B NMR spectroscopy) their presence at low concentration cannot be precluded. Therefore we compared the **5**/DMT system to borylation catalysed by [CatB(DMT)]^+^ borenium ions under identical conditions. 5 mol% of B(C_6_F_5_)_3_ and DMT were combined with HBCat (conditions that have been shown to generate borocations that effect borylation),^25*b*^ and after 36 h at 80 °C in chlorobenzene **14** had formed in only 32% yield. In contrast, under identical conditions, 5 mol% **5**/DMT led to 74% formation of **14**. The considerably greater conversion with **5**/DMT further confirms that the borylating system derived from **5**/DMT/HBCat is more reactive under identical conditions than that using [CatB(DMT)][HB(C_6_F_5_)_3_].

### DFT calculations

**Scheme 9:**
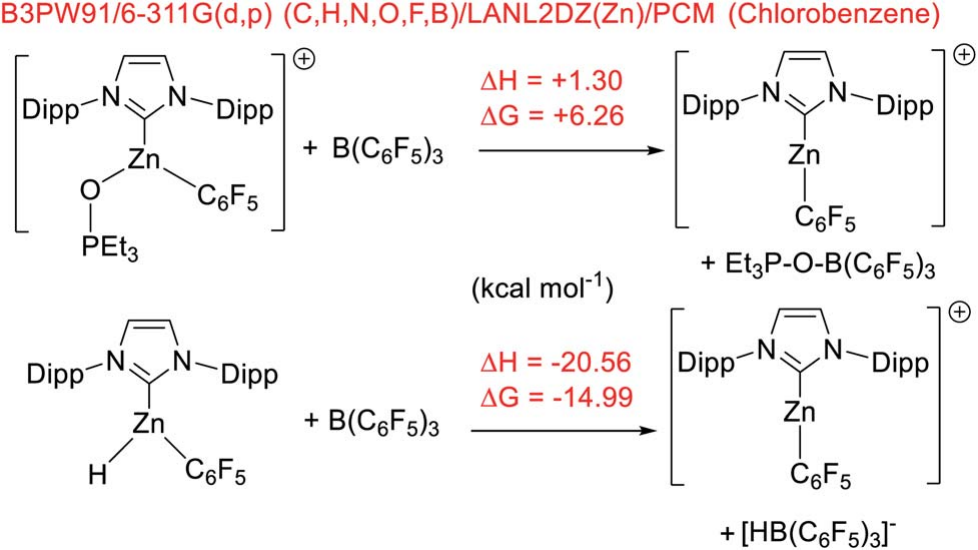
Relative Lewis acidity of **[9]+** and B(C_6_F_5_)_3_ towards Et_3_PO and hydride.

To gain insight into hydroborane activation by the zinc cations discussed above, DFT calculations were conducted. These were initially performed at the M06-2x/6-311G(d,p)/lanl2dz(Zn)/PCM (PhCl) level (PCM = polarizable continuum model), a combination used in our recent work to allow direct comparison to previously reported NHC–Zn cations.^31,51^ While this level has proved accurate for calculations on p block compounds, Truhlar highlighted the shortcoming of the M06-2x functional when used for transition metal complexes.^52^ Thus, M06-2x and two other functionals were assessed using two benchmark reactions, the relative (to B(C_6_F_5_)_3_) affinity of **[9]+** towards Et_3_PO and towards hydride. Notably, calculations using the M06-2x functional gave **[9]+** a lower Lewis acidity towards Et_3_PO than B(C_6_F_5_)_3_. This is in contrast to the experimental observations and to relative Lewis acidity based on calculations using the B3LYP-D3BJ and B3PW91 functionals, which both correctly identify **[9]+** to be more Lewis acidic towards Et_3_PO than B(C_6_F_5_)_3_. The three functionals all gave the correct relative Lewis acidity towards hydride (Scheme 9). Based on these observations and its recent utility in other zinc-cation catalysed processes,^32^ B3PW91 was used for further calculations, and only results with this functional are discussed.

**Scheme 10:**
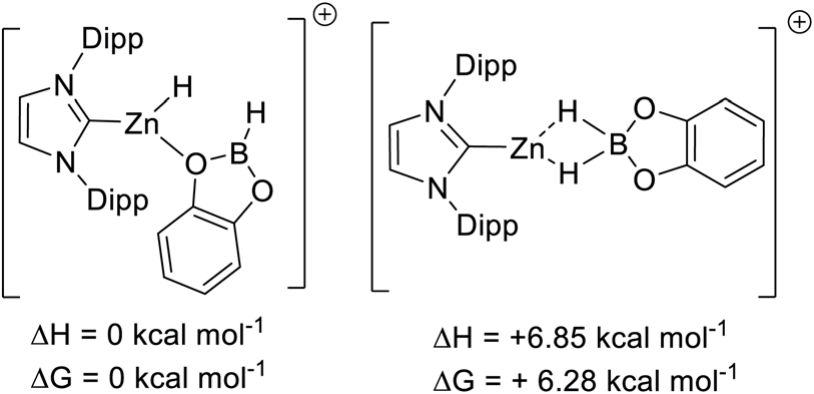
Relative energy of O and H bound CatBH–[IDippZnH]^+^.

Using B3PW91, **[9]+** is a considerably weaker Lewis acid toward hydride than B(C_6_F_5_)_3_, by Δ*G* = 15 kcal mol^−1^. This combined with the experimental data on the lower hydridophilicity of these zinc cations is notable. Previous work has shown that the Lewis acidity towards hydride is comparable for B(C_6_F_5_)_3_ and [CatB(amine)]^+^ species.^53^ Thus using the zinc cations utilised herein to remove hydride from CatBH(amine) will be highly endergonic, disfavouring the presence of [CatB(amine)]^+^ borenium cations in these borylation reactions. Instead, the significant oxophilicity of the zinc cations arising from the unit positive charge and the lower electronegativity of zinc (relative to boron) appears crucial. Consistent with significant oxophilicity, the putative Zn cation formed by metathesis of **5** with HBPin or HBCat, [(IDipp)ZnH]^+^, was found to favour binding of HBCat *via* O in preference to H–B (even though in the latter both hydrides are interacting with B and Zn, Scheme 10 top). When HBPin was optimised interacting with the same zinc cation only an O bound isomer was found.

**Scheme 11:**
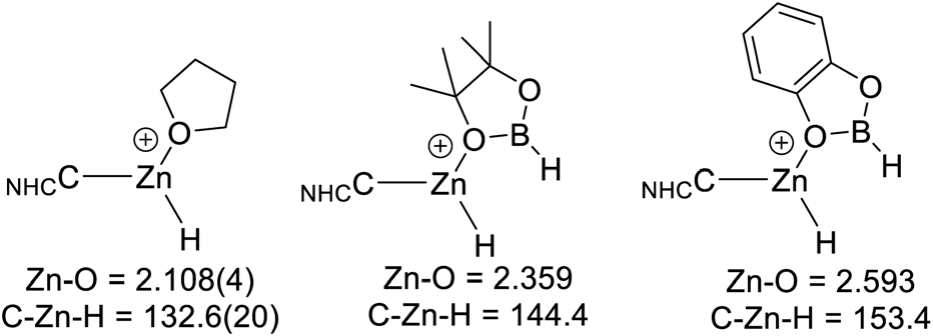
Key metrics for oxygen Lewis bases bound to [NHCZnH]^+^, the metrics for [7-DippZnH(THF)]^+^ are from ref. 31. Distances in Å, angles in (°).

Comparison of the calculated O-bound borane structures to that of the previously reported solid state structure of [(7-Dipp)ZnH(THF)]^+^, indicate that the hydroboranes interact more weakly with the cationic zinc centre than THF (Scheme 11). This is consistent with the weaker Lewis basicity of boranes relative to THF due to the delocalisation of oxygen electron density onto the boron centre. As expected, catecholborane is a weaker base relative to pinacolborane (based on the larger Zn–O distance and C–Zn–H angle, with the latter closer to the 180° angle expected for a two coordinate [NHCZnY]^+^ system).^39^

The impact of the hydroborane interacting with the zinc centre can be observed in both the metrics of the hydroborane (B–O and C–O bonds involving the O bound to zinc are elongated relative to those involving the non-bound O), and the orbital energies. For example, the energy of the LUMO with significant B character in free HBPin and free HBCat is: +0.835 eV (LUMO) and −0.709 eV (LUMO), respectively. On binding to [(IDipp)ZnH]^+^ the corresponding B based LUMO is found at −0.515 eV (LUMO+5) and −1.204 eV (LUMO+4), for zinc bound HBPin and HBCat, respectively (the lower energy unoccupied orbitals are NHC based in these adducts). The greater drop in energy for the boron based LUMO with HBPin (Δ*E* = 1.35 eV) on binding to zinc, relative to HBCat (Δ*E* = 0.495 eV), is presumably due to the stronger interaction of HBPin with the zinc centre (based on relative Zn–O distances). For the O-bound adduct of HBPin with **[9]+** the lowest energy boron based LUMO is found at −0.436 eV, indicating this effect on borane orbital energy is general for NHC–ZnR cations interacting with hydroboranes. The considerable drop in the energy of the HBPin based LUMO on binding is presumably a major factor in HBPin⋯Zn based boron electrophiles being able to borylate less nucleophilic heteroarenes, *e.g.* 2-methyl thiophene, to some extent. Combined, the data is consistent with O binding of hydroboranes to zinc cations generating borenium equivalents that are effective Lewis acids for heteroarene borylation.

## Conclusions

Cationic NHC–zinc complexes in combination with the appropriate Brønsted base are effective catalysts for the C–H borylation of heteroarenes using HBPin and HBCat. Notably, using the latter hydroborane extends main group catalysed electrophilic C–H borylation to weakly activated heteroarenes, *e.g.* thiophene, for the first time. Mechanistic studies indicate that (i) the zinc cations are highly oxophilic but much less hydridophilic, (ii) they activate the hydroborane and not the heteroarene, thereby generating borenium equivalents. Calculations indicate that this activation proceeds through binding of the zinc centre to an oxygen of the hydroborane and not the B–H, consistent with the greater oxophilicity of these zinc cations. The generated borenium cation functional equivalents are then proposed to effect C–H borylation. The use of oxophilic Lewis acid as activators for hydroboranes to effect (hetero)arene C–H borylation has been overlooked to date, and this new approach to catalysing (hetero)arene C–H borylation is currently under further investigation.

## Author contributions

M. E. G. performed the synthesis and characterised the compounds. K. Y. executed the theoretical calculations. G. S. N. solved the X-ray structures of the compounds. M. E. G. and M. J. I. designed the project, analyzed the results and wrote the manuscript.

## Conflicts of interest

There are no conflicts to declare.

## Supplementary Material

SC-012-D1SC01883C-s001

SC-012-D1SC01883C-s002
