# Supplementary material for: Zinc catalysed electrophilic C–H borylation of heteroarenes
Source: Chem Sci. 2021 May 13;12(23):8190–8. doi: 10.1039/d1sc01883c (PMC8208323; doi:10.1039/d1sc01883c)
Supplement: SC-012-D1SC01883C-s001 [file SC-012-D1SC01883C-s001.pdf]

Electronic Supplementary Material (ESI) for Chemical Science.
This journal is © The Royal Society of Chemistry 2021

Zinc catalysed electrophilic C-H borylation of heteroarenes

M. E. Grundy, K. Yuan, G. S. Nichol, M. J. Ingleson*

University of Edinburgh, Joseph Black Building, David Brewster Road, Edinburgh, EH9 3FJ

Table of Contents

S1. General information 2

S2. Synthesis of novel NHC-Zinc species 3

S3. Catalytic C-H Borylation 5

S4. Investigation of potential "trojan horse" catalysts 33

S5. Metathesis studies 37

S6. [IDippZnEt][B(C_6F_5)$_4$] / DMT study 48

S7. [IDippZnC_6F_5][B(C_6F_5)$_4$] / HBPin interaction study 52

S8. 7DippZnH(NTf_2) DBPin H/D exchange study 58

S10. Et_3PO competition experimentation 62

S11. Hydride affinity competition experimentation 64

S12. Isolation of borenium species [IDippBPin][NTf_2] 66

S13. X-Ray Crystallography 67

S14. DFT calculations 73

S15. Copies of NMR spectra for new compounds 118

S16. References 121

S1. General information

Unless otherwise stated, all the experiments were carried out under an inert atmosphere using either standard Schlenk techniques or in a MBraun glovebox (< 0.1 ppm H_2O / O_2). Chlorobenzene, d_6-benzene and d_5-bromobenzene were distilled over CaH_2 and stored over 3 Å molecular sieves. All other solvents were obtained from an Inert PureSolv MD5 SPS. Unless otherwise stated all chemicals were purchased from commercial sources and used as received. Trityl tetrakis(pentafluorophenyl)borate,[1] N,N-bis(2,6-diisopropylphenyl)-imidazol-2-ylidene (IDipp),[2] 1,3-bis(2,6-diisopropylphenyl)-4,5,6,7-tetrahydro-1*H*-1,3-diazepin-3-ium-2-ide (7Dipp),[3] 7DippZnPh_2,[4] 7DippZnPh(NTf_2),[4] 7DippZnH(NTf_2),[4] IDippZnPh_2,[4] IDippZnPh(NTf_2),[4] IDippZnEt_2,[5] [IDippZnEt][B(C_6F_5)$_4$],[6] [IDippZnC_6F_5][B(C_6F_5)$_4$],[6] 5-methoxy-N-benzylindole,[7] 5-methyl-N-benzylindole,[7] 5-fluoro-N-benzylindole,[8] 6-chloro-N-benzylindole[9] and 6-chloro-N-methylindole[10] were prepared according to reported literature procedures.

^{1}H, ^{2}H, $^{13}C\{^{1}H\}$, ^{11}B, ^{11}B, and ^{19}F NMR spectra were recorded on Bruker Avance III 400, Bruker Avance III 500MHz or Bruker PRO 500 MHz spectrometers. Chemical shifts are reported as dimensionless δ values and are frequency referenced relative to residual protio-solvent signals in the NMR solvents for ^{1}H and $^{13}C\{^{1}H\}$, while ^{11}B and ^{19}F shifts are referenced relative to external BF_3•Et_2O and hexafluorobenzene, respectively. Coupling constants J are given in Hertz (Hz) as positive values regardless of their real individual signs. Unless otherwise stated NMR spectroscopy was undertaken at room temperature (~20 °C). The multiplicity of the signals are indicated as "s", "d", "t" "q" "pent", "sept" or "m" for singlet, doublet, triplet, quartet, pentet, septet or multiplet, respectively. Mass spectrometry was performed by the University of Edinburgh, School of Chemistry, Mass spectrometry Laboratory using electrospray ionisation.

It should be noted that the very broad signals observed at ca. 0 ppm in the ^{11}B NMR spectra are due to the use of borosilicate glass NMR tubes and boron containing parts in the NMR cavity. These are not coincident with the broad compound signals.

S2. Synthesis of novel NHC-Zinc species

7DippZnEt$_2$

$ZnEt_2$ (0.72 mL, 1 M in hexanes, 0.72 mmol) was dissolved in toluene (5 mL), A solution of 7Dipp (300 mg, 0.720 mmol) in toluene (8 mL) was added dropwise. Volatiles were then removed *in vacuo* resulting in the isolation of the product 7DippZnEt$_2$ as an off-white powder. Yield: (325 mg, 0.600 mmol, 83%). Crystals suitable for X-ray crystallography were grown from a concentrated toluene solution stored at -30°C.

^{1}H NMR (500 MHz, C_6D_6) δ 7.20 – 7.07 (m, 6H), 3.48 (br s, 3.3 Hz, 4H), 3.37 (sept, J = 6.9 Hz, 4H), 1.71 (br s, 4H), 1.50 (d, J = 6.8 Hz, 12H), 1.30 (t, J = 8.0 Hz, 6H), 1.13 (d, J = 6.9 Hz, 12H), -0.31 (q, J = 8.1 Hz, 4H).

^{13}C{^{1}H} NMR (126 MHz, C_6D_6) δ 145.15, 144.47, 128.92, 125.10, 55.84, 28.78, 26.56, 25.59, 23.76, 14.24, 5.85. (N<u>C</u>N not detected)

CHN analysis Calculated for $C_{33}H_{52}N_2Zn$: C 73.11%, H 9.67%, N 5.17%. Observed: C 72.73%, H 9.66%, N 5.20%.

[7DippZnEt][B(C$_6$F$_5$)$_4$](**4**)

Two separate Schlenk flasks were charged with 7DippZnEt$_2$ (300 mg, 0.550 mmol, 1eq.) and $[Ph_3C][B(C_6F_5)_4]$ (507 mg, 0.550 mmol, eq.) and dissolved in PhCl (8.0mL and 3.0mL respectively). Dropwise addition of the bright yellow $[Ph_3C][B(C_6F_5)_4]$ solution to the solution of 7DippZnEt$_2$ yielded a clear purple solution. All volatiles were then removed *in vacuo* yielding a viscous oil. Addition of pentane (ca. 10 mL) with vigorous stirring formed a pale purple powder, this was then washed with further pentane (3x5 mL) before filtration to yield the product as an off white powder. Yield: (510 mg, 0.430 mmol, 78%).

^{1}H NMR ^{1}H NMR (400 MHz, C_6D_5Br) δ 7.21 (t, J = 7.8 Hz, 2H), 7.04 (d, J = 8.1 Hz, 4H), 3.63 (br s, 4H), 2.88 (sept, J = 6.8Hz, 4H), 1.95 (br s, 4H), 1.14 (d, J = 6.8 Hz, 12H), 1.06 (d, J = 6.8 Hz, 12H), 0.26 (t, J = 8.2Hz, 3H), -0.37 (q, J = 8.2 Hz, 2H).

^{11}B NMR (128 MHz, PhCl) δ -16.32 (s).

^{19}F NMR (376 MHz, PhCl) δ -131.90 – -132.18 (m), -162.77 (t, J = 20.7 Hz), -166.62 (t, J = 19.6 Hz).

$^{13}C\{^1H\}$ NMR (126 MHz, C_6D_5Br) δ 157.83, 148.68 (dm, J_{CF} = 243.18Hz, C_{Ar}-F), 144.80 (C-Ar), 138.52 (dm, J_{CF} = 233.10Hz, C_{Ar}-F), 136.56 (dm, J_{CF} = 135.62Hz, C_{Ar}-F), 131.39 (C-Ar), 129.81 (C-Ar), 126.63 (C-Ar), 55.31 (NCH_2), 28.74 (CH-^{i}Pr), 25.13 ($NCH_2C\underline{H_2}$), 24.65 (CH_3-^{i}Pr), 24.61 (CH_3-^{i}Pr), 9.18 ($ZnCH_2CH_3$), 2.83 ($ZnCH_2CH_3$).

Mass Spectrometry Calculated [M^+]: 511.30252, Observed [M^+] 511.30333

S3. Catalytic C-H Borylation

S3.1 Catalyst optimisation for heteroarene borylation

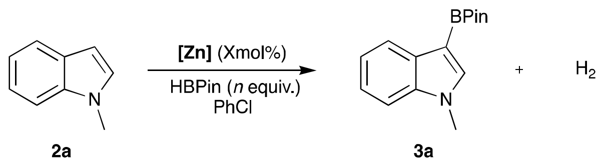

General procedure A: A J Young's NMR tube was charged with a [Zn] complex (X mol%) and dissolved in the selected solvent (500 μL, PhCl unless otherwise stated), HBPin (*n* eq.) was then added followed by N-Me-Indole (**2a**) (63 μL, 0.50 mmol, 1eq.), the reaction mixture was then heated to the desired temperature and monitored periodically by ^{1}H and ^{11}B NMR spectroscopy. Conversions were estimated by comparison of the integrals of the distinguishable N-C$\underline{H}_3$ resonances in **2a** and **3a**. For a number of samples *in-situ* yields were determined by integration of diagnostic ^{1}H resonances of the product against dibromomethane (35μL, 0.50 mmol) added at the end of the reaction as an internal standard.

Table S1 - Optimization study on the catalytic borylation of **2a**.

Experiment	"[**Zn**]"	"[**Zn**]" loading (Xmol%)	HBPin (*n* equiv.)	Temp / °C	Cumulative time / h	Conversion
1	**1-H**	100	2	100	18	100%
2	**1-Ph**	10	2.3	100	18<br>36<br>100	35%<br>78%<br>85%
3[a]	**1-Ph**	10	2.3	100	18<br>36<br>100	52%<br>72%<br>74%
4	**1-Ph**	10	3	100	40<br>64<br>84	50%<br>67%<br>75%
5	**1-Ph**	10	1.5	100	16<br>80	32%<br>72%
6	**1-Ph**	10	1.5	120	16<br>80	57%<br>59%
7	**4**	10	2	80	16<br>36<br>60	36%<br>47%<br>56%
8	**5**	10	2	60	1<br>2<br>16<br>40	26%<br>33%<br>53%<br>82%
9	**5**	10	2	80	18	97%
10[a]	**5**	10	2	80	18	80%
11[b]	**5**	10	2	80	18	91%
12	**5**	5	2	80	18	94%[c]
13	**5**	5	2	80	18	0%[d]
14	**6**	10	2	80	18	0%
15	$Zn(C_6F_5)_2$	5	2	80	18	0%
16	**9**	5	2	80	18	72%

[a] C_6D_6 used as solvent. [b] Toluene used as solvent. [c] Yield confirmed by use of Dibromomethane as an internal standard. [d] H-9BBN used as boron source.

S3.2 Borylation using pinacolborane

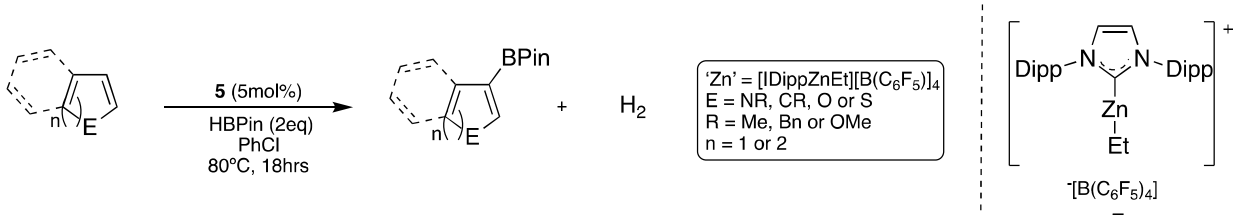

5

General procedure B: A J. Young's NMR tube was charged with **5** (29 mg, 0.025 mmol), PhCl (0.5 mL), Pinacolborane (145 μL, 1.00 mmol) and the substrate (0.50 mmol). The reaction mixture was then heated at 80 ºC for 18 hours and monitored by ^{1}H and ^{11}B NMR spectroscopy. Conversion was estimated by comparison of the integrals of diagnostic resonances in the ^{1}H NMR spectra of the substrate and product. For a number of samples *in-situ* yields were determined by integration of diagnostic ^{1}H resonances against dibromomethane (35μL, 0.50 mmol) added at the end of the reaction as an internal standard.

Table S2 - Scoping of borylation using **5** .

Substrate	Conversion	Substrate	Conversion
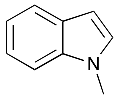	94%[a]	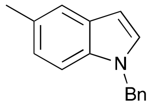	76%[a]
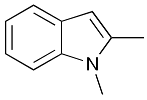	86%[a]	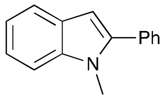	0%
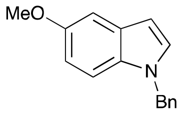	74%[a]	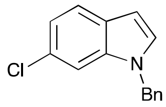	0%
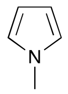	5%[a]	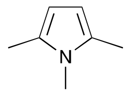	0%
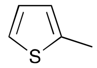	0%	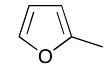	0%
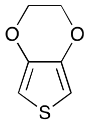	14%	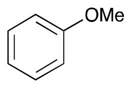	0%

[a]: Confirmed by integration against dibromomethane (35 μL, 0.50mmol).

Borylation of N-Me-indole

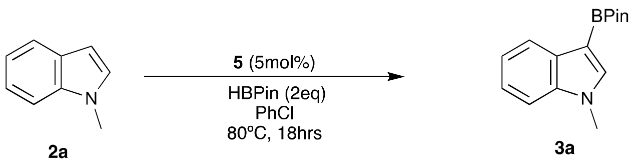

Yield: 94% by integration against dibromomethane as internal standard.

Product was synthesised according to general procedure B, the reaction mixture was then extracted with pentane (ca. 5 mL) and filtered through a short pad of silica rinsing with pentane (ca. 10 mL), the resulting filtrate was dried *in vacuo* and redissolved in $CDCl_3$ allowing confirmation of the product as 1-methyl-3-(4,4,5,5-tetramethyl-1,3,2-dioxaborolan-2-yl)-indole by comparison of NMR data to previously reported literature.[11]

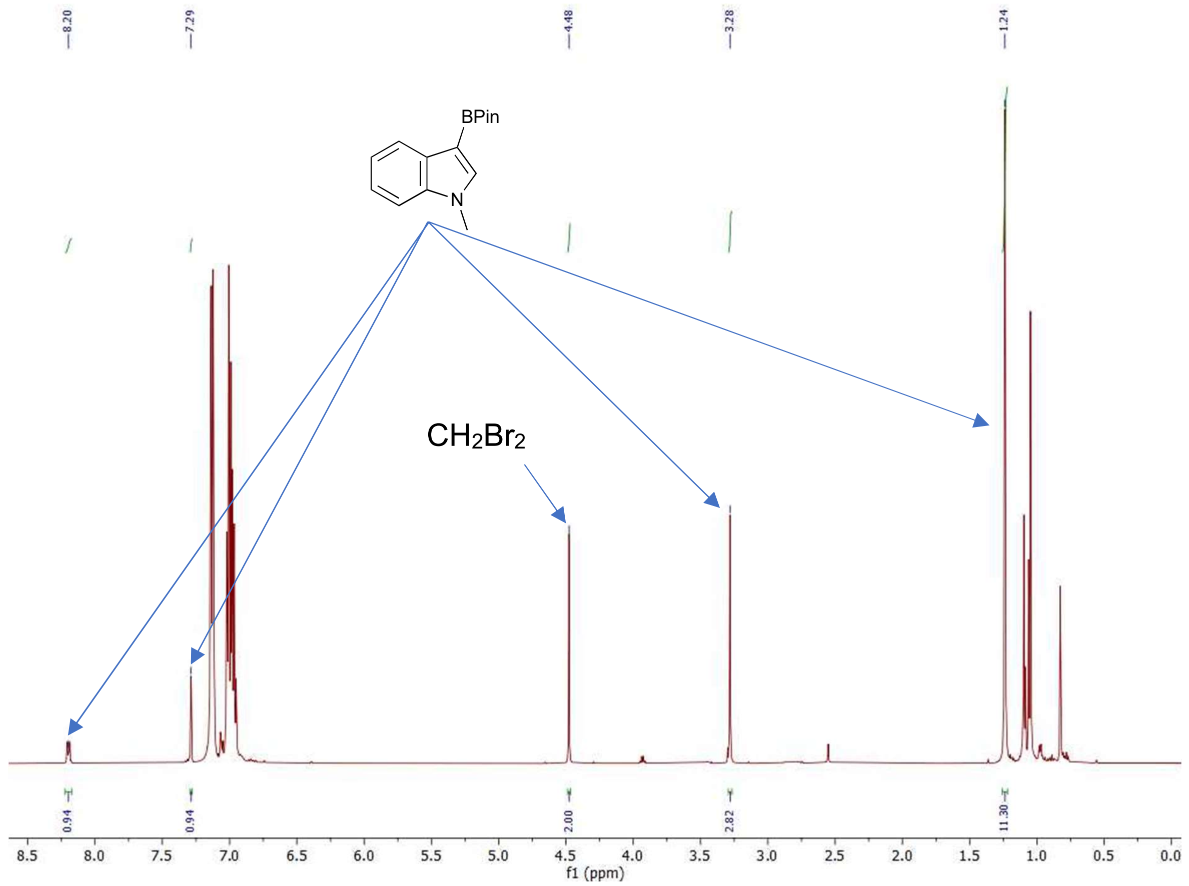

Figure S1: Borylation of N-Me-indole using **5** in PhCl with CH_2Br_2 internal standard as observed by ^{1}H NMR spectroscopy of the crude reaction mixture.

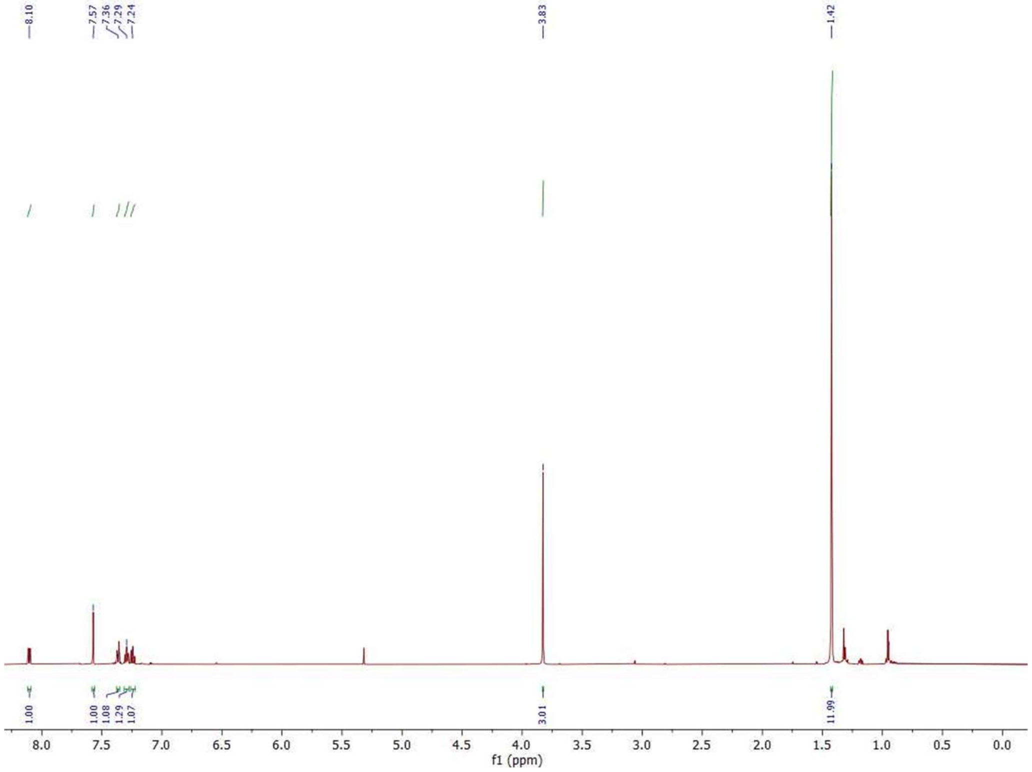

Figure S2: ^{1}H NMR spectrum ($CDCl_3$) of reaction mixture after partial work-up/ filtration .

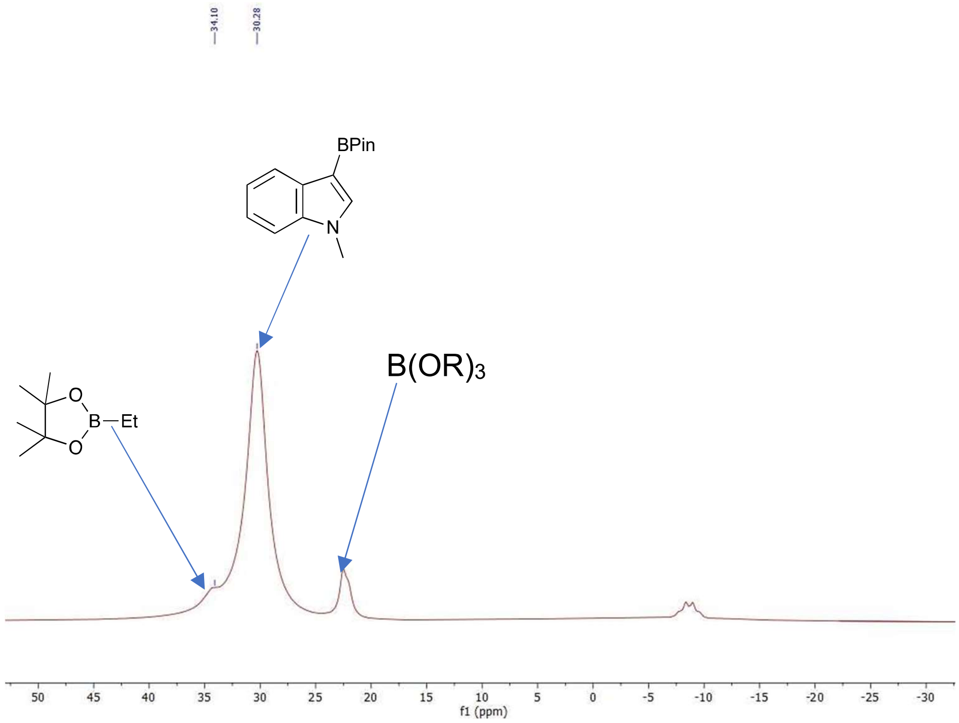

Figure S3: ^{11}B NMR spectrum ($CDCl_3$) of reaction mixture after partial work-up/ filtration

Borylation of 5-Methyl-N-benzyl-indole

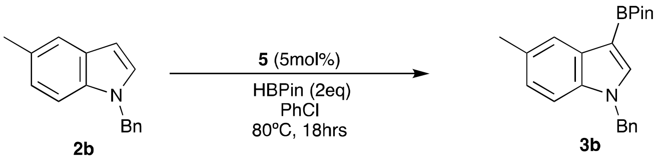

Yield: 76% by integration against dibromomethane as internal standard.

Product was synthesised according to general procedure B, the reaction mixture was then extracted with pentane (ca. 5 mL) before filtration through a short pad of silica (silica pretreated with NEt_3 (ca. 5mL)) using pentane (ca. 10 mL) as an eluent. The resulting filtrate was dried *in vacuo* and redissolved in $CDCl_3$ allowing confirmation of the product as 5-methyl-N-benzyl-3-(4,4,5,5-tetramethyl-1,3,2-dioxaborolan-2-yl)-indole, unfortunately clean isolation of this compound proved elusive in our hands despite multiple attempts due to its propensity to undergo protodeboronation. ^{1}H, ^{11}B and $^{13}C\{^{1}H\}$ NMR spectra of the crude filtrate can be seen in **Figures S5-7**.

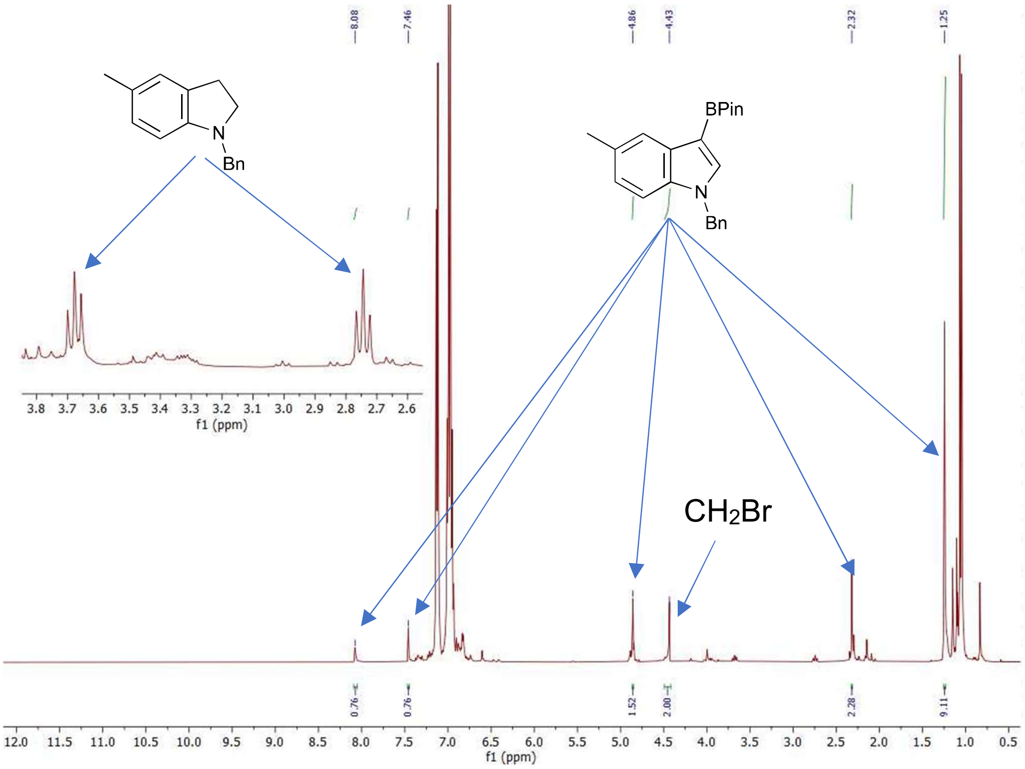

Figure S4: Borylation of 5-Me-N-Bn-indole using **5** in PhCl with CH_2Br_2 internal standard as observed by ^{1}H NMR spectroscopy. Inset left, diagnostic resonances for the indoline congener.

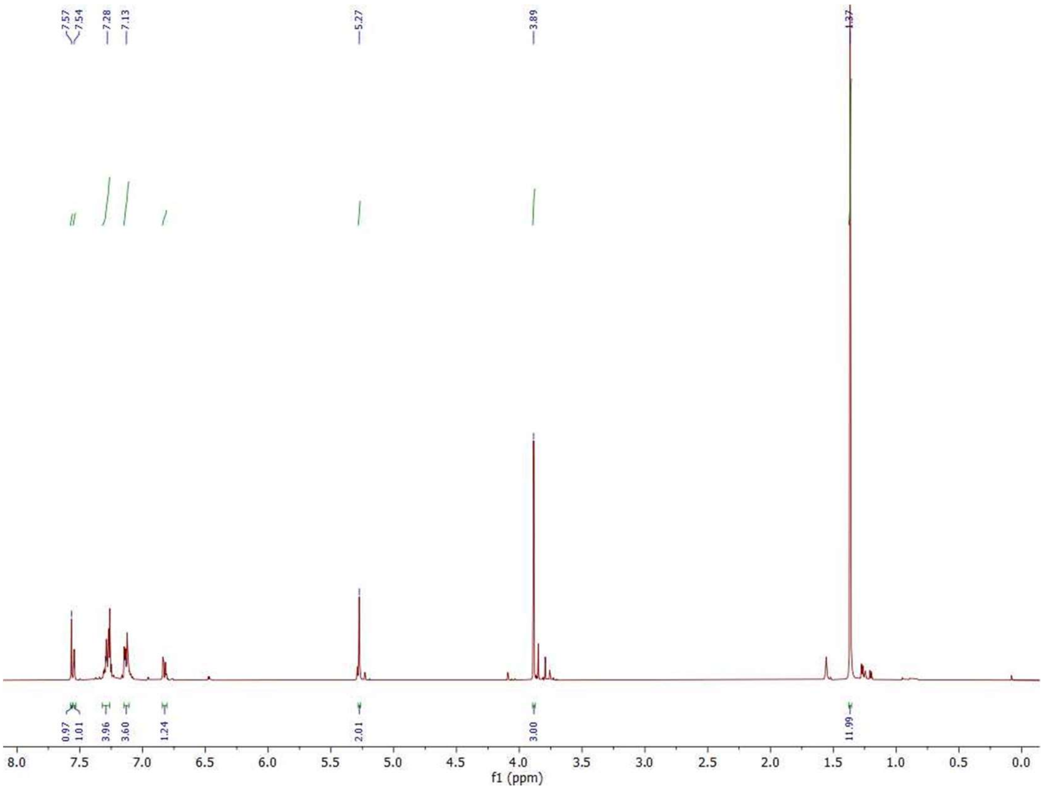

Figure S5: ^{1}H NMR spectrum ($CDCl_3$) of reaction mixture after partial work-up/ filtration

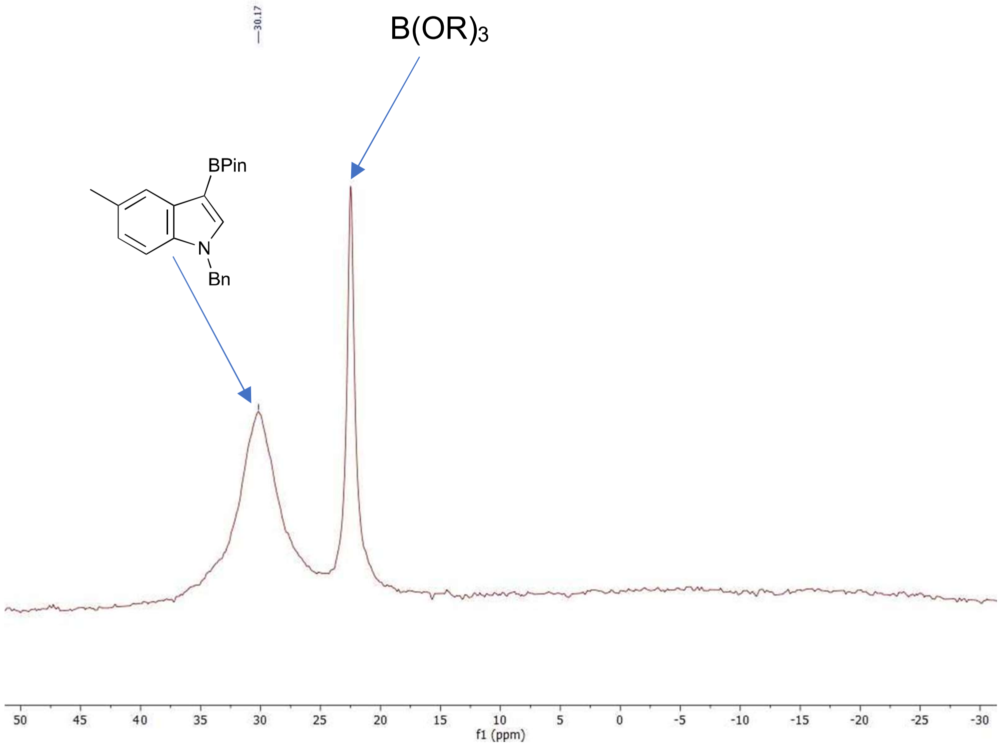

Figure S6: ^{11}B NMR spectrum ($CDCl_3$) of reaction mixture after partial work-up/ filtration

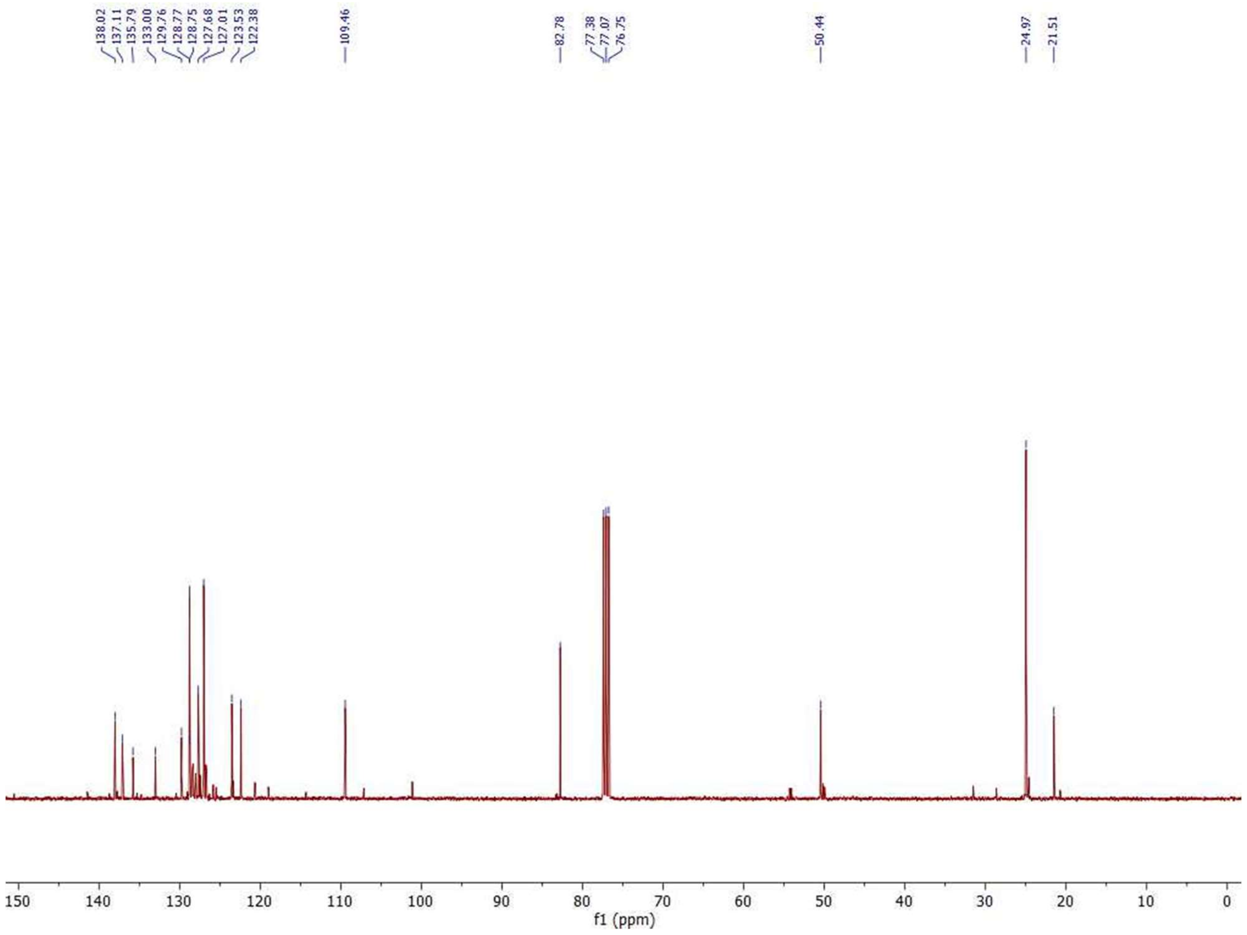

Figure S7: $^{13}C\{^{1}H\}$ NMR spectrum ($CDCl_3$) of reaction mixture after partial work-up/ filtration

Borylation of 5-methoxy-N-benzyl-indole

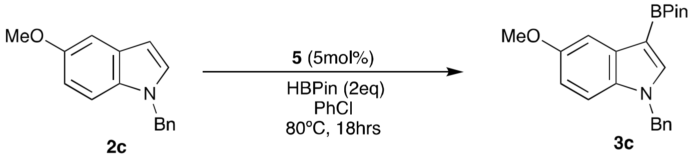

Yield: 74% by integration against dibromomethane as internal standard.

Product was synthesised according to general procedure B, the reaction mixture was then extracted with pentane (ca. 5 mL) before filtration through a short pad of silica (pretreated with NEt_3 (ca. 5mL)) using pentane (ca. 10 mL) as an eluent, the resulting filtrate was dried *in vacuo* and redissolved in $CDCl_3$ allowing confirmation of the product as 5-methoxy-N-benzyl-3-(4,4,5,5-tetramethyl-1,3,2-dioxaborolan-2-yl)-indole, unfortunately clean isolation of this compound proved elusive in our hands despite multiple attempts due to its propensity to undergo protodeboronation. ^{1}H, ^{11}B and $^{13}C\{^{1}H\}$ NMR spectra of the crude filtrate can be seen in **Figures S9-11**.

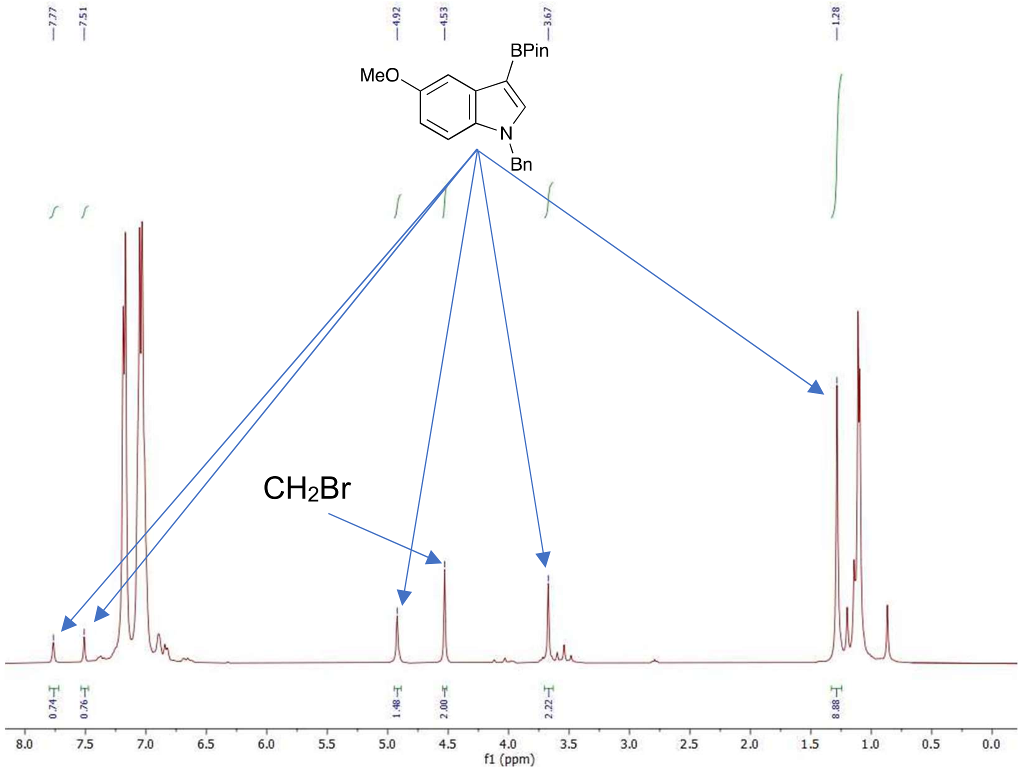

Figure S8: Borylation of 5-MeO-N-Bn-indole using **5** in PhCl with CH_2Br_2 internal standard as observed by ^{1}H NMR spectroscopy.

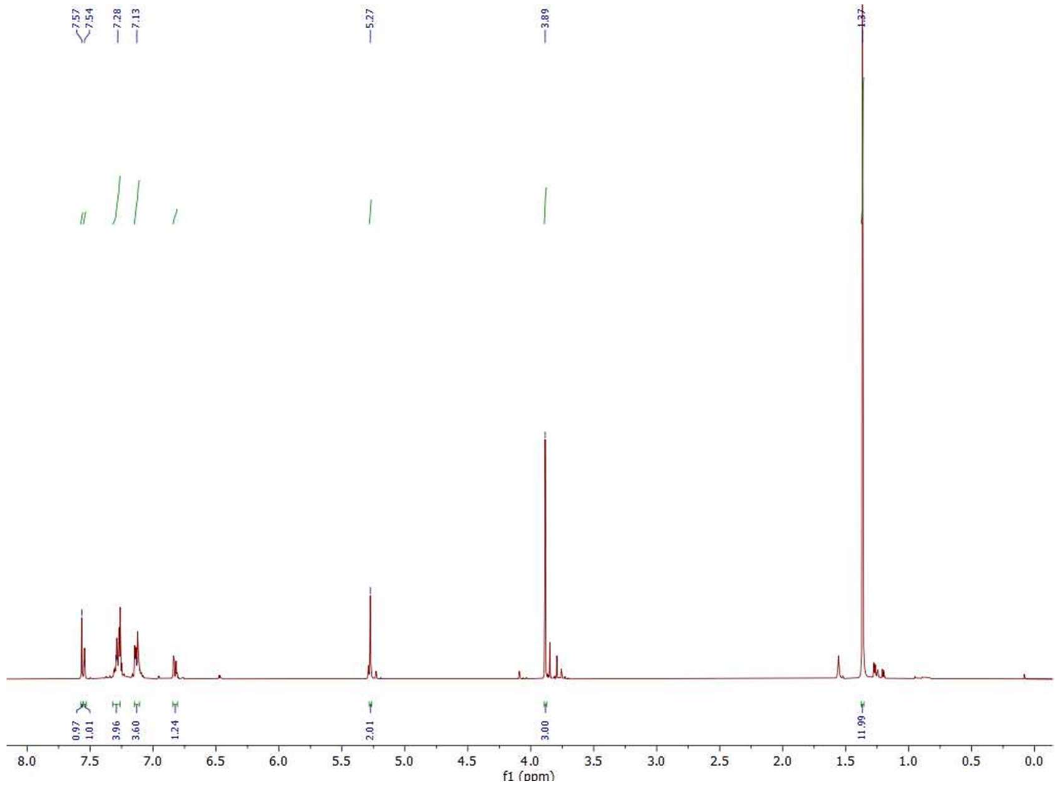

Figure S9: ^{1}H NMR spectrum ($CDCl_3$) of reaction mixture after partial work-up/ filtration

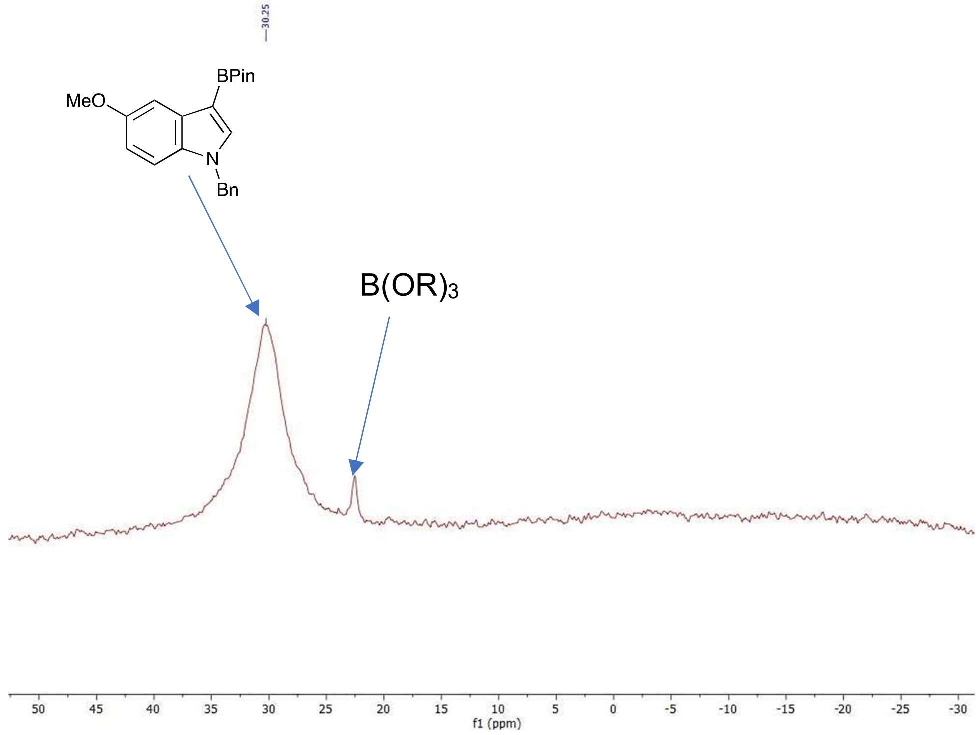

Figure S10: ^{11}B NMR spectrum ($CDCl_3$) of reaction mixture after partial work-up/ filtration.

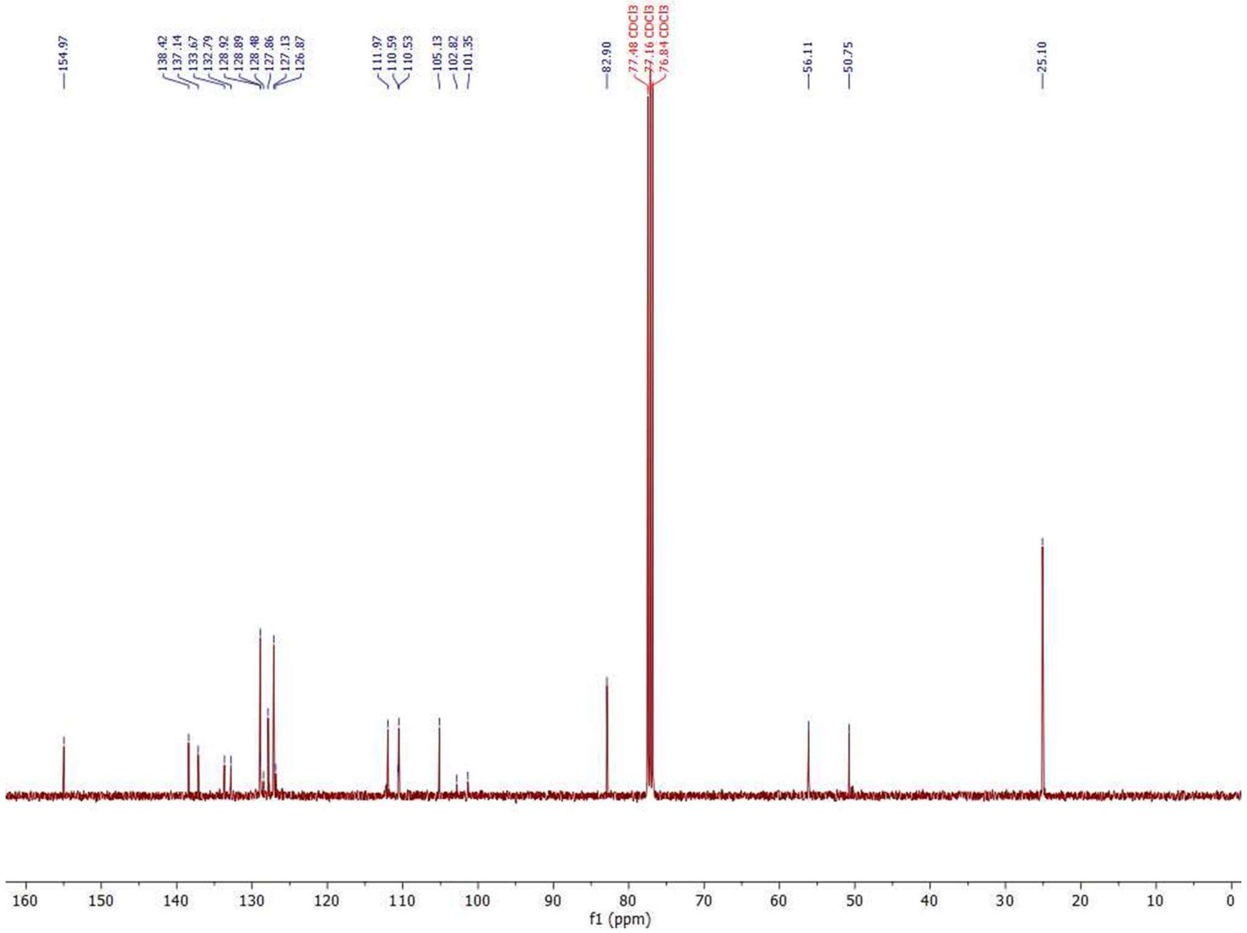

Figure S11: $^{13}C\{^{1}H\}$ NMR spectrum ($CDCl_3$) of reaction mixture after partial work-up/ filtration

Borylation of 1,2-dimethylindole

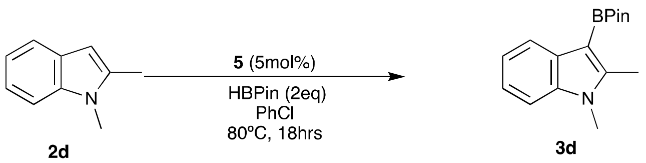

Yield: 86% by integration against dibromomethane as internal standard.

Product was synthesised according to general procedure B, the reaction mixture was then extracted with pentane (ca. 5 mL) and filtered through a short pad of silica rinsing with pentane (ca. 10 mL), the resulting filtrate was dried *in vacuo* and redissolved in $CDCl_3$ allowing confirmation of the product as 1,2-dimethyl-3-(4,4,5,5-tetramethyl-1,3,2-dioxaborolan-2-yl)-indole by comparison of NMR data to previously reported literature.[11]

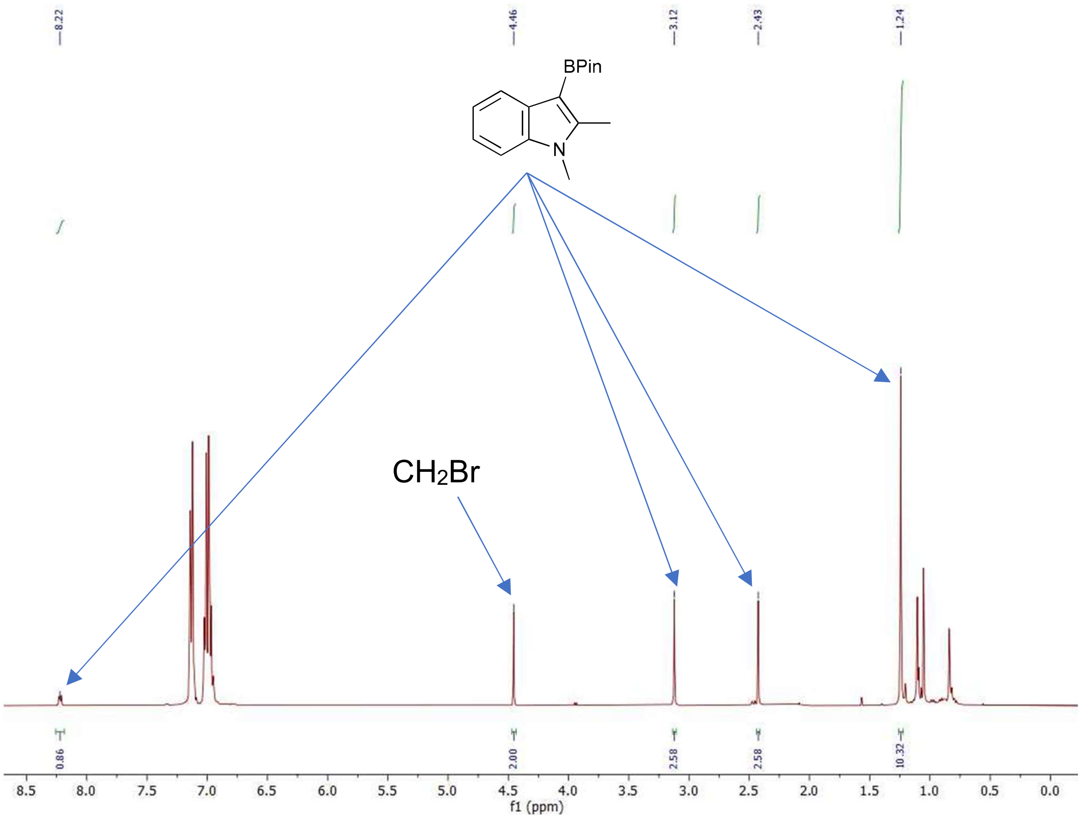

Figure S12: Borylation of 1,2-dimethylindole using **5** in PhCl with CH_2Br_2 internal standard as observed by ^{1}H NMR spectroscopy.

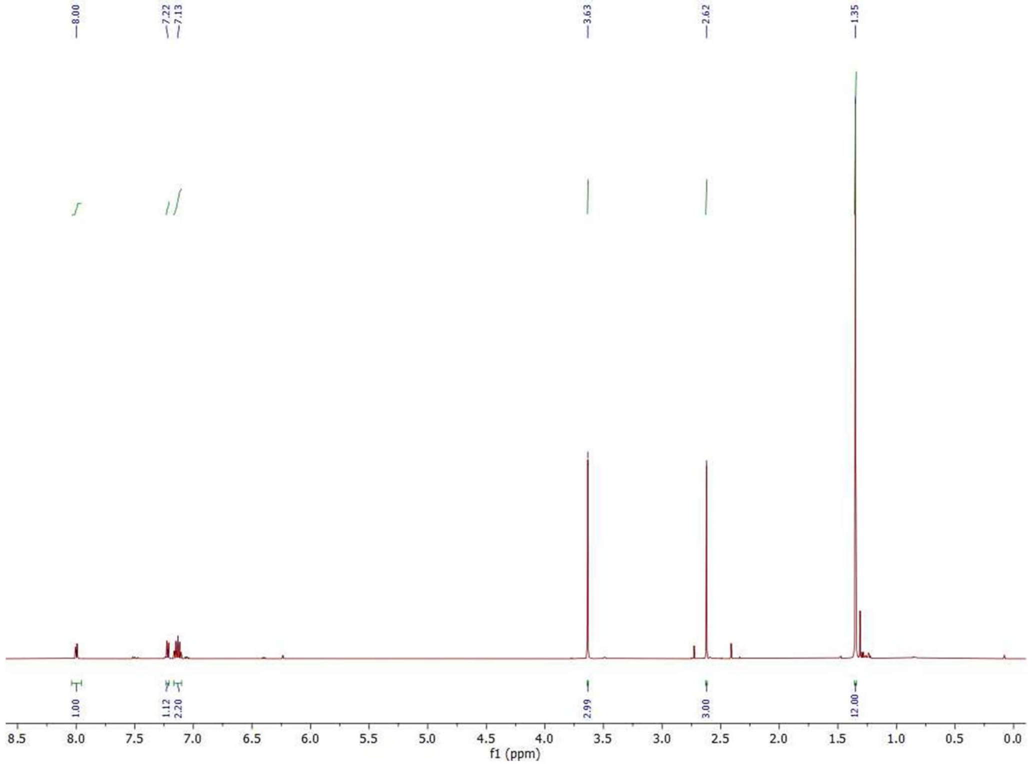

Figure S13: ^{1}H NMR spectrum ($CDCl_3$) of reaction mixture after partial work-up/ filtration

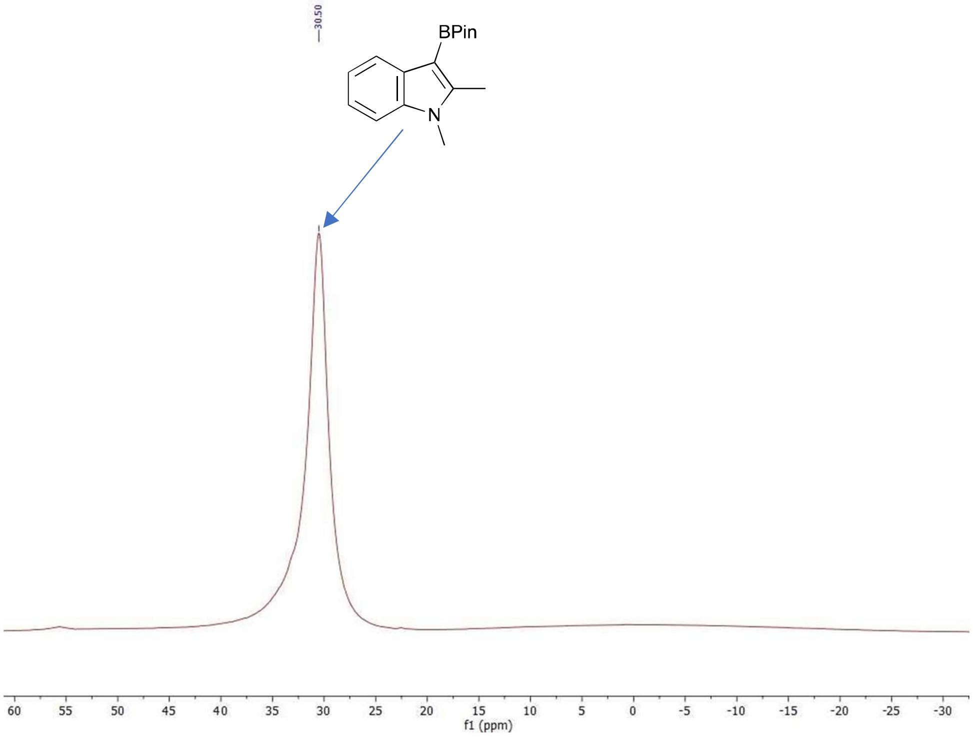

Figure S14: ^{11}B NMR spectrum ($CDCl_3$) of reaction mixture after partial work-up/ filtration

S3.3 Base assisted C-H borylation

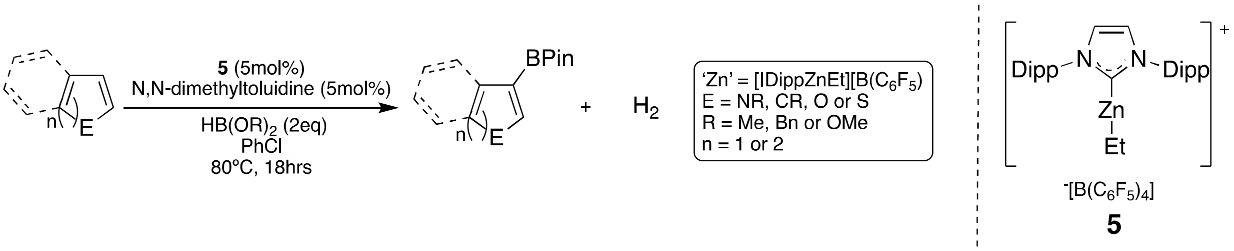

General procedure C: A J. Young's NMR tube was charged with Zinc complex **5** (29 mg, 0.025 mmol), PhCl (0.5 mL), boron source (1.00 mmol, 2 eq.), N,N-dimethyltoluidine (4.0µL, ca. 0.025 mmol) and the substrate (0.50 mmol, 1eq.). The reaction mixture was then heated to the desired temperature and monitored by ^{1}H and ^{11}B NMR spectroscopy. Conversion was estimated by comparison of the integrals of diagnostic resonances in the ^{1}H NMR spectra of the substrate and product. For a number of samples *in-situ* yields were determined by integration of diagnostic ^{1}H resonances against dibromomethane (35 µL, 0.50 mmol) added at the end of the reaction as an internal standard. DBP = 2,6-di-tert-butyl-4-methylpyridine, DMT = N,N-dimethyl-*p*-toluidine.

Table S3 - Summary of base assisted C-H borylation reactions.

Substrate	Catalyst (loading)	Base (loading)	Temp / °C	Time / hrs	Boron source	Conversion
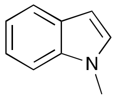	**5** (5 mol%)	DBP (100mol%)	80	18	HBPin	62%[a]
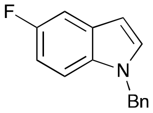	**5** (5 mol%)	DBP (100mol%)	80	1	HBCat	0%
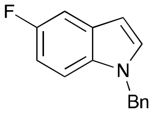	**5** (5 mol%)	-	80	1	HBCat	67%[a]
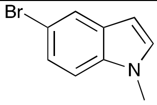	**5** (5 mol%)	DMT (5 mol%)	80	18	HBCat	>99%[a]

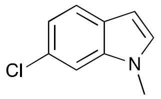	**5** (5 mol%)	DMT (5 mol%)	80	18	HBCat	>99%[a]
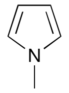	**5** (5mol%)	DMT (5 mol%)	80	18	HBCat	89%[a,b]
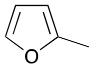	**5** (5mol%)	DMT (5 mol%)	80	36	HBCat	55%[a]
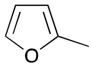	**5** (5mol%)	DMT (5 mol%)	100	18	HBCat	81%[a]
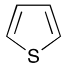	**5** (5mol%)	DMT (5 mol%)	100	36	HBCat	22%[a]
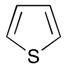	**5** (5mol%)	DMT (5 mol%)	100	120	HBCat	51%[a]
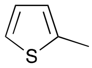	**5** (5 mol%)	DMT (5 mol%)	80	18	HBPin	31%
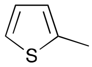	**5** (5 mol%)	DMT (5 mol%)	80	18	HBCat	45%[a]
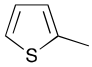	**5** (5 mol%)	DMT (10 mol%)	80	18	HBCat	45%[a]
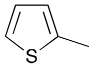	**5** (5 mol%)	DMT (5 mol%)	80	36	HBCat	73%[a]
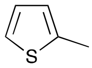	$B(C_6F_5)_3$ (5 mol%)	DMT (5 mol%)	80	36	HBCat	32%[a]
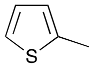	**5** (5 mol%)	DMT (5 mol%)	100	18	HBCat	74%[a]

[a] Yield confirmed by use of dibromomethane as an internal standard. [b] 73:16 mixture of the 2- and 3-BCat regioisomers.

Borylation of 5-Br-N-Me-indole

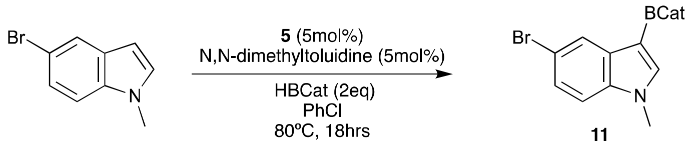

Yield: ca. 99% by integration against dibromomethane as internal standard.

Product was synthesised according to general procedure C, crystals suitable for X-ray crystallography were isolated upon cooling the reaction mixture to room temperature. Analysis of the isolated crystals by X-ray crystallography confirmed the product as 3-(1,3,2-benzodioxaborole)-5-bromo-N-methylindole (**11**). Dissolution of a small amount of unwashed crystalline material in DCM provided additional confirmation of the formulation as **11** by comparison of NMR data to previously reported literature.[12]

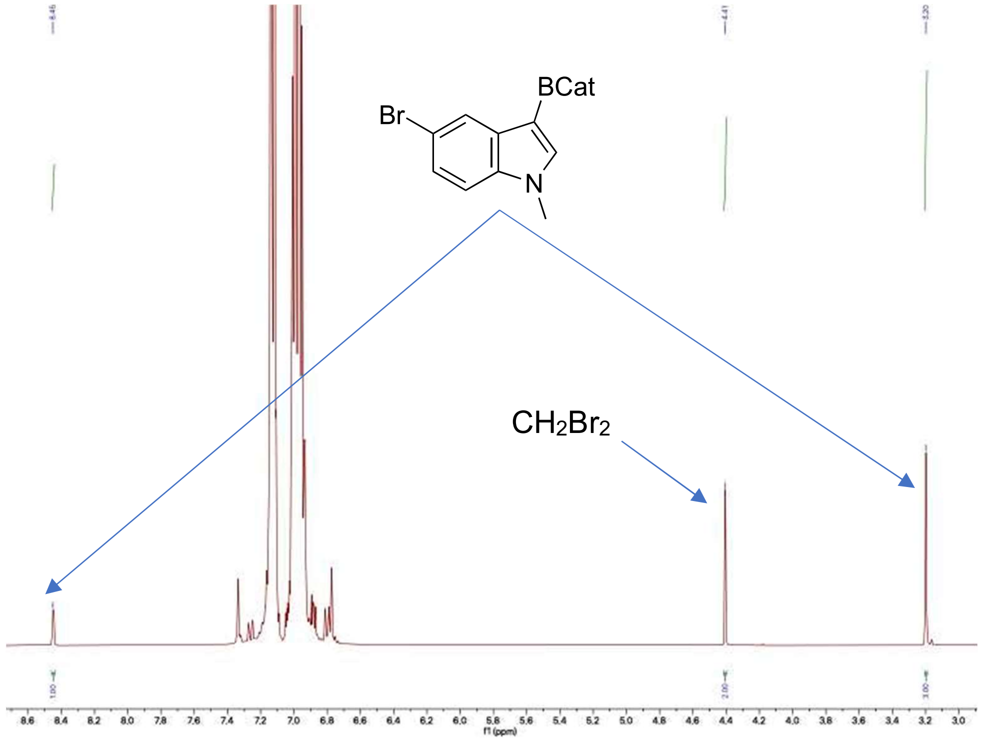

Figure S15: Borylation of 5-Br-N-Me-indole using **5** in PhCl with CH_2Br_2 internal standard as observed by ^1H NMR spectroscopy at 60 °C.

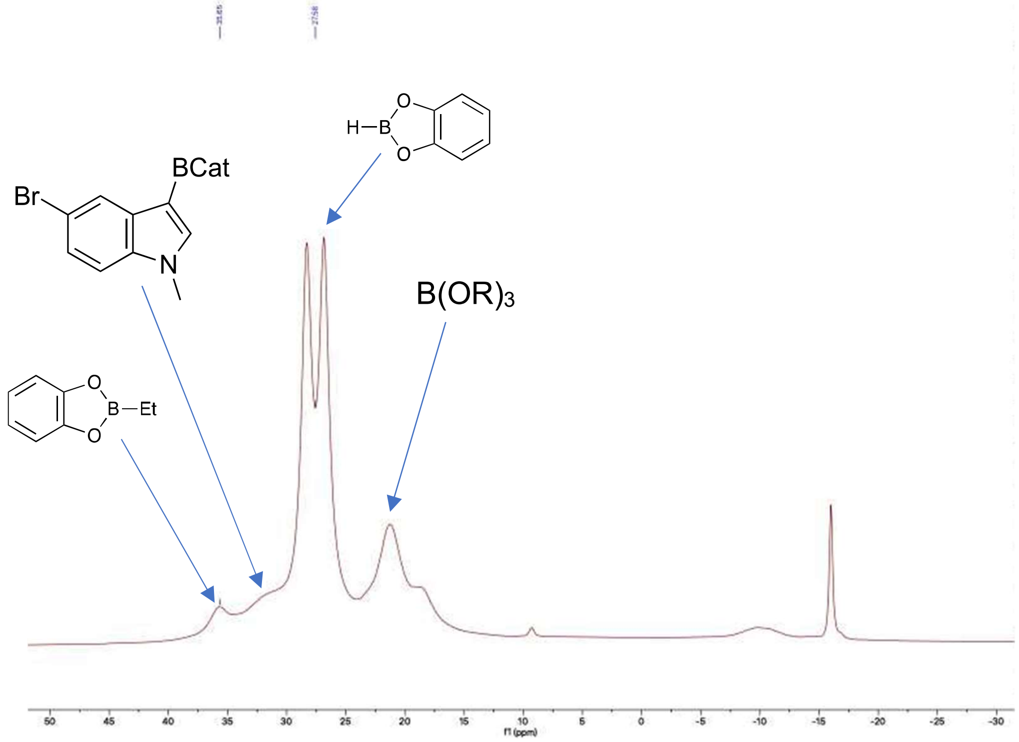

Figure S16: Borylation of 5-Br-N-Me-indole using **5** in PhCl as observed by ^{11}B NMR spectroscopy at 60°C.

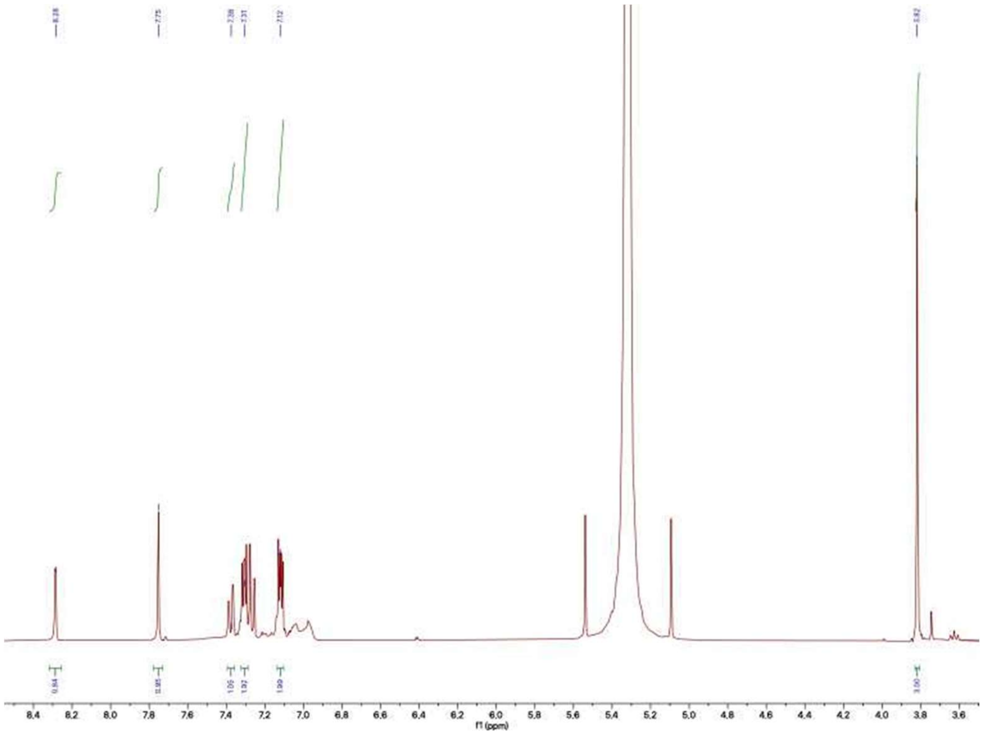

Figure S17: Unwashed crystalline material isolated from borylation of 5-Br-N-Me-indole using **5** as observed by ^{1}H NMR spectroscopy in DCM. Note this compound is poorly soluble in chlorinated organic solvents at room temperature.

Borylation of 6-Cl-N-Me-indole

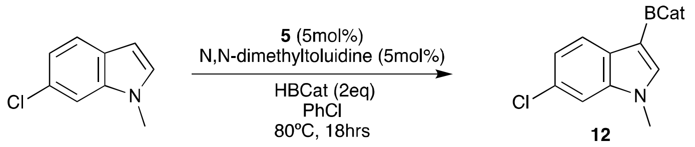

Yield: ca. 99% by integration against dibromomethane as internal standard.

Product was synthesised according to general procedure C but C_6D_5Br (0.5mL) was used as the solvent, volatiles were then removed, and the crude solids were redissolved in C_6D_6 allowing confirmation of the product as 3-(1,3,2-benzodioxaborole)-6-chloro-N-methylindole by comparison of NMR data to previously reported literature.[11]

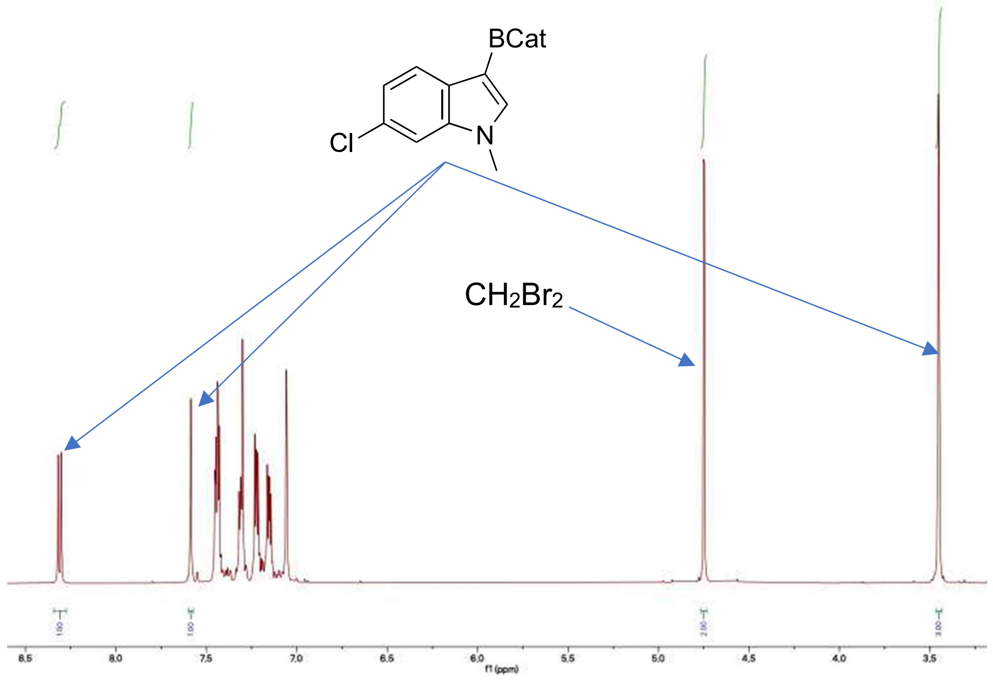

Figure S18: Borylation of 6-Cl-N-Me-indole using **5** in C_6D_5Br with CH_2Br_2 internal standard as observed by ^1H NMR spectroscopy.

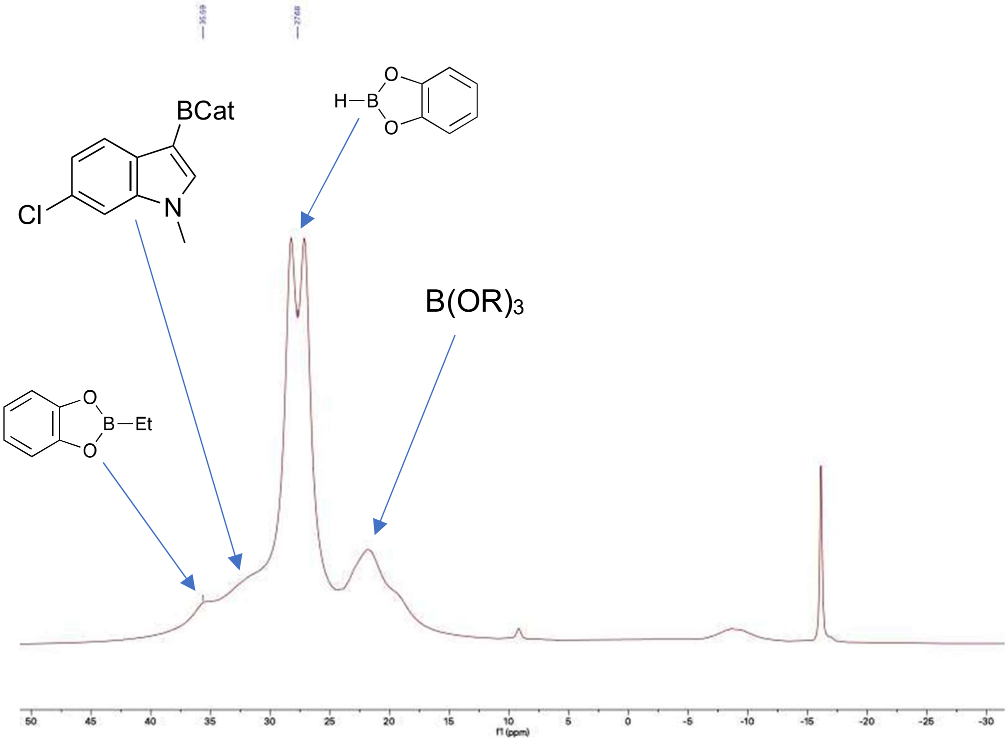

Figure S19: Borylation of 6-Cl-N-Me-indole using **5** in C_6D_5Br as observed by ^{11}B NMR spectroscopy.

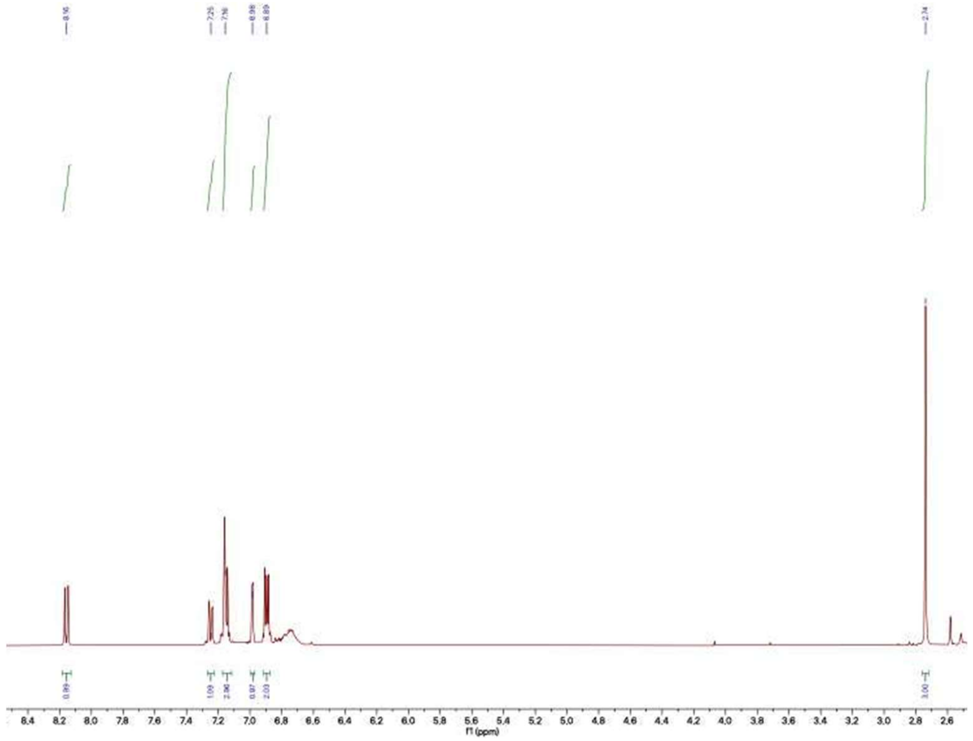

Figure S20: Crude reaction mixture of the Borylation of 6-Cl-N-Me-indole using **5** in as observed by ^{11}B NMR spectroscopy in C_6D_6.

Borylation of N-methylpyrrole

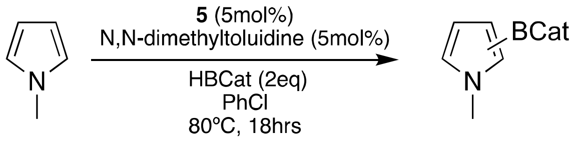

Yield: 89% (73:16 ratio of 2-:3-borylated regioisomers) by integration against dibromomethane as an internal standard.

Product was synthesised according to general procedure C. To confirm the identity conversion to the boronic acid pinacol ester was achieved by drying of the reaction mixture *in vacuo* before dissolving in DCM (0.5 mL) and reacting with 1M solution of pinacol in NEt_3 (0.75 mL, 0.75mmol) for 18 hrs at room temperature. The reaction mixture was then dried before extraction of the product with pentane (ca. 5 mL) and filtering through a short pad of silica and rinsing with pentane (ca. 10 mL). The resulting filtrate was dried *in vacuo* and redissolved in $CDCl_3$ confirming a mixture of regioisomers of 2- and 3-(4,4,5,5-tetramethyl-1,3,2-dioxaborolan-2-yl)-1-methyl-pyrrole, thus confirming initial products are 2-(1,3,2-benzodioxaborole)-N-methylpyrrole and 3-(1,3,2-benzodioxaborole)-N-methylpyrrole

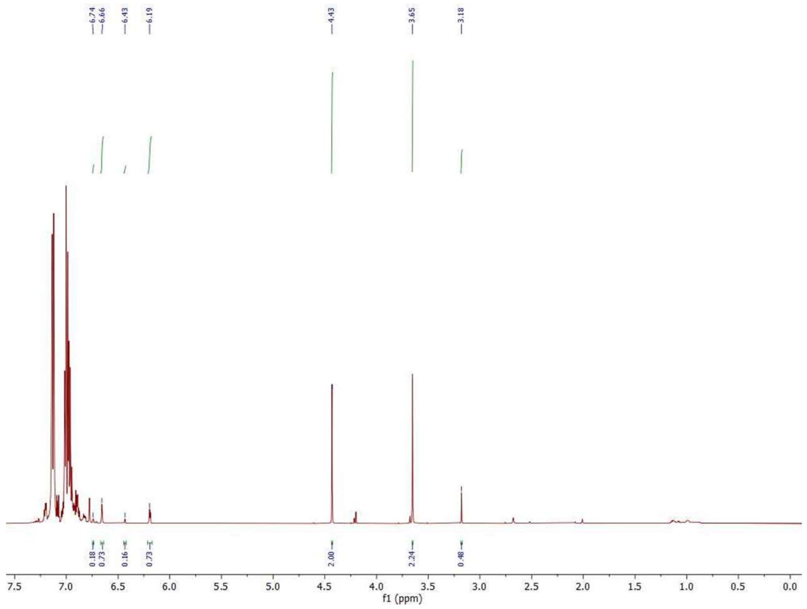

Figure S21: Borylation of N-Me-pyrrole using **5** in PhCl with CH_2Br_2 internal standard as observed by ^1H NMR spectroscopy. Resonance at 4.43 ppm = CH_2Br_2

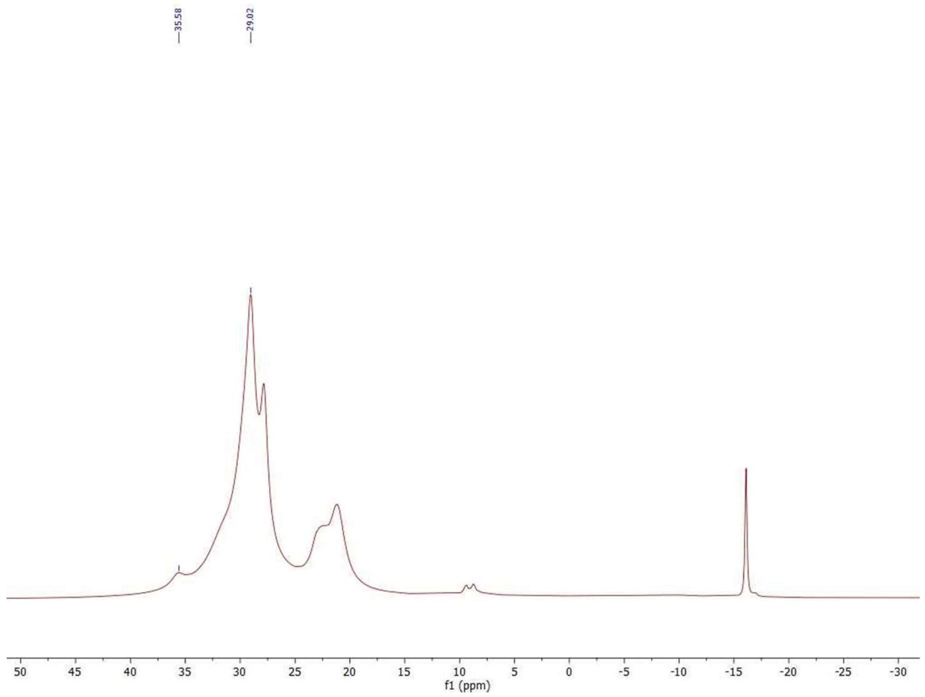

Figure S22: Borylation of N-Me-pyrrole using **5** in PhCl with CH_2Br_2 internal standard as observed by ^{11}B NMR spectroscopy.

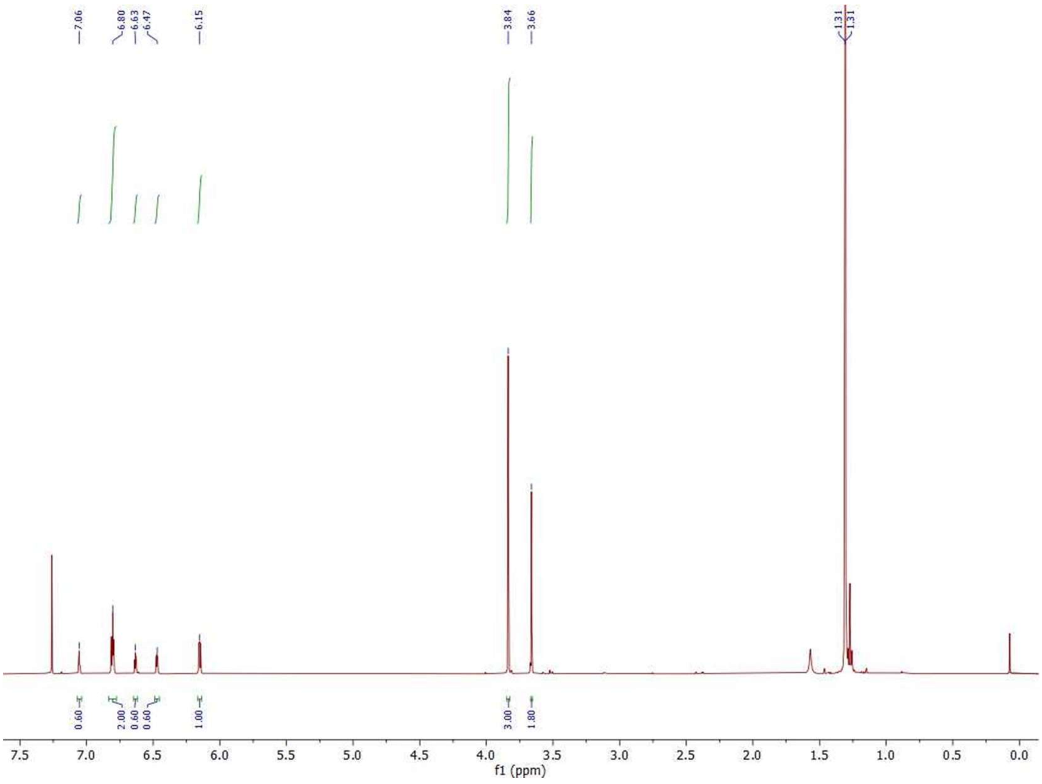

Figure S23: ^1H NMR spectrum ($CDCl_3$) of reaction mixture after pinacol protection/ partial work-up.

Borylation of 2-methylthiophene

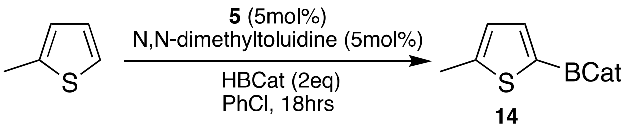

Yield: 45% (at 80 ºC) or 74% (at 100 ºC) by integration against dibromomethane as internal standard.

Product was synthesised according to general procedure C. Conversion to the boronic acid pinacol ester was achieved by drying of the reaction mixture *in vacuo* before dissolving in DCM (0.5 mL) and reacting with 1M solution of pinacol in NEt_3 (0.75 mL, 0.75mmol) for 18hrs at room temperature. The reaction mixture was then dried before extraction of the product with pentane (ca. 5 mL) and filtered through a short pad of silica rinsing with pentane (ca. 10 mL). The resulting filtrate was dried *in vacuo* and redissolved in $CDCl_3$ allowing confirmation of the initial product as 2-methyl-5-(1,3,2-benzodioxaborole)-thiophene by comparison of NMR data to previously reported literature.[13]

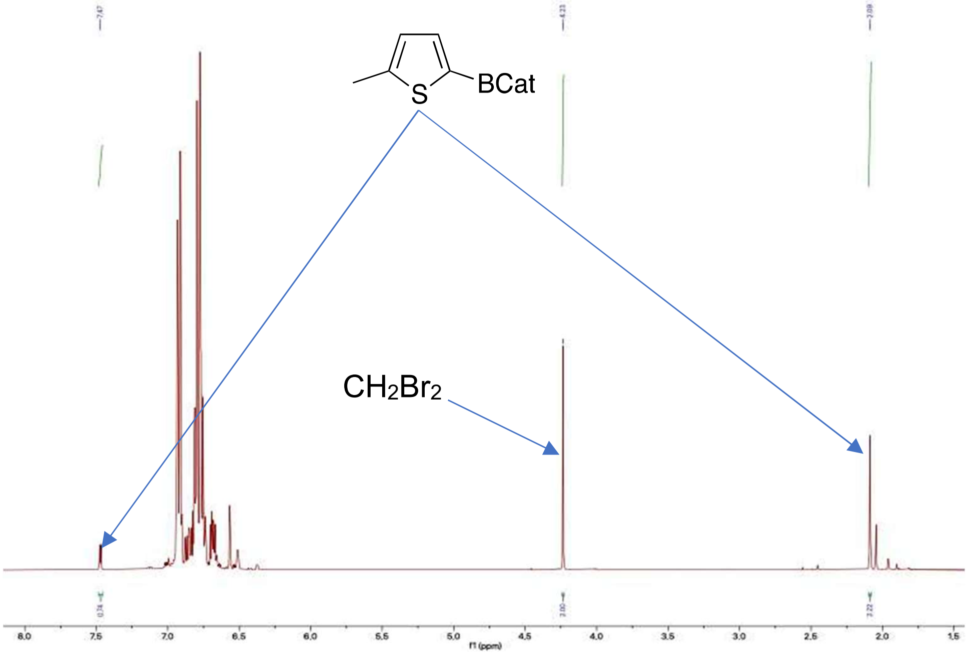

Figure S24: Borylation of 2-methylthiophene using **5** in PhCl with CH_2Br_2 internal standard as observed by ^{1}H NMR spectroscopy.

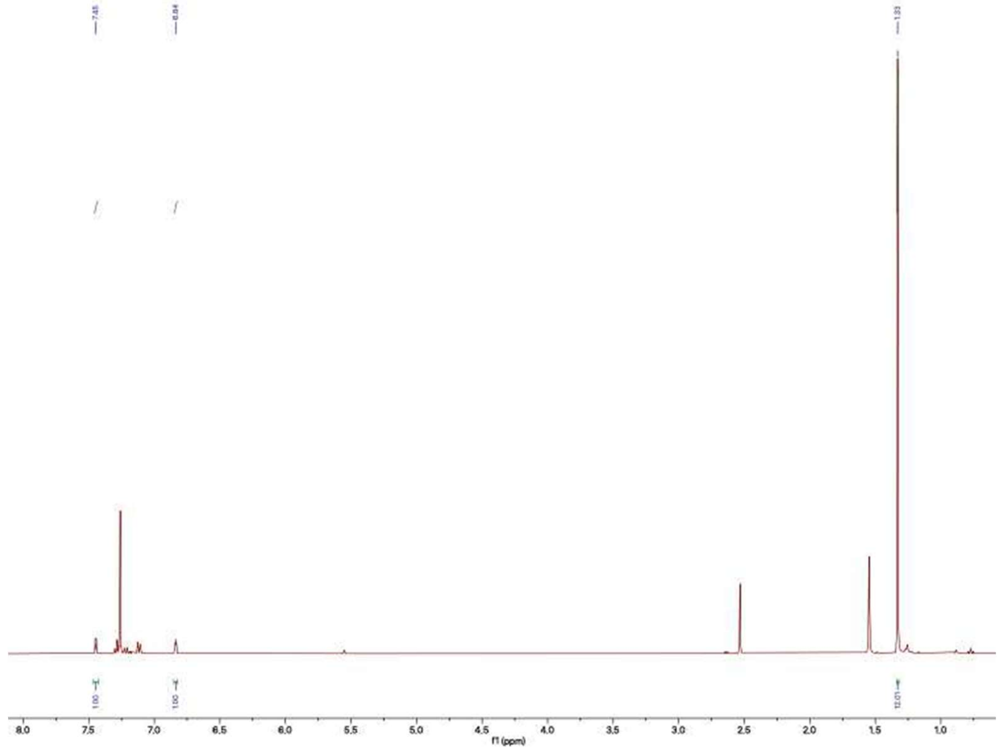

Figure S25: Reaction mixture after filtration post-pinacol esterification as observed by ^{1}H NMR spectroscopy in $CDCl_3$.

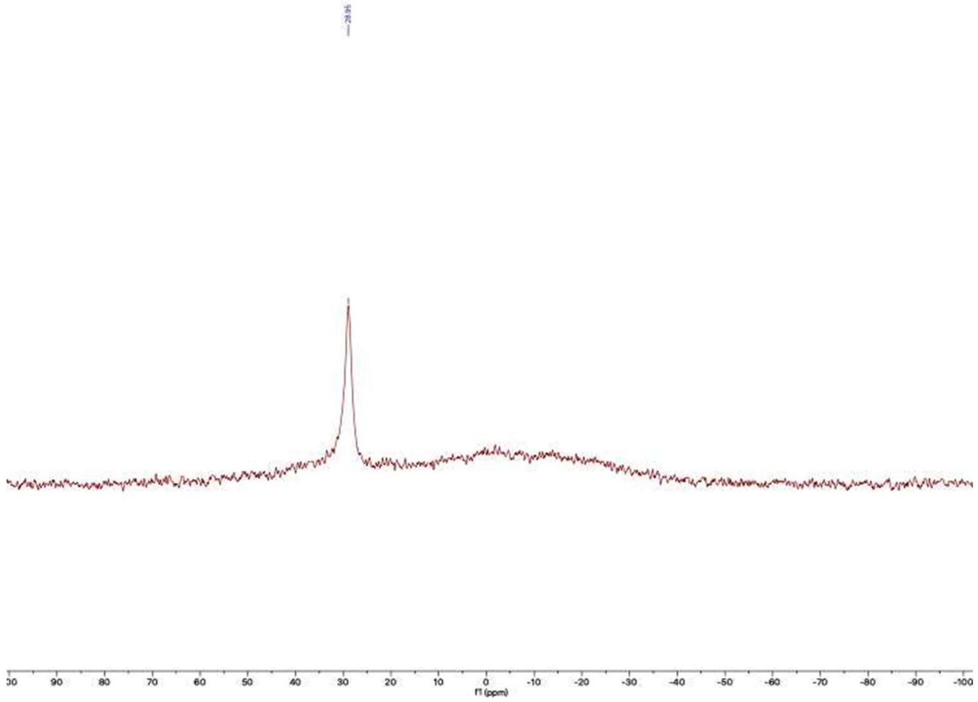

Figure S26: Reaction mixture after filtration post-pinacol esterification as observed by ^{11}B NMR spectroscopy in $CDCl_3$.

N.B. A lower (31%) conversion was achieved after 18 hours at 80 ºC using HBPin as the boron source in place of HBCat (**Figures S27-28**).

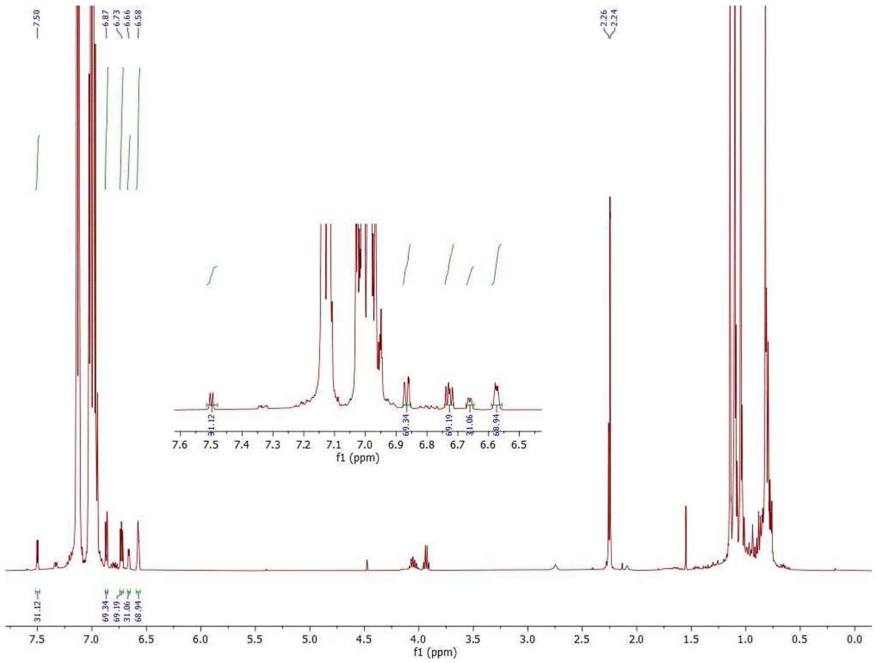

Figure S27: Borylation of 2-Me-thiophene using **5** and HBPin as observed by ^{1}H NMR spectroscopy.

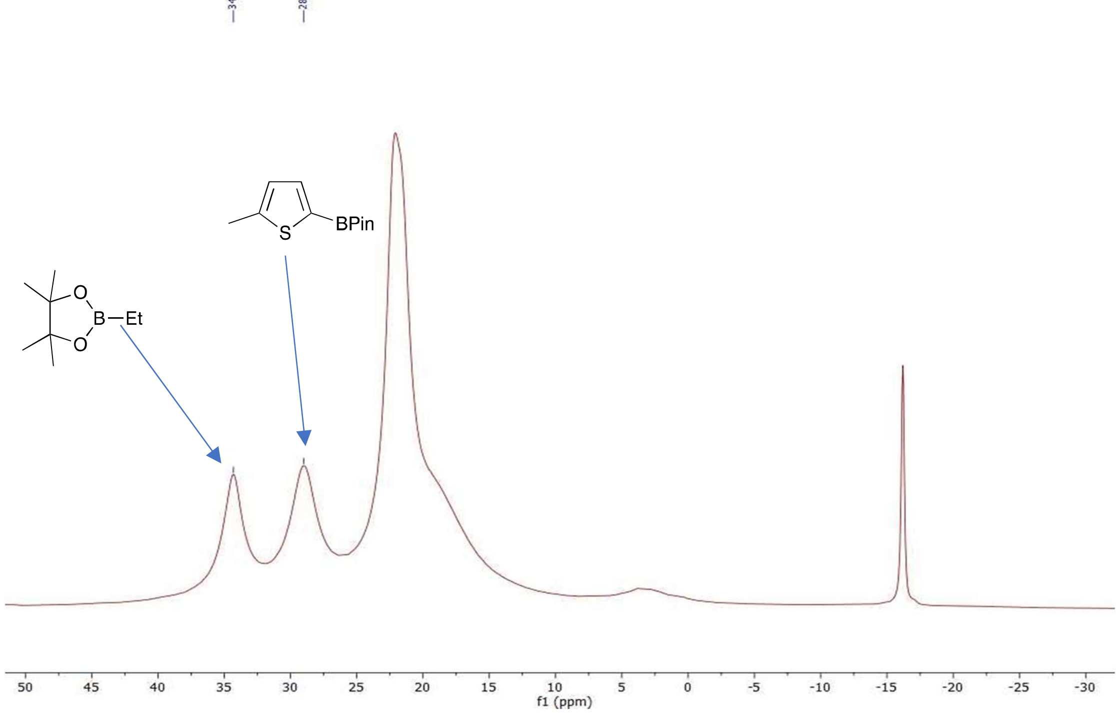

Figure S28: Borylation of 2-Me-thiophene using **5** and HBPin as observed by ^{11}B NMR spectroscopy.

Borylation of 2-methylfuran

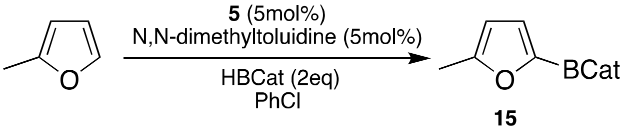

Yield: 55% (36hrs at 80ºC) or 81% (18hrs at 100ºC) by integration against dibromomethane as internal standard.

Product was synthesised according to general procedure C, volatiles were then removed and the crude solids were redissolved in CD_2Cl_2 allowing confirmation of the product as 2-methyl-5-(1,3,2-benzodioxaborole)-furan by comparison of NMR data to previously reported literature.[14]

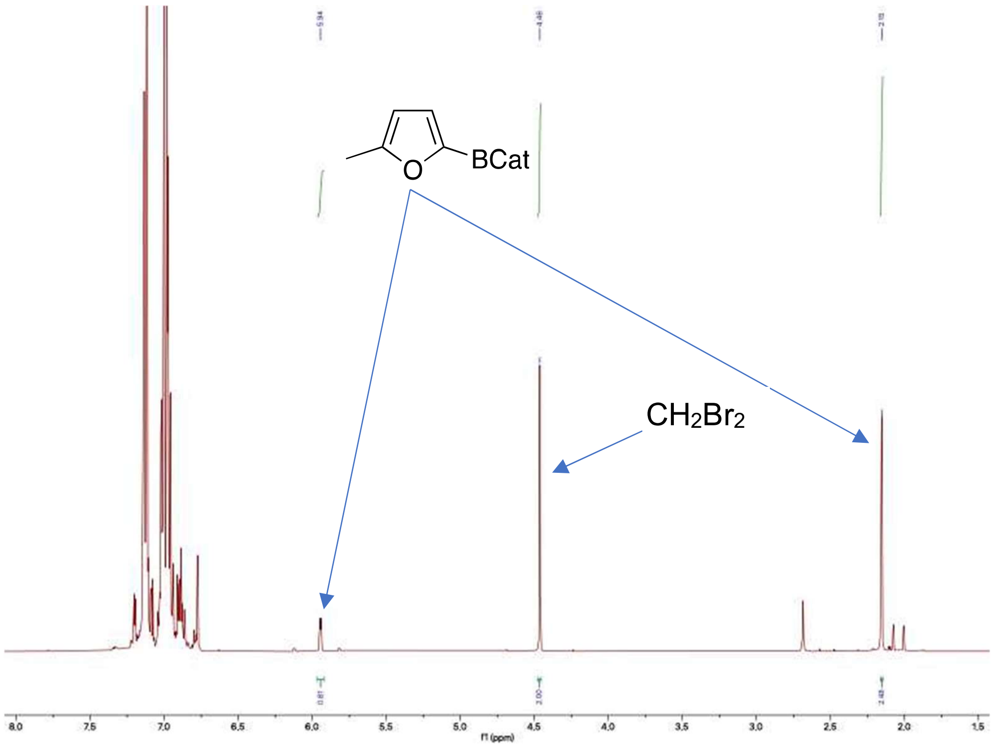

Figure S29: Borylation of 2-methylthiophene using **5** in PhCl with CH_2Br_2 internal standard as observed by ^{1}H NMR spectroscopy.

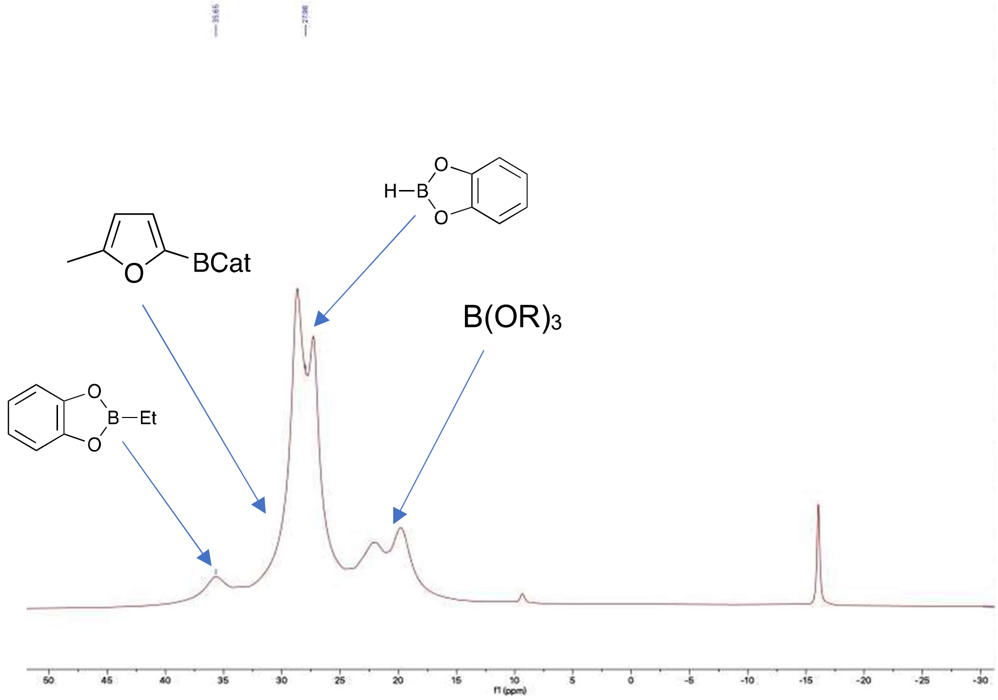

Figure S30: Borylation of 2-methylfuran using **5** in PhCl as observed by ^{11}B NMR spectroscopy.

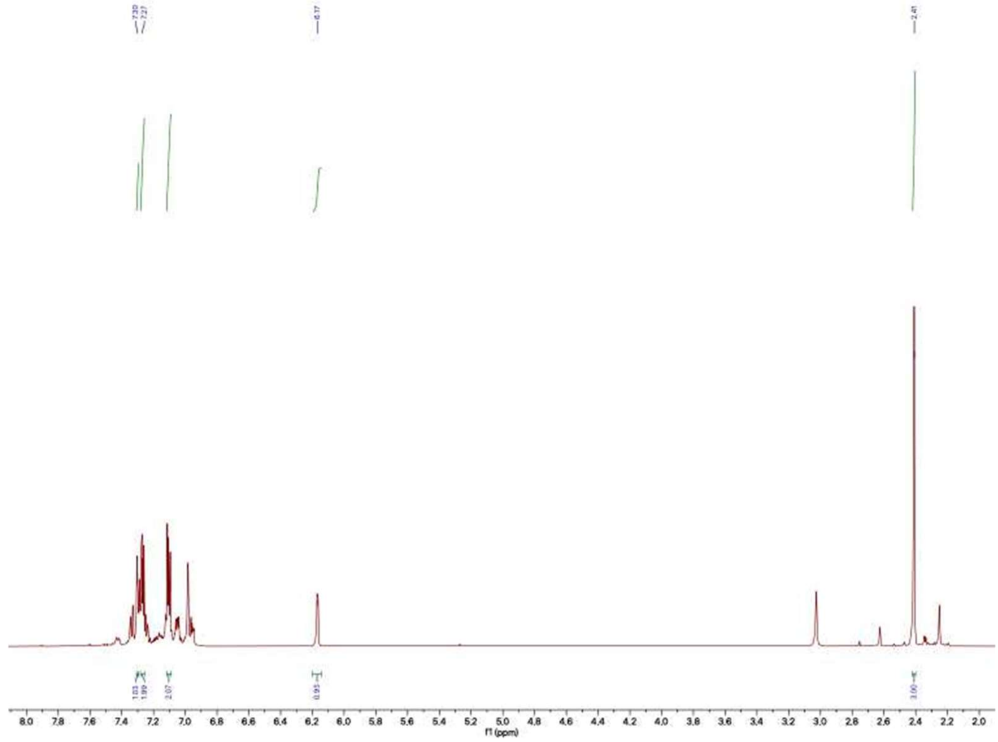

Figure S31: Crude reaction mixture of the borylation of 2-methylfuran using **5** in as observed by ^{11}H NMR spectroscopy in $CDCl_3$.

Borylation of thiophene

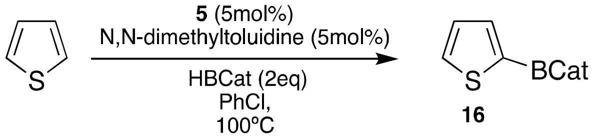

Yield: 22% (36hrs) or 51% (120hrs) confirmed by integration against dibromomethane as internal standard.

Product was synthesised according to general procedure C, before conversion to the boronic pinacol ester by drying of the reaction mixture *in vacuo* before solvation in DCM (0.5 mL) and reaction with 1M solution of pinacol in NEt_3 (0.75 mL) for 18hrs at room temperature. The reaction mixture was then dried and product was confirmed as 2-(4,4,5,5-tetramethyl-1,3,2-dioxaborolan-2-yl)-thiophene by comparison of NMR data to previously reported literature.[15]

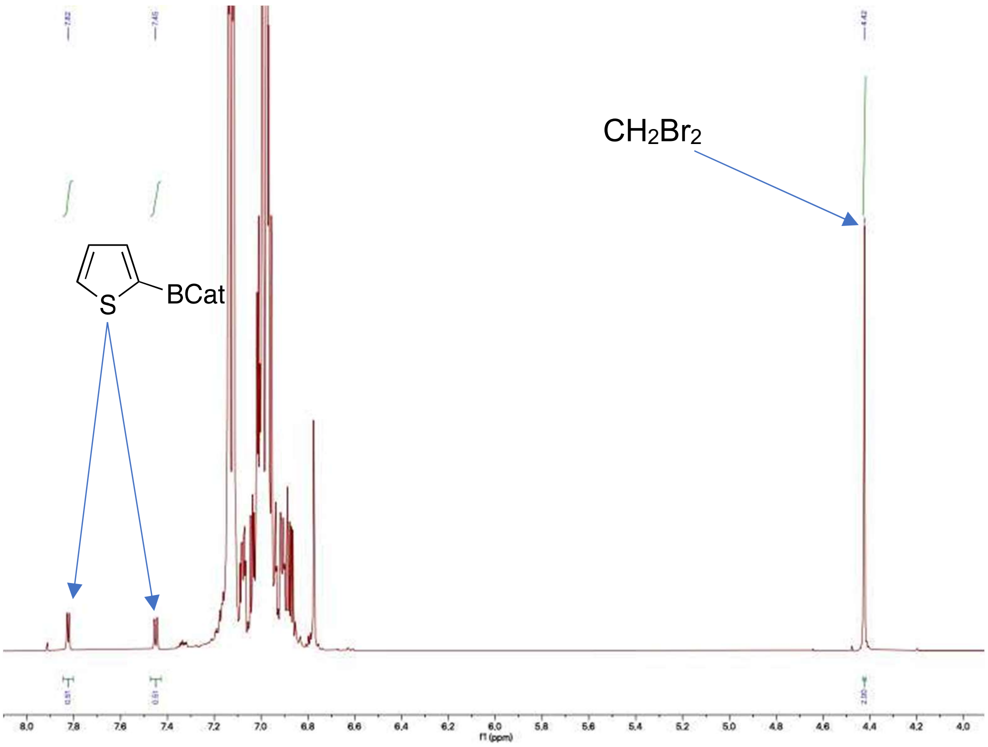

Figure S32: Borylation of thiophene using **5** in PhCl with CH_2Br_2 internal standard as observed by ^1H NMR spectroscopy.

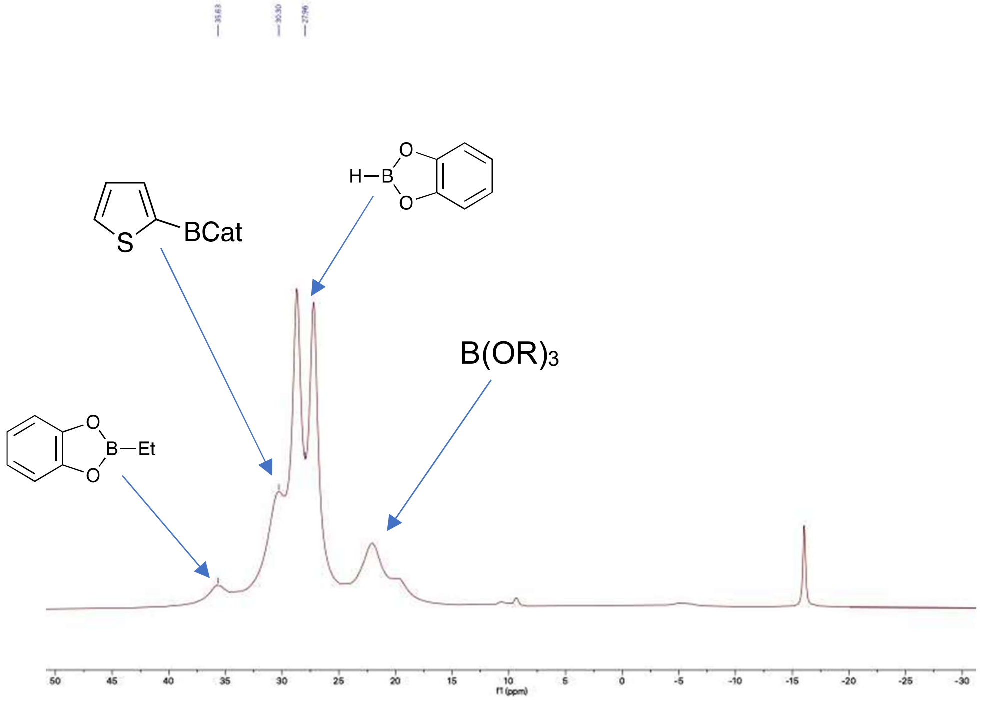

Figure S33: Borylation of thiophene using **5** in PhCl as observed by ^{11}B NMR spectroscopy.

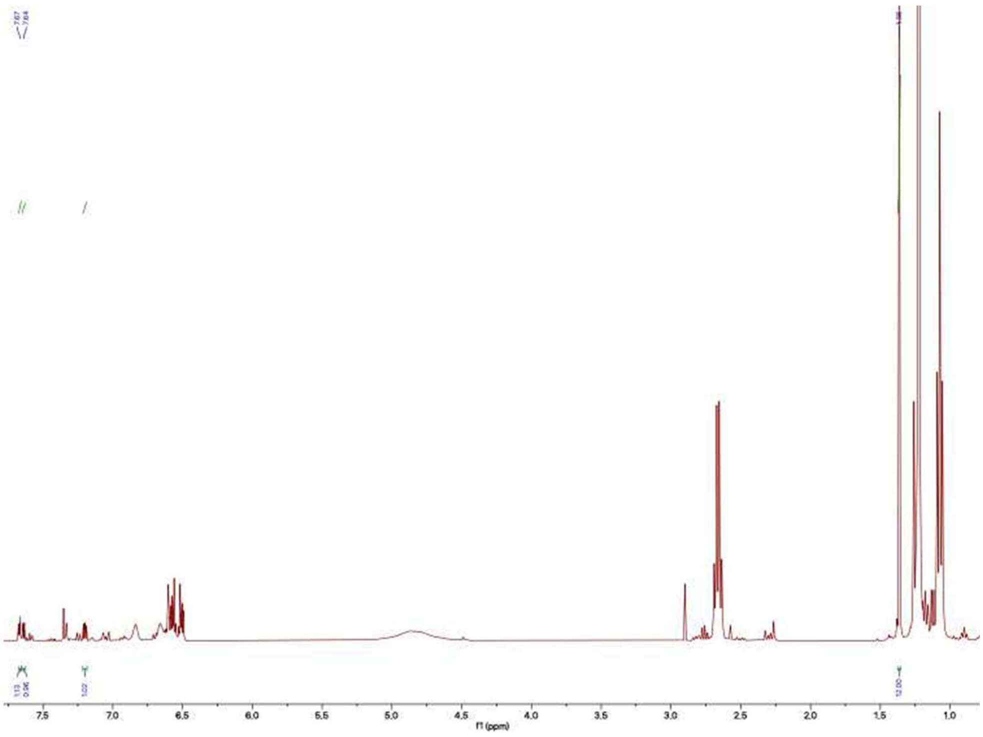

Figure S34: Crude reaction mixture post-pinacol esterification as observed by ^{1}H NMR spectroscopy in $CDCl_3$.

S4. Investigation of potential "Trojan horse" catalysts

S4.1 [IDippBPin][B(C_6F_5)$_4$] (8) as a catalyst

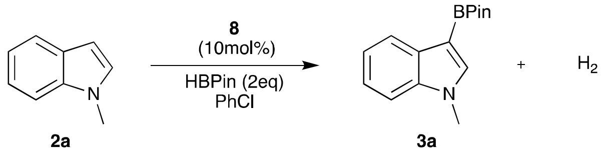

Synthesis of **8** *in-situ*:

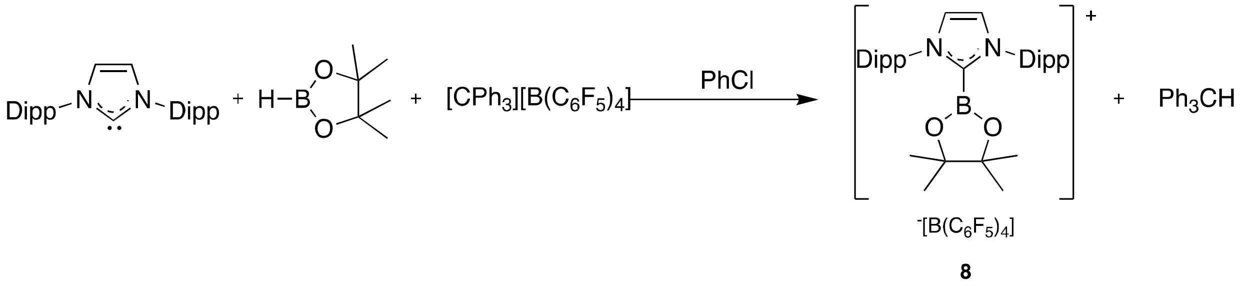

A J Young's NMR tube was charged with IDipp carbene (26 mg, 67 μmmol), the solid was then dissolved in PhCl (500 μL) before addition of HBPin (10 μL, 67 μmmol), analysis by ^{1}H and ^{11}B NMR showed clean production of an adduct (**Figures S35-36**), consistent with previous work.[16] In a separate J Youngs ampoule [CPh_3][B(C_6F_5)$_4$] (62 mg, 67 μmmol) was dissolved in PhCl (ca. 200 μL) before addition to the reaction mixture, analysis by ^{1}H and ^{11}B NMR spectroscopy showed production of the NHC-borenium species.[17] The catalytic reaction was then conducted by addition of HBPin (195 μL, 1.34 mmol) and **2a** (84 μL, 0.67 mmol) before heating at 80 ºC, analysis of the reaction mixture after 18 hours by ^{1}H and ^{11}B NMR spectroscopy showed no conversion of substrate.

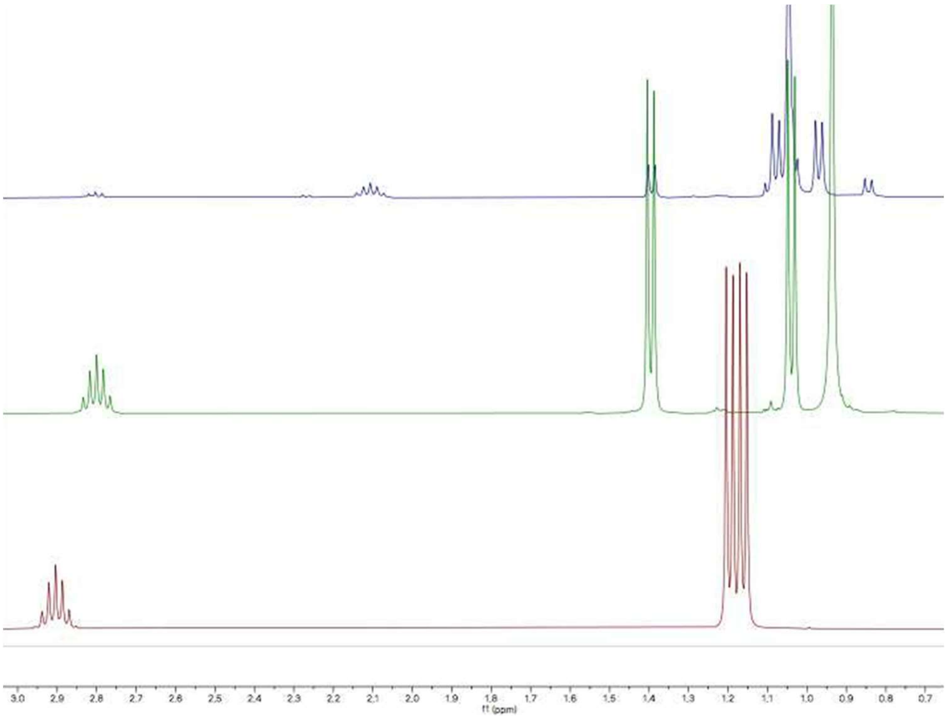

Figure S35: Stacked ^{1}H NMR spectra of the *in-situ* production of **8**. IDipp in PhCl (red). IDipp + HBPin in PhCl (green). IDipp + HBPin + $[CPh_3][B(C_6F_5)_4]$ in PhCl (blue).

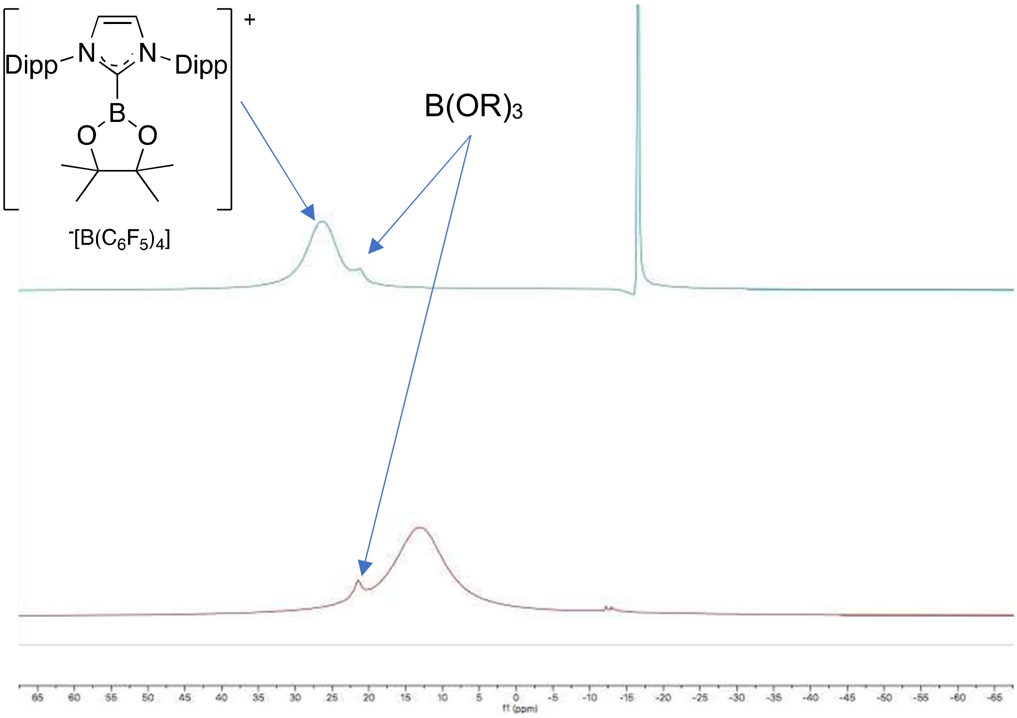

Figure S36: Stacked ^{11}B NMR spectra of the *in-situ* production of **8**. IDipp + HBPin in PhCl (red). IDipp + HBPin + $[CPh_3][B(C_6F_5)_4]$ in PhCl (turquoise).

S4.2 IDipp as a catalyst

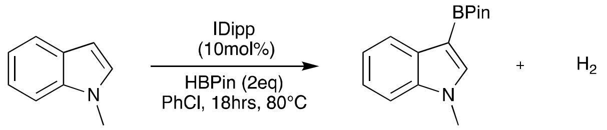

2a 3a

A J Youngs NMR tube was charged with solid IDipp (20 mg, 0.05 mmol), the solid was then dissolved in PhCl (500 μL) before addition of HBPin (145 μL, 1.00 mmol) and **2a** (63 μL, 0.50 mmol). The reaction was then heated to 80 °C, analysis of the reaction mixture after 18 hours by ^{1}H and ^{11}B NMR spectroscopy showed no conversion of substrate.

S4.3 BH_3•THF as a catalyst

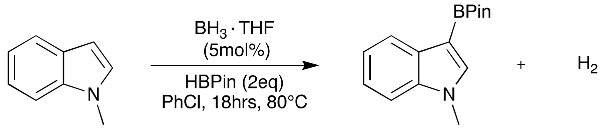

2a 3a

BH_3•THF (25 μL, 1.0 M, 0.025 mmol) was added to a J Youngs tube followed by PhCl (500 μL), HBPin (145 μL, 1.00 mmol) and **2a** (63 μL, 0.50 mmol). The reaction was then heated to 80 °C, analysis of the reaction mixture after 18 hours by ^{1}H and ^{11}B NMR spectroscopy showed no conversion of substrate.

S4.4 $Zn(C_6F_5)_2$ as a catalyst

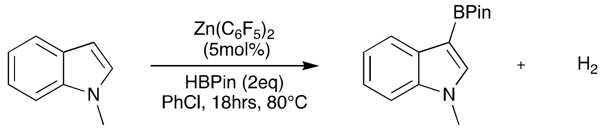

2a 3a

A J Youngs NMR tube was charged with $Zn(C_6F_5)_2$ (10 mg, 0.25mmol), the solid was then dissolved in PhCl (500 μL) before addition of HBPin (145 μL, 1.00 mmol) and **2a** (63 μL, 0.50 mmol). Shortly after addition of the reactants a white precipitate deposited at the bottom of the tube. The reaction was then heated to 80 °C for 18 hours, analysis by ^{1}H, ^{11}B and ^{19}F NMR spectroscopy showed no conversion of substrate, consumption of $Zn(C_6F_5)_2$ and formation of PinB-C_6F_5.

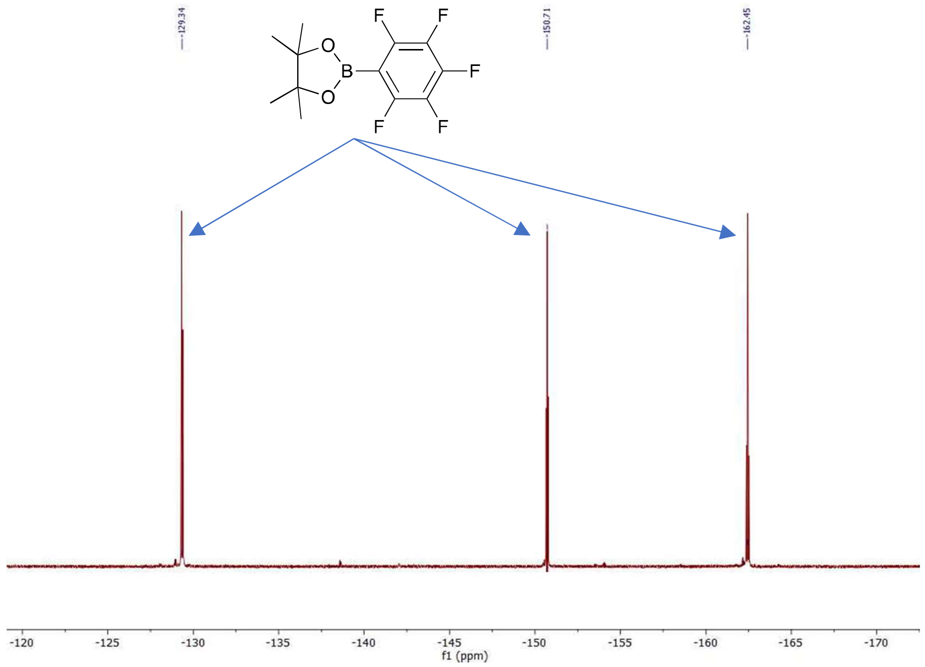

Figure S37: Attempted borylation of **2a** using $Zn(C_6F_5)_2$, metathesis observed by ^{19}F NMR spectroscopy.

S5. Metathesis studies

S5.1 Zn-Et/H-BPin metathesis

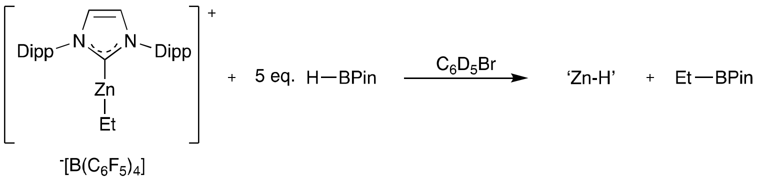

A J Young's NMR tube was charged with **5** (29 mg, 0.025 mmol), the solid was then dissolved in C_6D_5Br (500 μL) before addition of HBPin (18 μL, 0.125 mmol, 5eq.). The reaction was analysed by ^1H NMR spectroscopy upon addition of HBPin, after 24 hrs at room temperature and 1 week at room temperature. Isolation of a [IDippZnH][$B(C_6F_5)_4$] proved elusive in our hands despite multiple attempts at crystallisation.

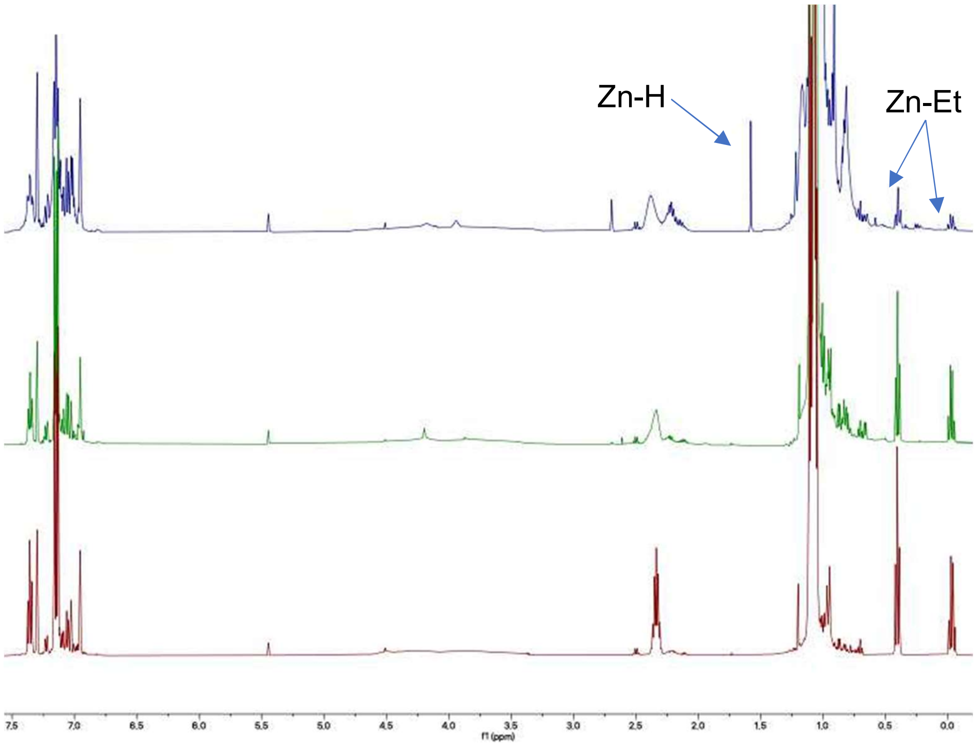

Figure S38: Stacked ^1H NMR spectra of the reaction between **5** and HBPin in C_6D_5Br. Immediately after addition of HBPin (red). After 24 hours at room temperature (green). After 1 week at room temperature (blue).

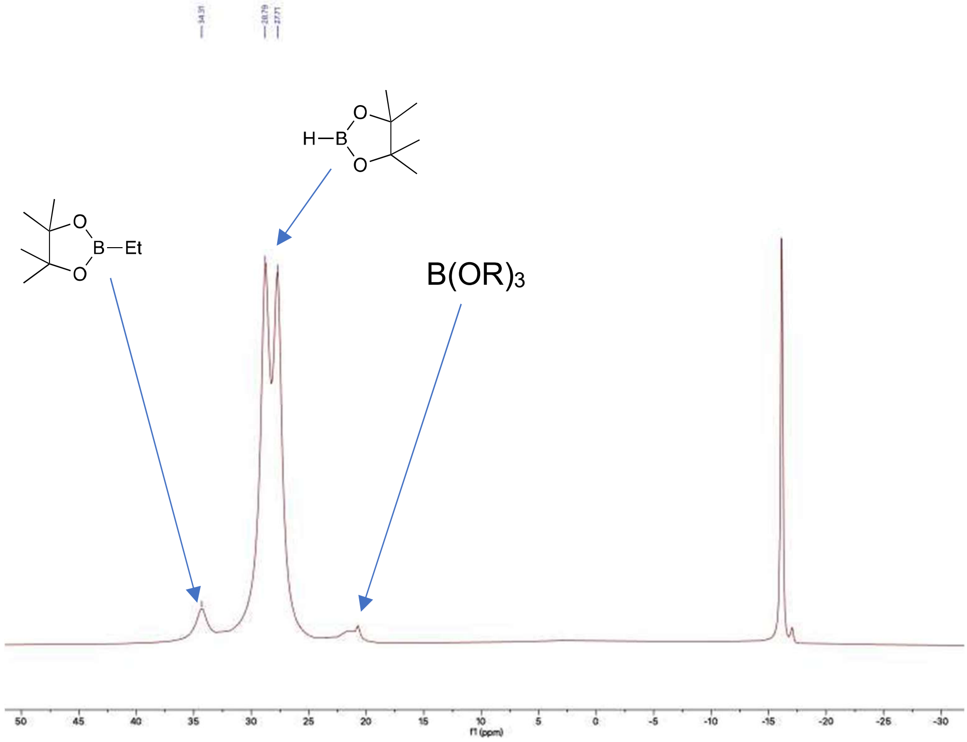

Figure S39: ^{11}B NMR spectrum of the reaction between **5** and HBPin in C_6D_5Br after 24 hours at room temperature.

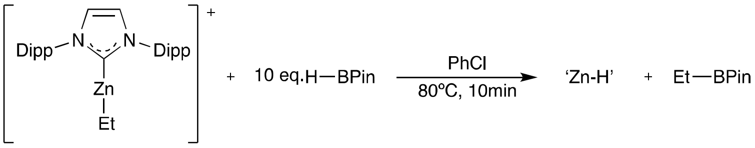

$^{-}$[B(C_6F_5)$_4$]

5

A J Young's NMR tube was charged with **5** (29 mg, 0.025 mmol), the solid was then dissolved in PhCl (500 μL) before addition of HBPin (36 μL, 0.25 mmol, 10eq.), the reaction was then heated at 80 °C for 10 minutes and analysed by ^{11}B NMR spectroscopy.

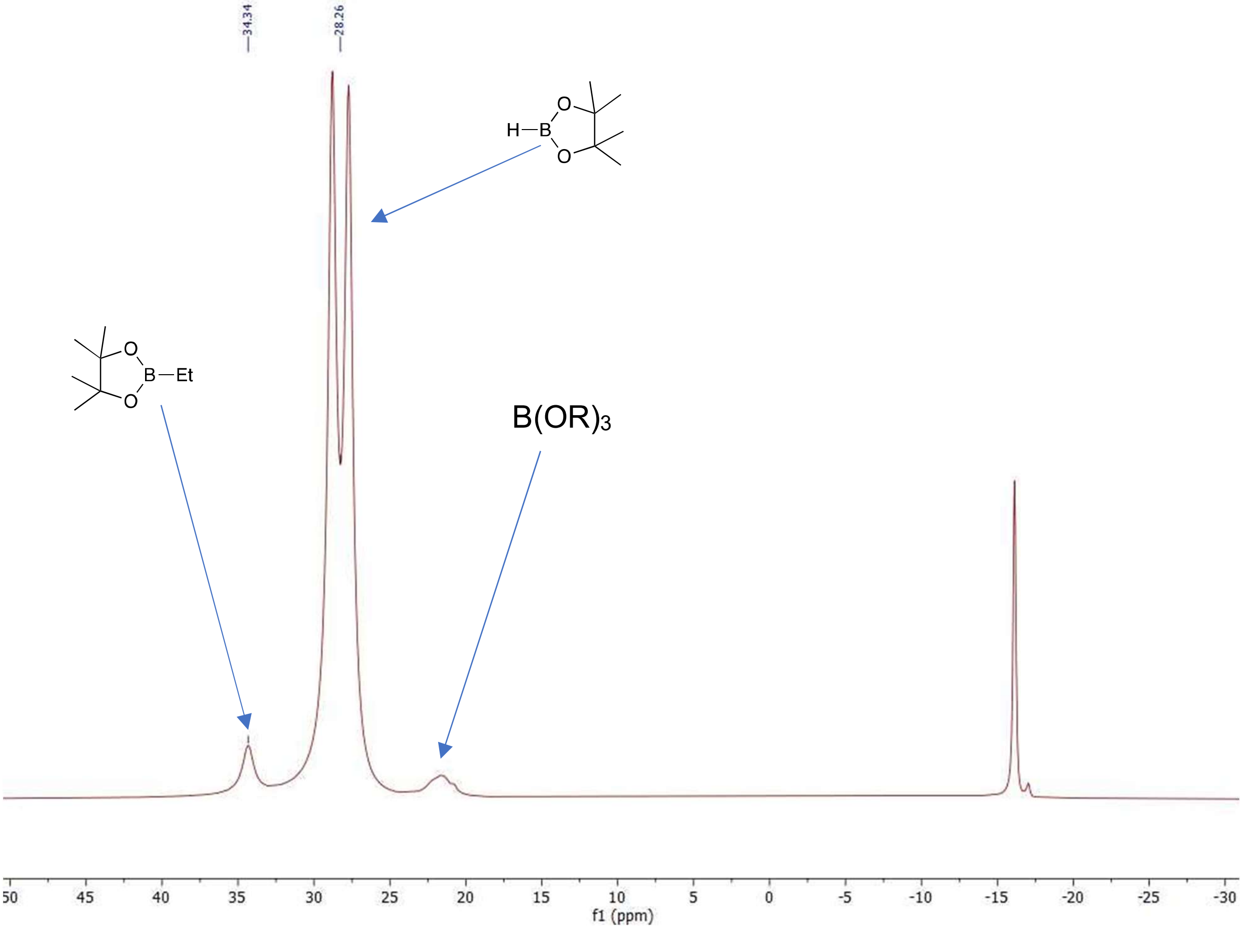

Figure S40: Reaction between **5** and HBPin after 10 mins at 80 °C as observed by ^{11}B NMR spectroscopy.

Mass spectrometry analysis of reaction mixture

A J Young's NMR tube was charged with **5** (29 mg, 0.025 mmol), the solid was then dissolved in PhCl (500 μL) before addition of HBPin (18 μL, 0.125 mmol, 5eq.). This was then left for 4 days at room temperature before analysis by mass spectrometry using electrospray ionisation where [IDippZnH(H_2O)]$^+$, Calculated [M^+]: 471.24, Observed [M^+]: 471.3 was observed. Peaks for solvent free or oligomeric NHCZn products were not observed.

S5.2 Zn-Et/D-BPin metathesis

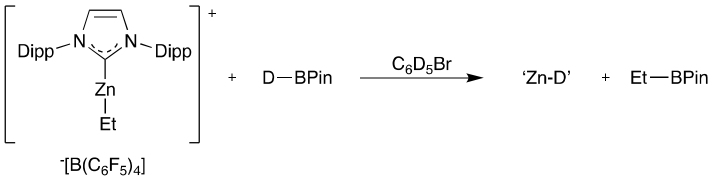

A J Young's NMR tube was charged with **5** (29 mg, 0.025 mmol), the solid was then dissolved in PhCl (500 μL) before addition of DBPin (3.7 μL, 0.025 mmol, 1eq.). The reaction was analysed by ^{1}H NMR spectroscopy after 24 hrs and 1 week at room temperature, an additional equivalent of DBPin (3.7 μL, 0.025 mmol) was then added and the reaction analysed by ^{1}H NMR spectroscopy after a further 3 weeks at room temperature. Isolation of a [NHCZnD][B(C_6F_5)$_4$] species proved elusive in our hands.

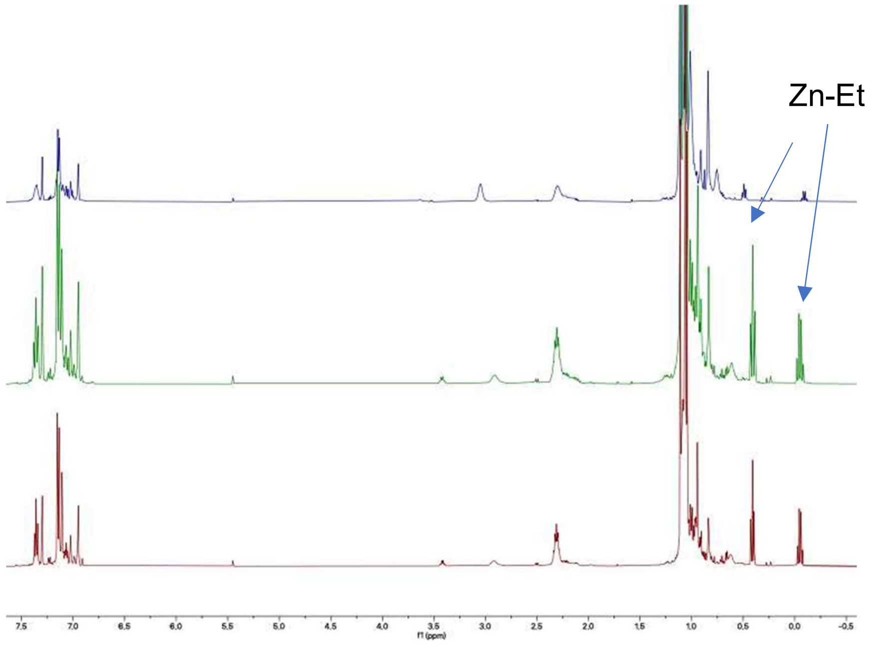

Figure S41: Stacked ^{1}H NMR spectra of the reaction between **5** and DBPin in C_6D_5Br. After 24 hours at room temperature (red). After 1 week at room temperature (green). 3 weeks after addition of 2nd equivalent of DBPin (blue).

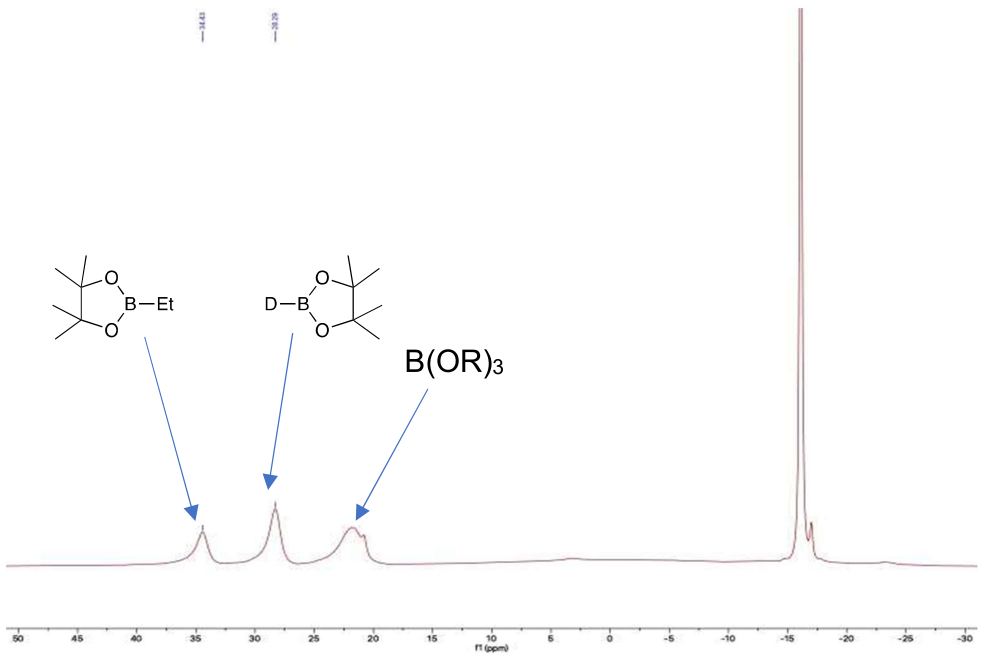

Figure S42: ^{11}B NMR spectrum of the reaction between **5** and DBPin in C_6D_5Br after 24 hours at room temperature.

S5.3 Zn-Et/H-BCat metathesis

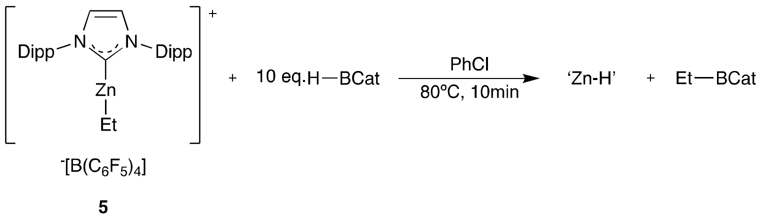

A J Young's NMR tube was charged with **5** (29 mg, 0.025 mmol), the solid was then dissolved in PhCl (500 μL) before addition of HBCat (26 μL, 0.25 mmol, 10eq.), the reaction was then heated at 80 °C for 10 minutes and analysed by ^{11}B NMR spectroscopy.

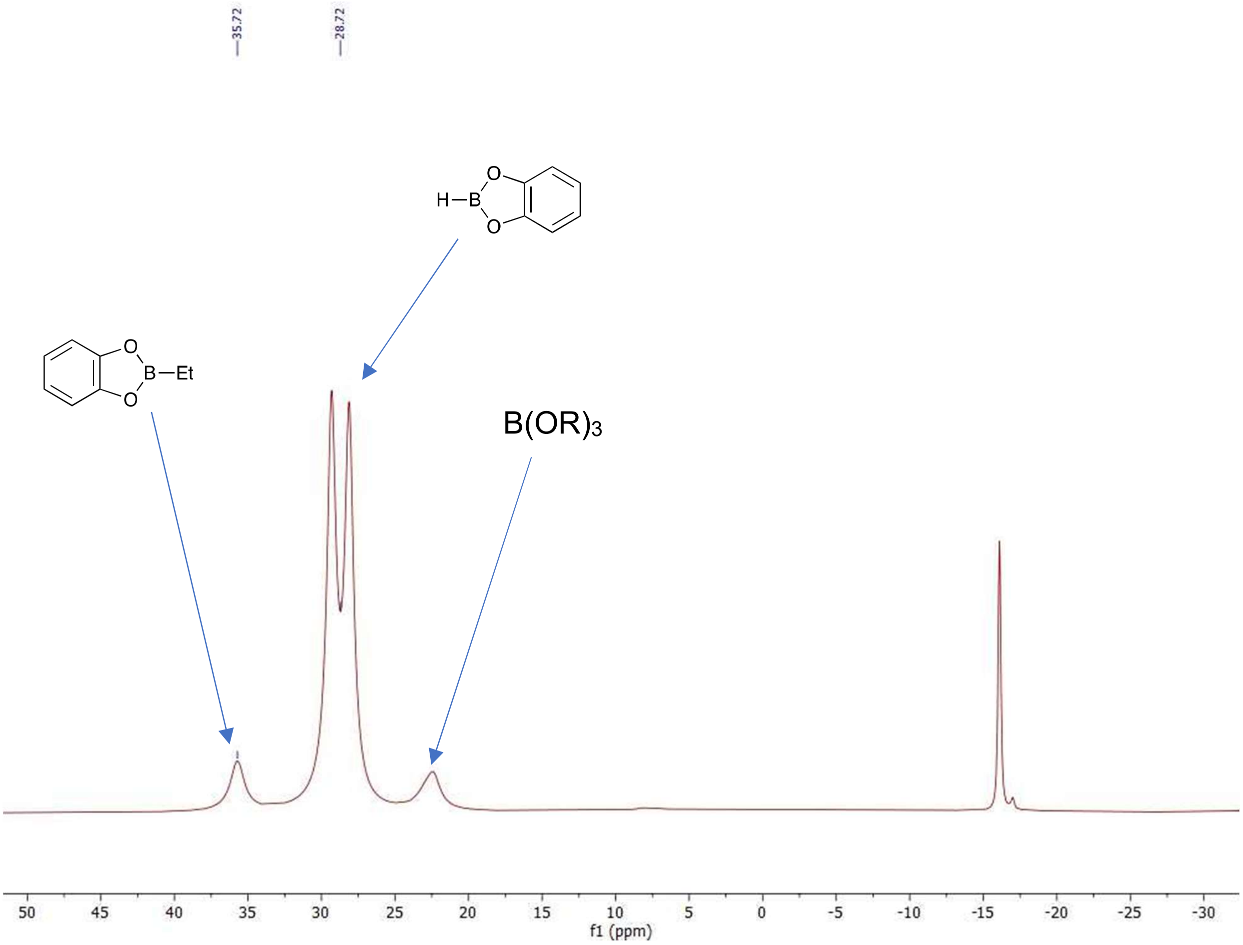

Figure S43: Reaction between **5** and HBCat after 10 mins at 80 °C as observed by ^{11}B NMR spectroscopy.

S5.4 Zn-Et/H-9BBN metathesis

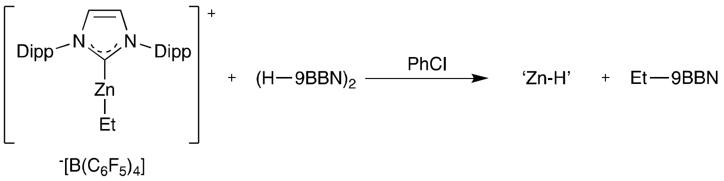

5

A J Young's NMR tube was charged with **5** (29 mg, 0.025 mmol), the solid was then dissolved in PhCl (500 μL) before addition of HBBN (6.1 mg, 0.025 mmol, 1eq.). The reaction was analysed by ^{1}H NMR spectroscopy after addition of HBBN and 24 hours at room temperature, an additional equivalent of HBBN (6.1 mg, 0.025mmol, 1eq.) was then added and the reaction analysed by ^{1}H NMR spectroscopy after a further week at room temperature. Isolation of a [IDippZnH][$B(C_6F_5)_4$] from these reaction mixtures proved elusive in our hands.

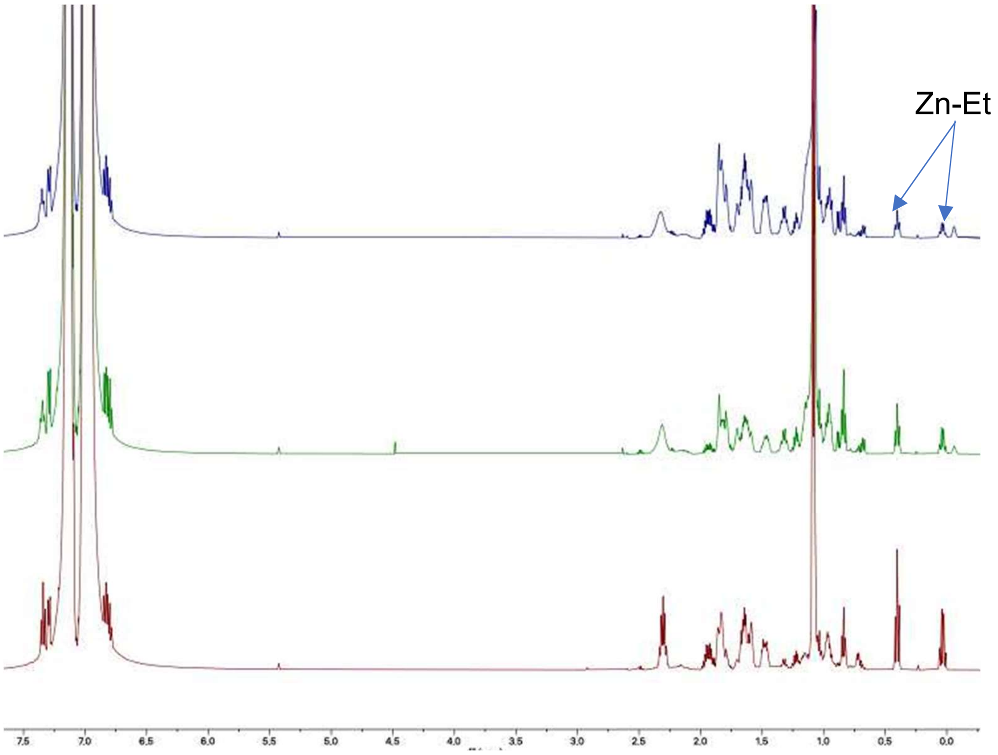

Figure S44: Stacked ^{1}H NMR spectra of the reaction between **5** and HBBN in C_6D_5Br. Immediately after addition of HBBN (red). After 24 hours at room temperature (green). 1 week after addition of 2nd equivalent of HBBN (blue).

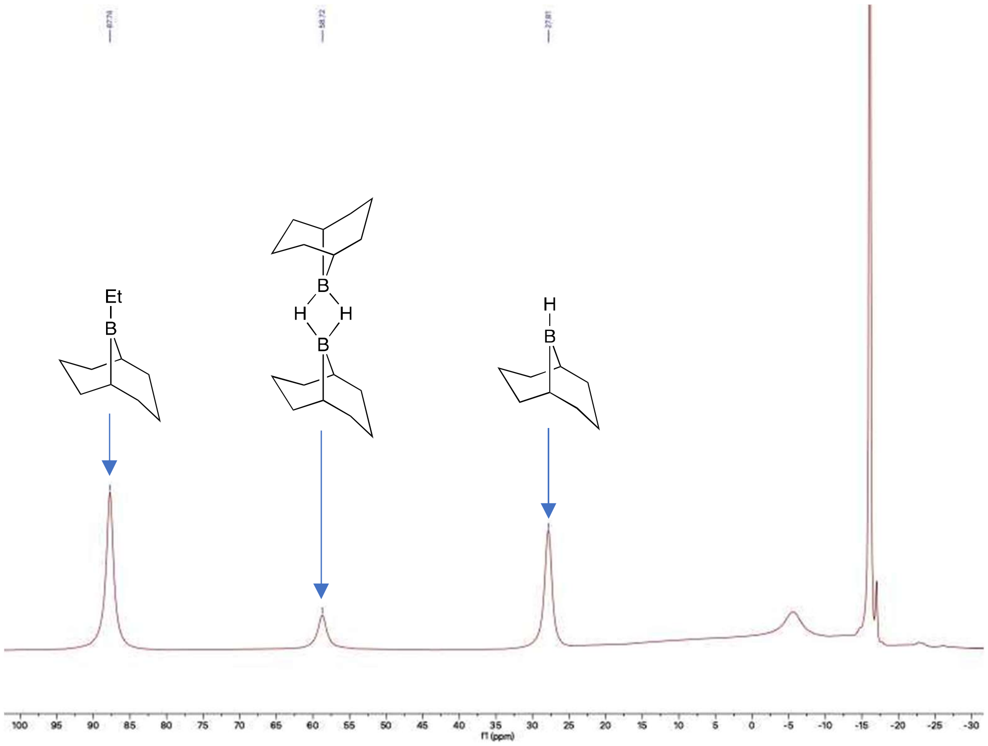

Figure S45: ^{11}B NMR spectrum of the reaction between **5** and HBBN in PhCl after 24 hours at room temperature.

S5.5 Attempted indole metalation with 1-H

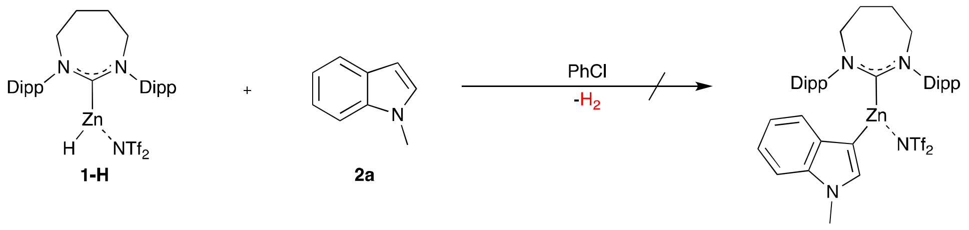

A J Young's NMR tube was charged with 7DippZnH(NTf_2) (**1-H**) (19 mg, 0.025 mmol), the solid was then dissolved in PhCl (500 µL) aided by sonication at room temperature before addition of **2a** (3.1 µL, 0.025 mmol). Analysis by ^1H NMR spectroscopy showed no reaction after heating at 60 °C for 1 hour. The temperature was then increased to 100 °C in line with the catalytic conditions for **1-Ph**, however no reaction was observed by ^1H NMR spectroscopy after 24 hours.

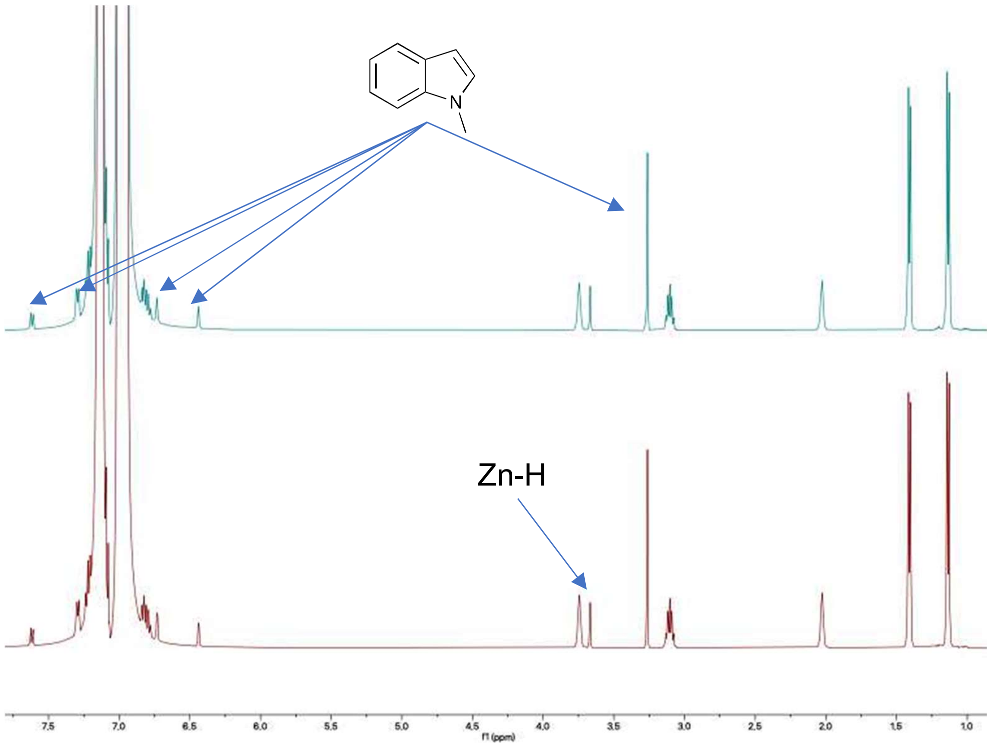

Figure S46: Stacked ^1H NMR spectra of the reaction between **1-H** and N-Me-indole in PhCl. Immediately after addition of N-Me-indole (red). After 24 hours at 100 °C (green).

S5.6 Zn-C_6F_5 / H-BPin metathesis under catalytic conditions

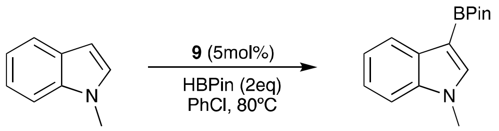

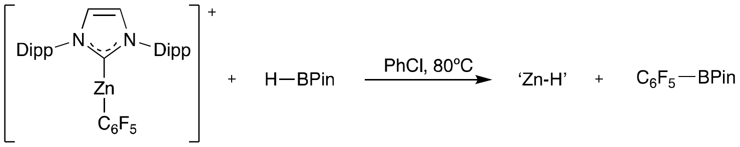

$^{-}[B(C_6F_5)_4]$
9

A J Young's NMR tube was charged with **9** (32 mg, 0.025 mmol), the solid was then dissolved in PhCl (500 μL) before addition of HBPin (18 μL, 0.125 mmol, 5eq.). The reaction was then heated at 80 ºC, the N-Me-indole borylation and Zn-C_6F_5/H-BPin metathesis reactions were then monitored periodically by ^{1}H and ^{19}F NMR spectroscopy respectively (**Table S4**).

Table S4 – Conversion of substrate and % of metathesis by-product at various time intervals during catalytic C-H borylation of N-Me-indole by **9**.

Time/hrs	Conversion of substrate / %[a]	% PinB-C_6F_5[b]
1	13	1
4	26	2
12	57	4
15	65	5
18	72	6

[a]: determined *in-situ* by ^{1}H NMR spectroscopy. [b]: determined *in-situ* by ^{19}F NMR spectroscopy.

S5.7 Zn-C_6F_5 / H-BCat metathesis

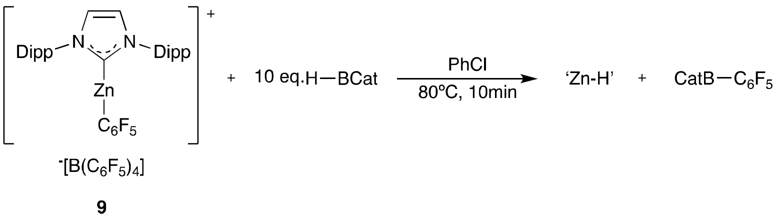

A J Young's NMR tube was charged with **9** (32 mg, 25 μmmol), the solid was then dissolved in PhCl (500 μL) before addition of HBCat (26 μL, 0.25 mmol, 10eq.), the reaction was then heated at 80 ºC for 10 minutes and analysed by ^{19}F NMR spectroscopy.

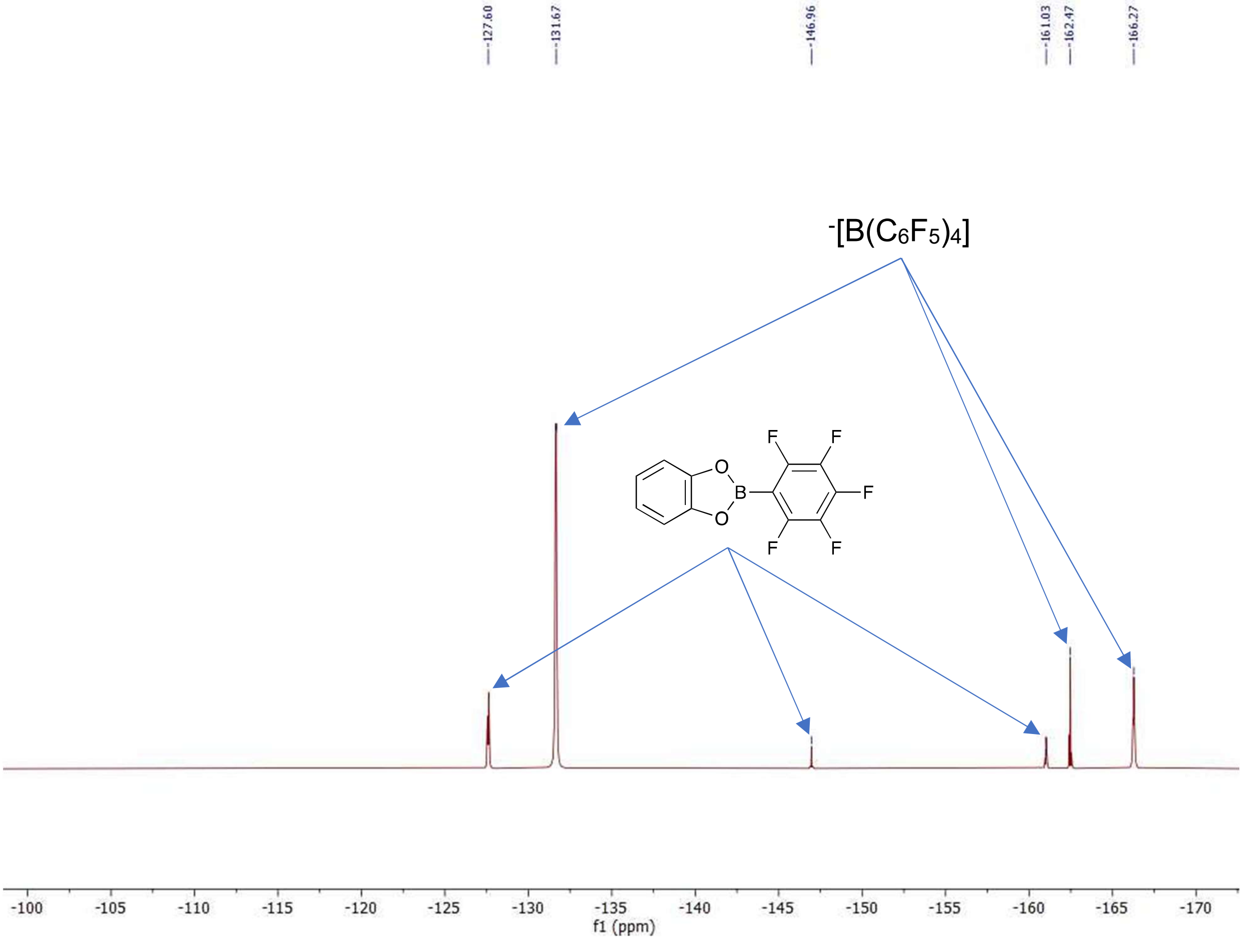

Figure S47: Reaction between **9** and HBCat after 10 mins at 80 ºC as observed by ^{11}B NMR spectroscopy.

S6. [IDippZnEt][B(C_6F_5)$_4$] / DMT study

S6.1 [IDippZnEt-(DMT)$_x$][B(C_6F_5)$_4$] adduct

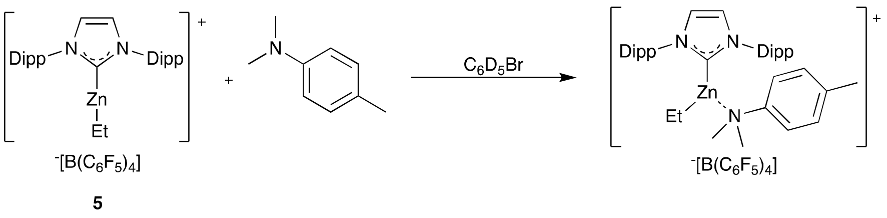

A J Young's NMR tube was charged with **5** (29 mg, 0.025 mmol, 1eq.) this was then dissolved in d_5-bromobenzene (500 μL) before addition of N,N-dimethyl-*p*-toluidine (2.0 μL, ca. 0.5 eq.) and analysis by ^{1}H NMR spectroscopy, two subsequent portions of N,N-dimethyl-*p*-toluidine (4.0 μL, ca. 1 eq.) were added with ^{1}H NMR measurements taken after each addition. Repeated attempts to isolate the product and to observe the adduct by air sensitive mass spectrometry using electrospray ionisation proved unsuccessful in our hands.

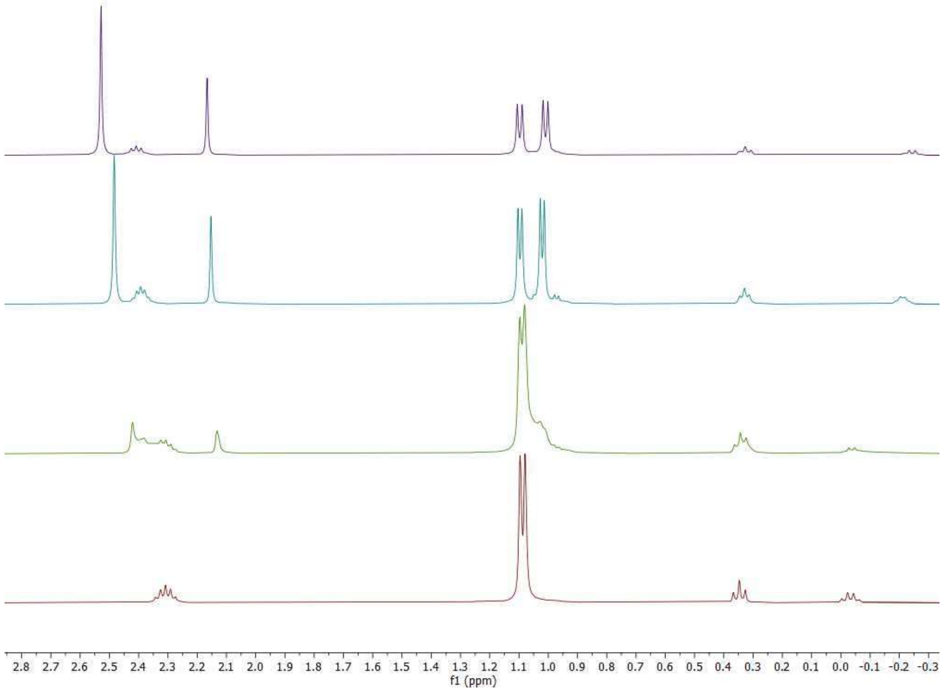

Figure S48: Stacked ^{1}H NMR spectra of **5** in C_6D_5Br. Prior to the addition of DMT (red). After addition of 0.5 eq. DMT (green). After addition of 1.5 eq. DMT (blue). After addition of 2 eq. DMT (purple).

S6.2 HBPin metathesis of [IDippZnEt-(DMT)$_x$][B(C_6F_5)$_4$]

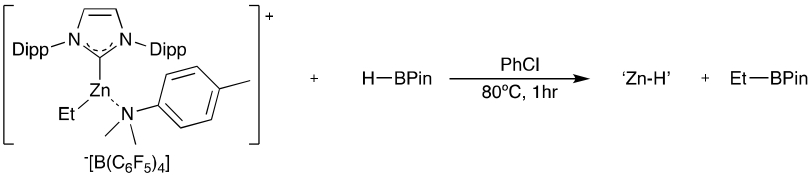

A J Young's NMR tube was charged with **5** (29 mg, 0.025 mmol, 1eq.) this was then dissolved in PhCl (500 μL) before addition of N,N-dimethyl-*p*-toluidine (3.7 μL, 0.025 mmol, 1eq.) and pinacolborane (3.6 μL, 0.025 mmol, 1eq.). The reaction mixture was then heated at 80 °C for 1 hour before analysis by ^{1}H NMR spectroscopy.

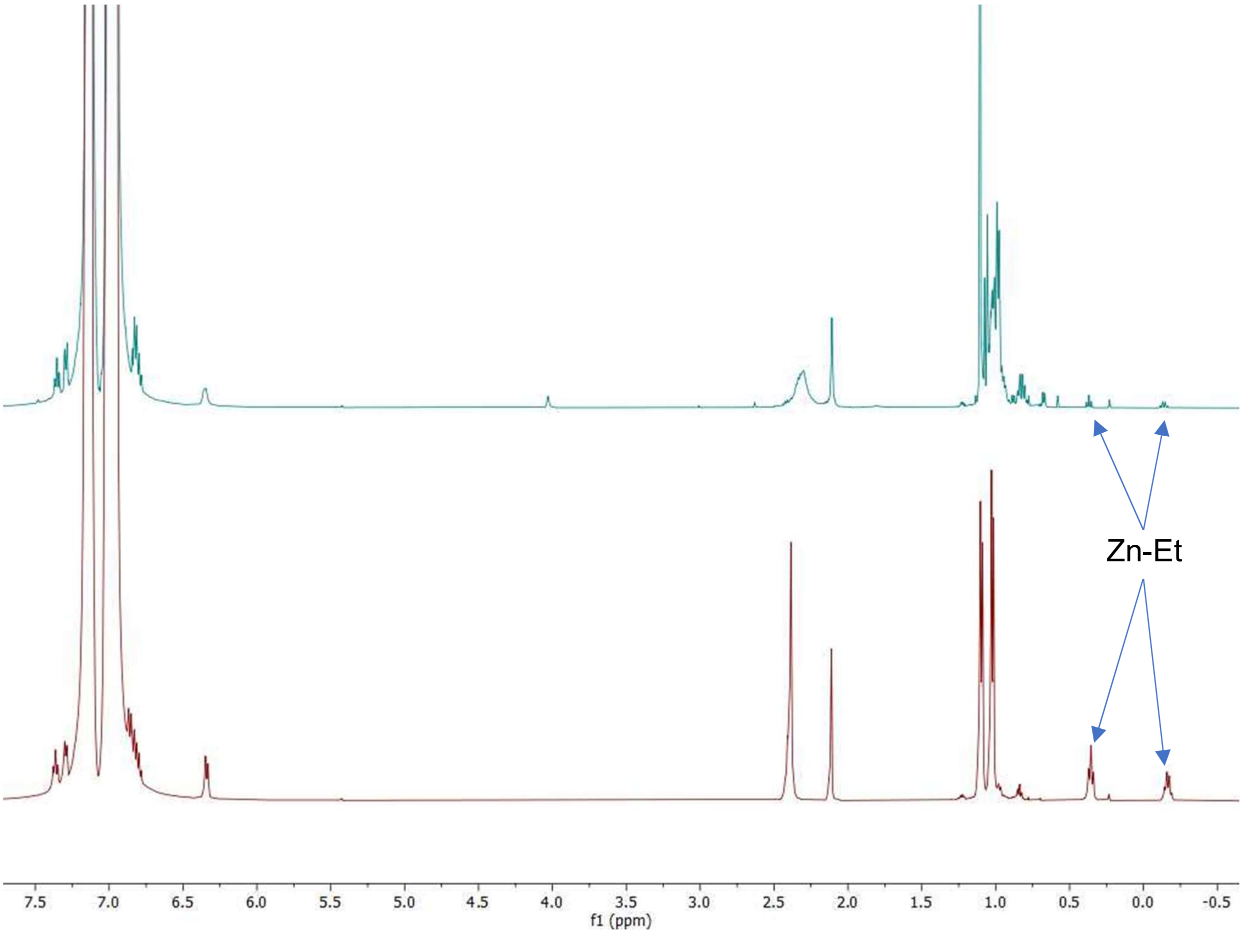

Figure S49: Stacked ^{1}H NMR spectra of putative [IDippZnEt-(DMT)$_x$][B(C_6F_5)$_4$] in PhCl. Before addition of HBPin (red). After addition of HBPin and heating at 80 °C for 1 hour (green).

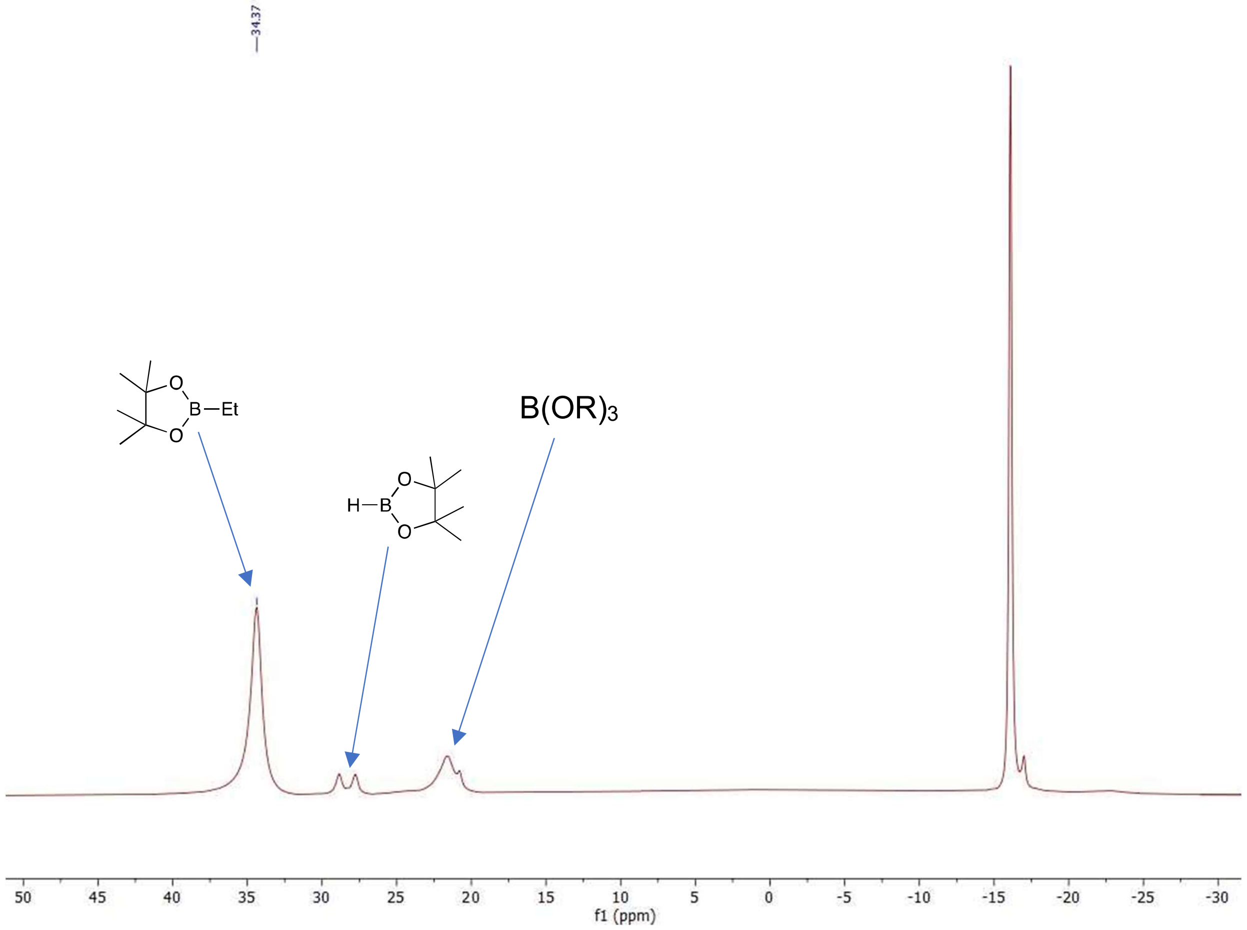

Figure S50: Reaction of putative [IDippZnEt-(DMT)$_x$][B(C_6F_5)$_4$] and HBPin in PhCl after heating at 80 ºC for 1 hour as observed by ^{11}B NMR spectroscopy.

S6.3 Attempted indole metalation with putative [IDippZnEt-(DMT)$_x$][B(C$_6$F$_5$)$_4$]

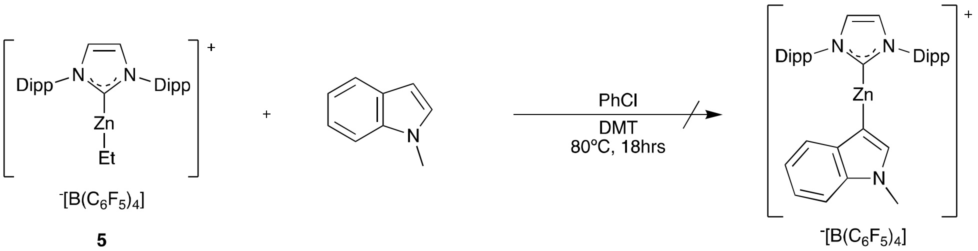

A J Young's NMR tube was charged with **5** (29 mg, 0.025 mmol, 1eq.) this was then dissolved in PhCl (500 µL) before addition of N,N-dimethyl-*p*-toluidine (3.7 µL, 0.025 mmol, 1eq.) and **2a** (3.1 µL, 0.025 mmol, 1eq.). The reaction mixture was then heated at 80 ºC for 18 hours before analysis by ^{1}H NMR spectroscopy.

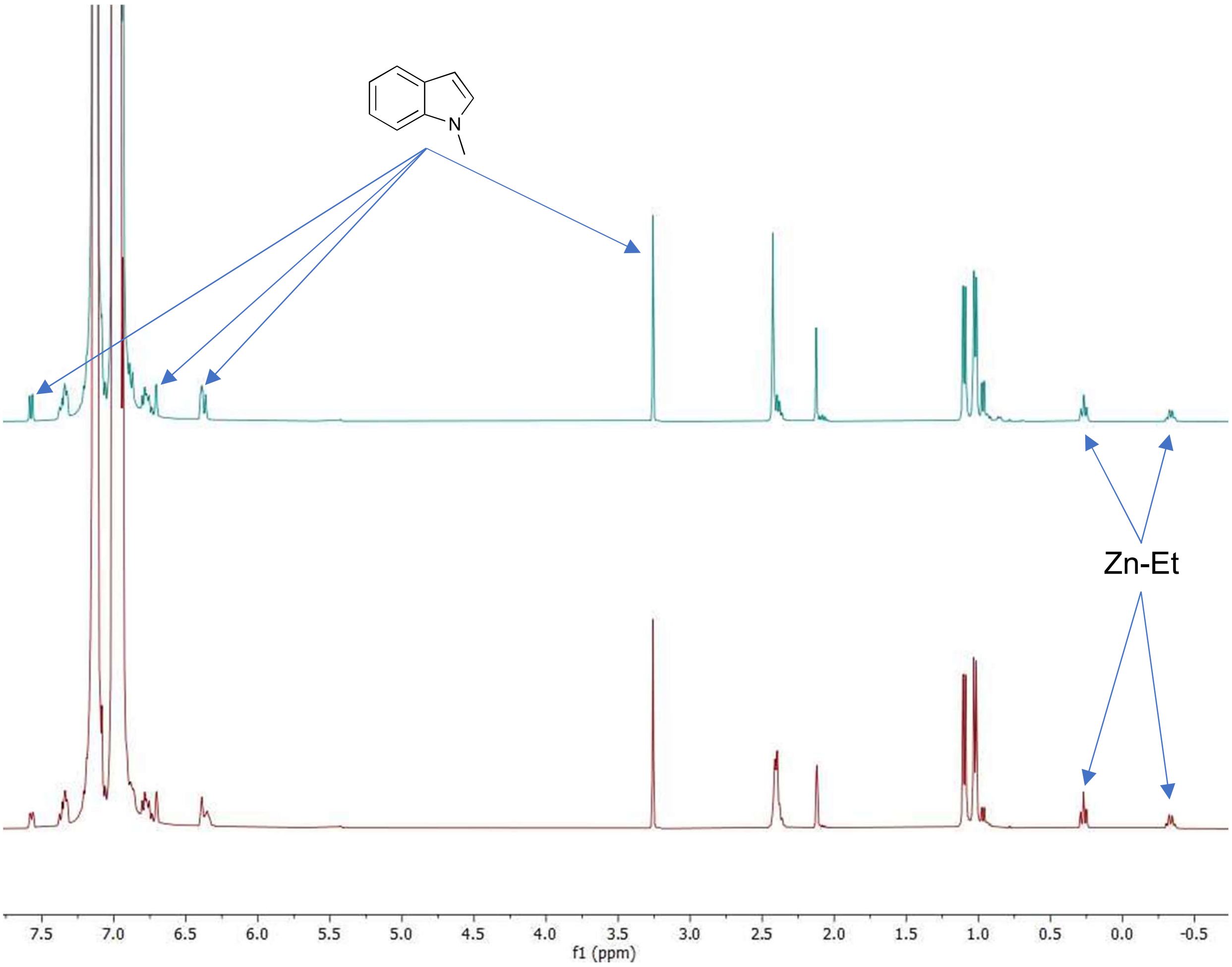

Figure S51: Stacked ^{1}H NMR spectra of **5**, DMT and **2a** in PhCl. Before heating (red). After heating at 80 ºC for 18 hours.

S7. $[IDippZnC_6F_5][B(C_6F_5)_4]$ / HBPin interaction study

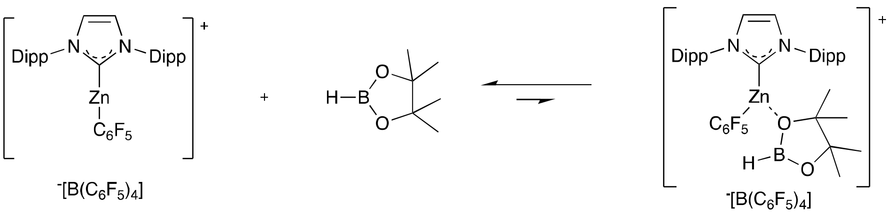

9 **9a**

A J Young's NMR tube was charged with **9** (24 mg, 0.018 mmol) before dissolution in PhCl (500 μL) and addition of HBPin (13 μL, 0.09 mmol), the reaction was then immediately analysed by ^{1}H, ^{11}B and ^{19}F NMR spectroscopy. ^{11}B NMR spectroscopy revealed a severe broadening of the usually well resolved doublet resonance at 28 ppm corresponding to H<u>B</u>Pin. Additionally, shifting of the Zn-C_6<u>F_5</u> resonances in the ^{19}F NMR spectrum (**Figure S52**) was observed upon addition of HBPin.

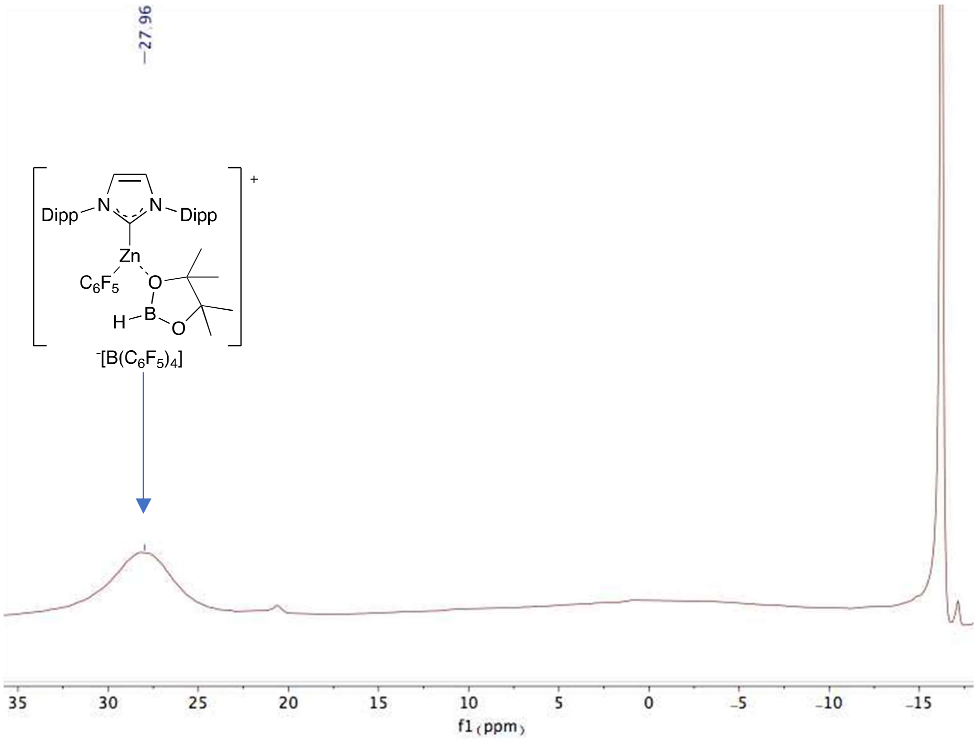

Figure S52 - ^{11}B NMR spectrum of **9** and HBPin in PhCl.

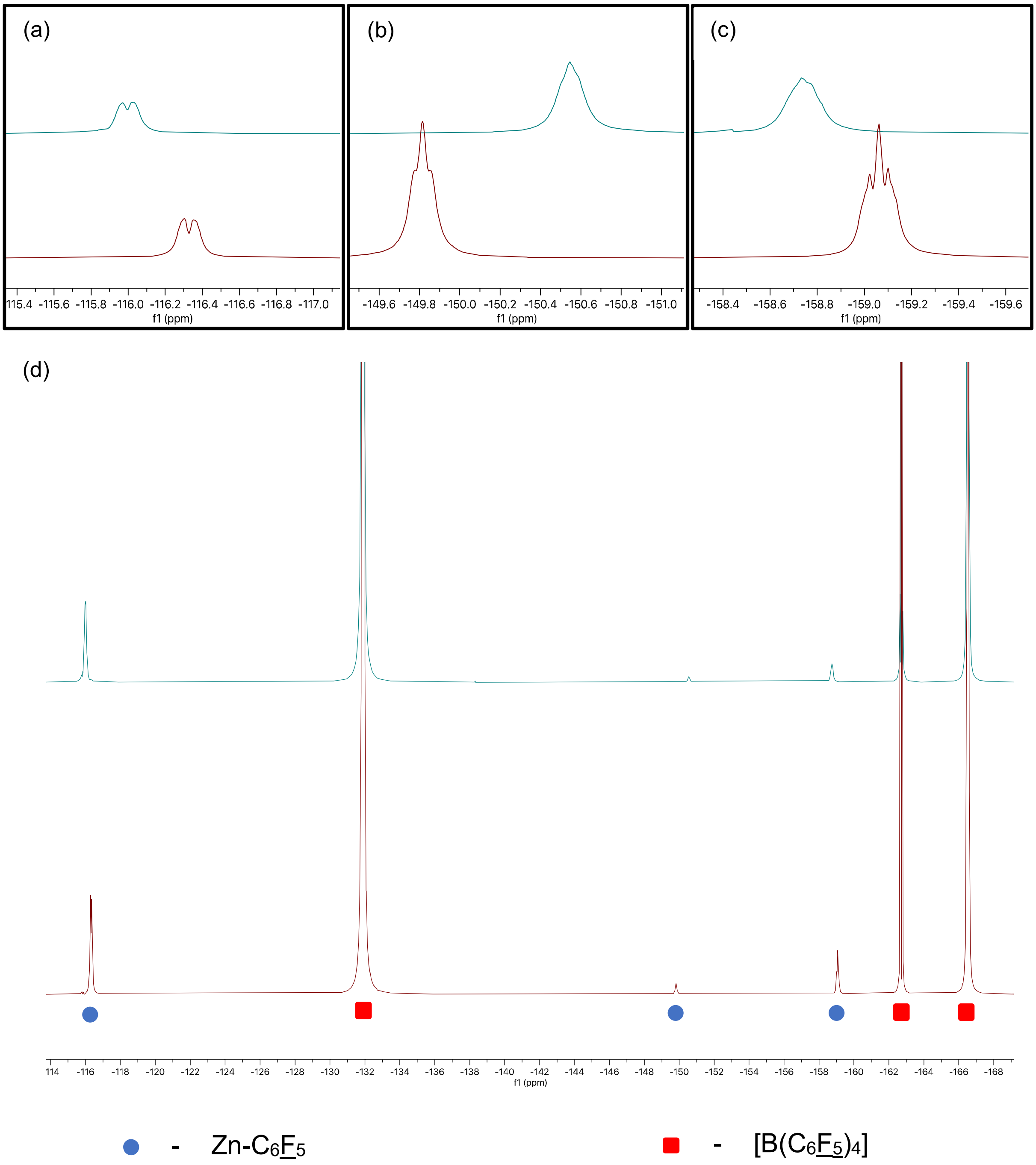

Figure S53: (a)Top left. Zoomed image of (d) in the region ca. -116 ppm. (b) Top centre. Zoomed image of (d) in the region ca. -150 ppm. (c) Top right. Zoomed image of (d) in the region ca. -159 ppm. (d) Stacked ^{19}F spectra of **9** in PhCl (red) and **9** after addition of HBPin in PhCl (green). Spectrum referenced to literature values for the anion.

Analysis of interaction by VT NMR spectroscopy: A J Young's NMR tube was charged with **9** (24 mg, 18 μmmol) before dissolution in PhCl (500 μL) and addition of HBPin (13 μL, 0.09 mmol) the interaction was then analysed by ^{19}F NMR spectroscopy at 27 ºC, 0 ºC, -10 ºC, -20 ºC, -30 ºC and -40 ºC. Two distinct sets of resonances were observed in the ^{19}F NMR spectra (Figures **S54-57**) at low temperature (-30 ºC to -40 ºC) which coalesce at higher temperatures. The sets of resonances in the low-temperature regime were assigned to the Zn-C_6F_5 resonances of **9** and the O-bound HBPin adduct **9a**. It should also be noted that a variable temperature ^{11}B NMR study was also conducted, however whilst decreasing the temperature sharpened the broadened H-BPin peak, neither the expected doublet nor any additional peaks could be resolved at the temperatures accessible with this solvent.

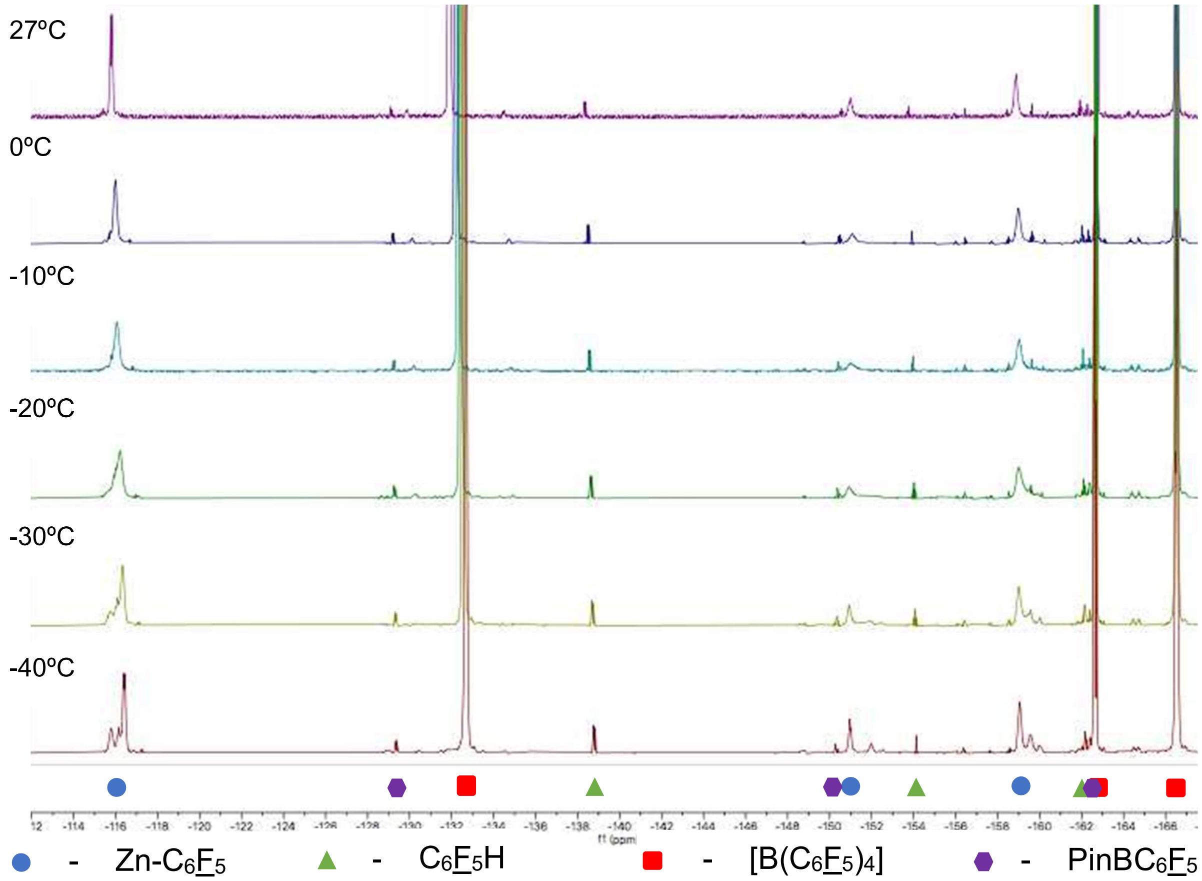

Figure S54: Stacked ^{19}F NMR spectra of reaction mixture of **9** and HBPin (5eq) in PhCl at various temperatures. Spectrum referenced to literature value for anion.

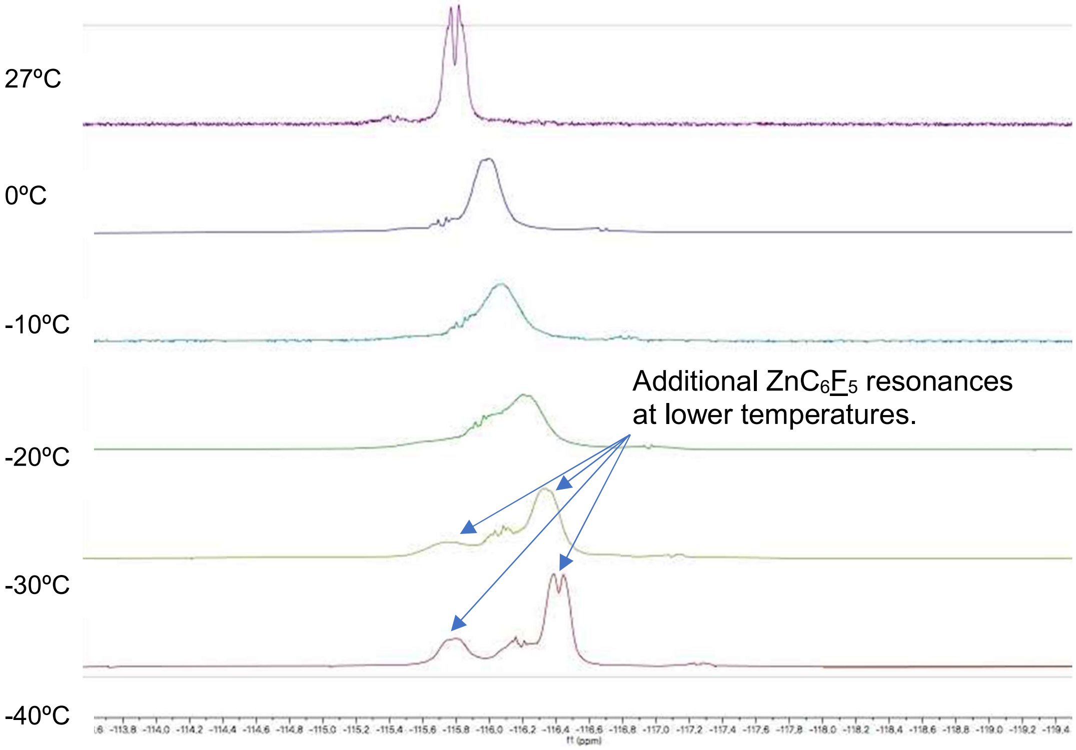

Figure S55: Zoomed image of **S54** in to the region ca. -116ppm.

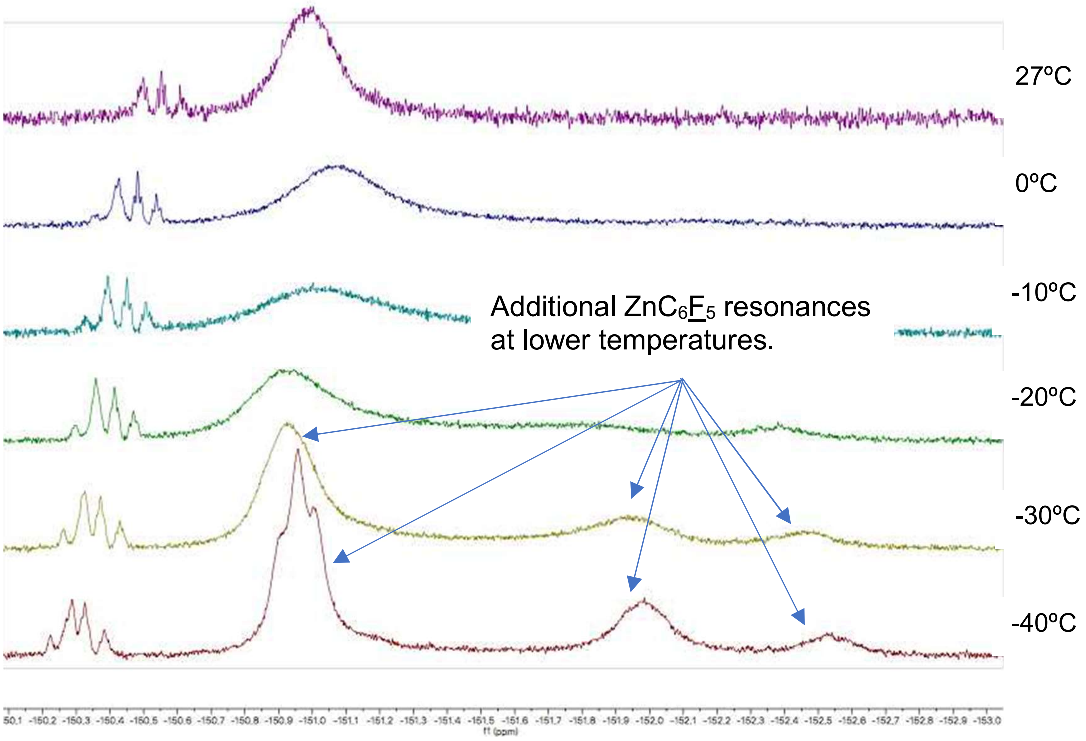

Figure S56: Zoomed image of **S54** in to the region ca. -151ppm.

VT NMR control experiment: A J Young's NMR tube was charged with **9** (24 mg, 18 μmmol) before dissolution in PhCl (500 μL) the solvated compound was then analysed by ^{19}F NMR spectroscopy at 27 ºC, 0 ºC, -10 ºC, -20 ºC, -30 ºC and -40 ºC.

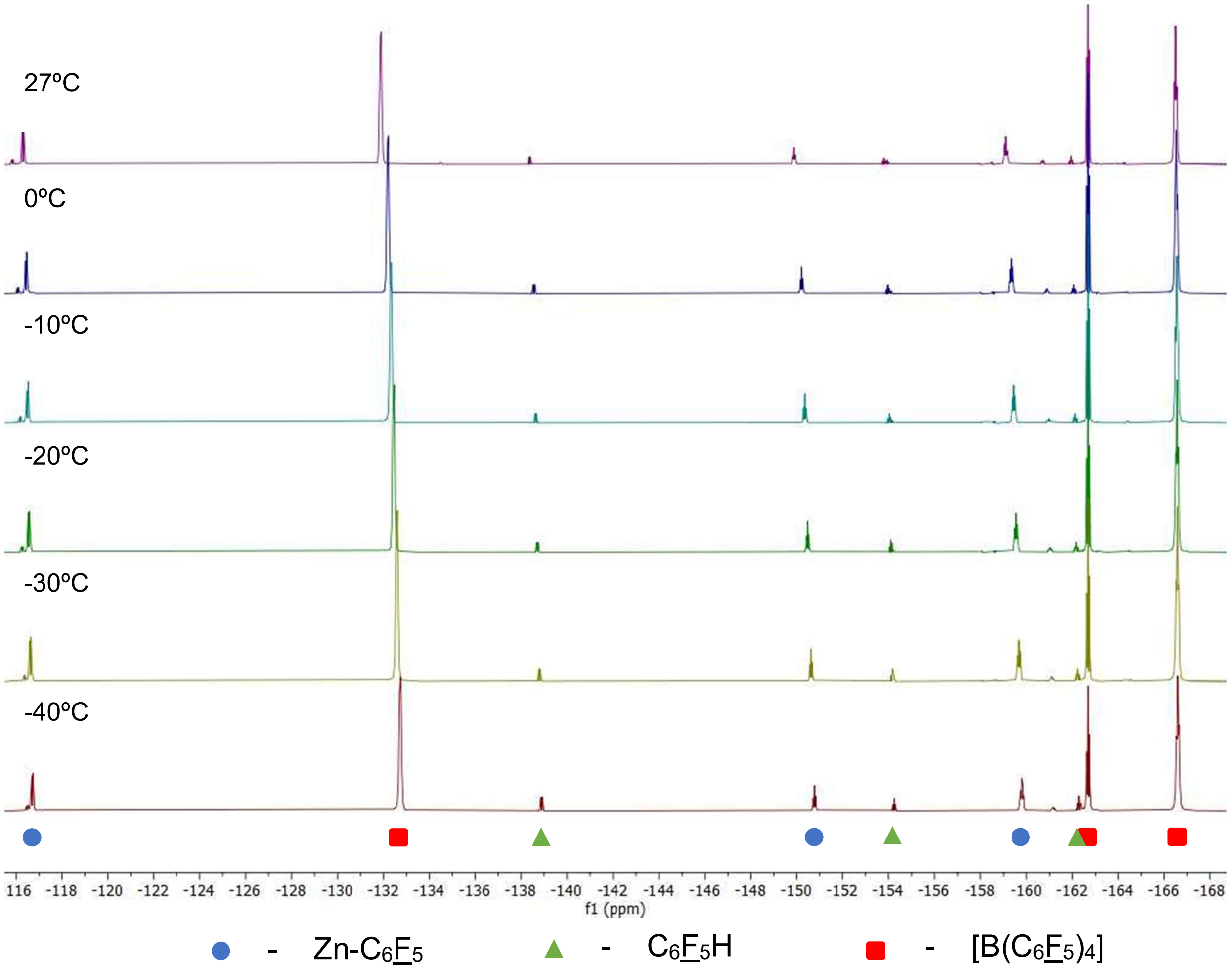

Figure S57: Stacked ^{19}F NMR of **9** solvated in PhCl at various temperatures.

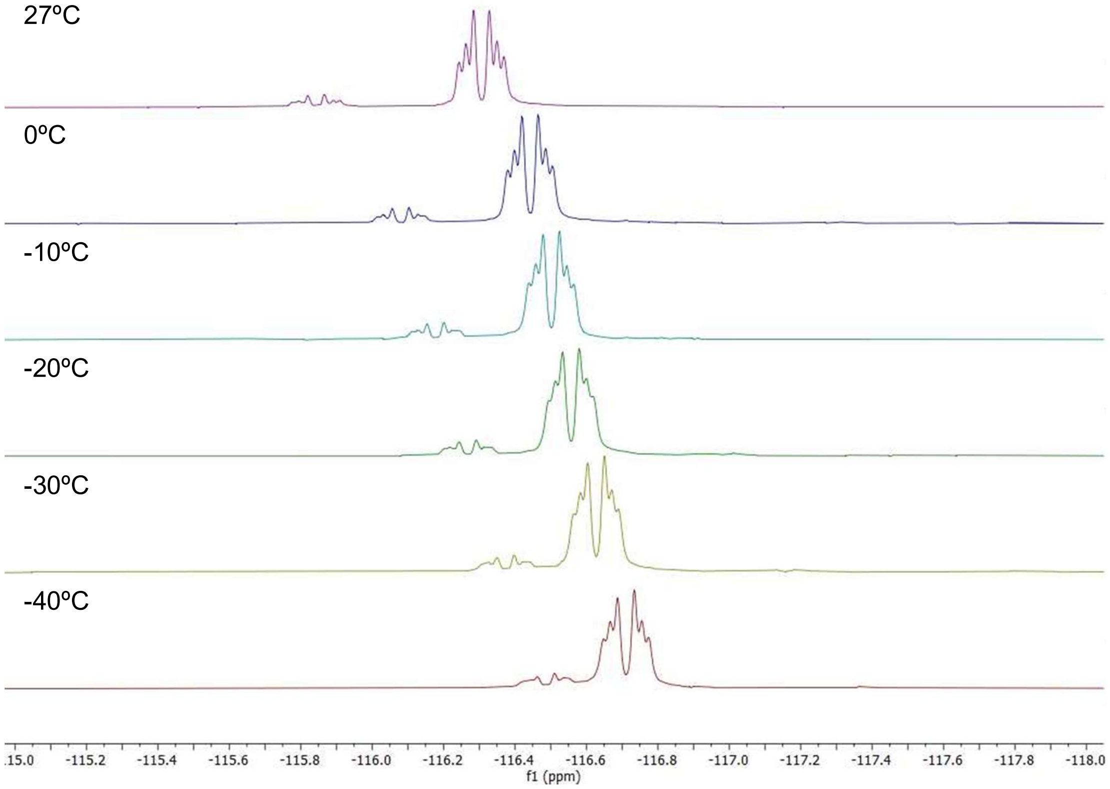

Figure S58: Zoomed image of **S57** in to the region ca. -116ppm.

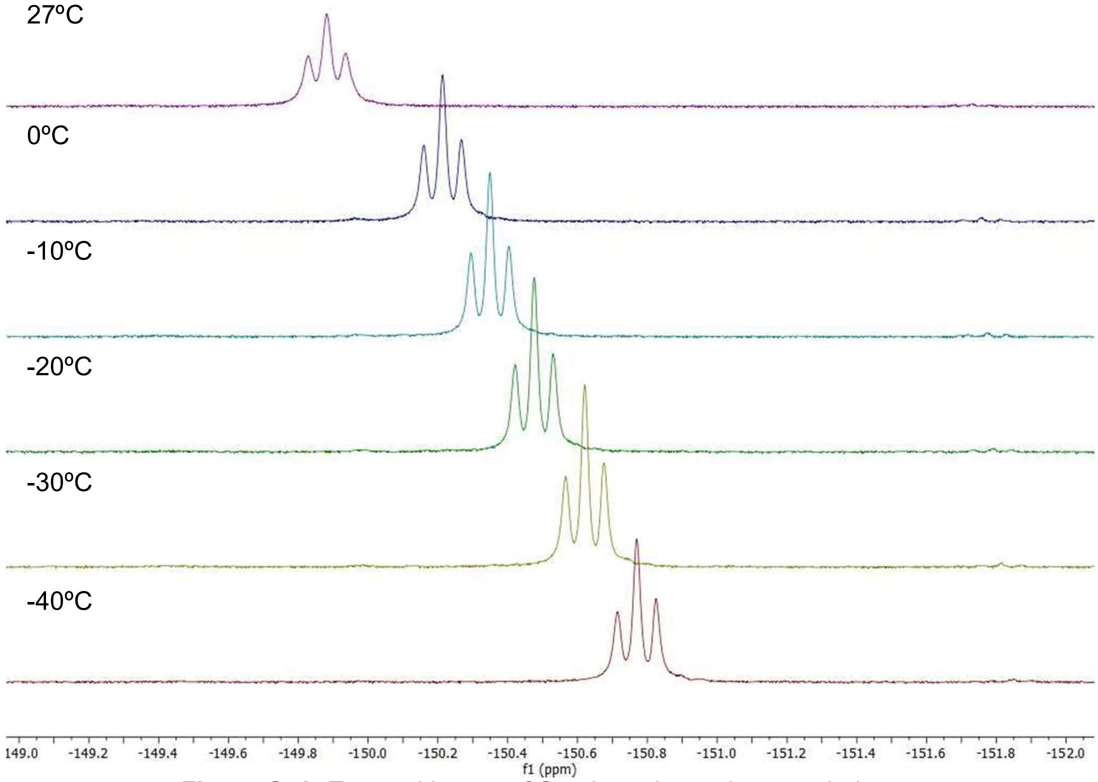

Figure S59: Zoomed image of **S57** in to the region ca. -151ppm.

S8. 7DippZnH(NTf_2) DBPin H/D exchange study

A J Young's NMR tube was charged with 7DippZnH(NTf_2) (**1-H**) (19 mg, 0.025 mmol), the solid was then dissolved in PhCl (500 μL) before addition of DBPin (3.6 μL, 0.025 mmol). Immediately after addition ^{1}H, ^{11}B and ^{2}H NMR spectroscopy was used to analyse the reaction mixture.

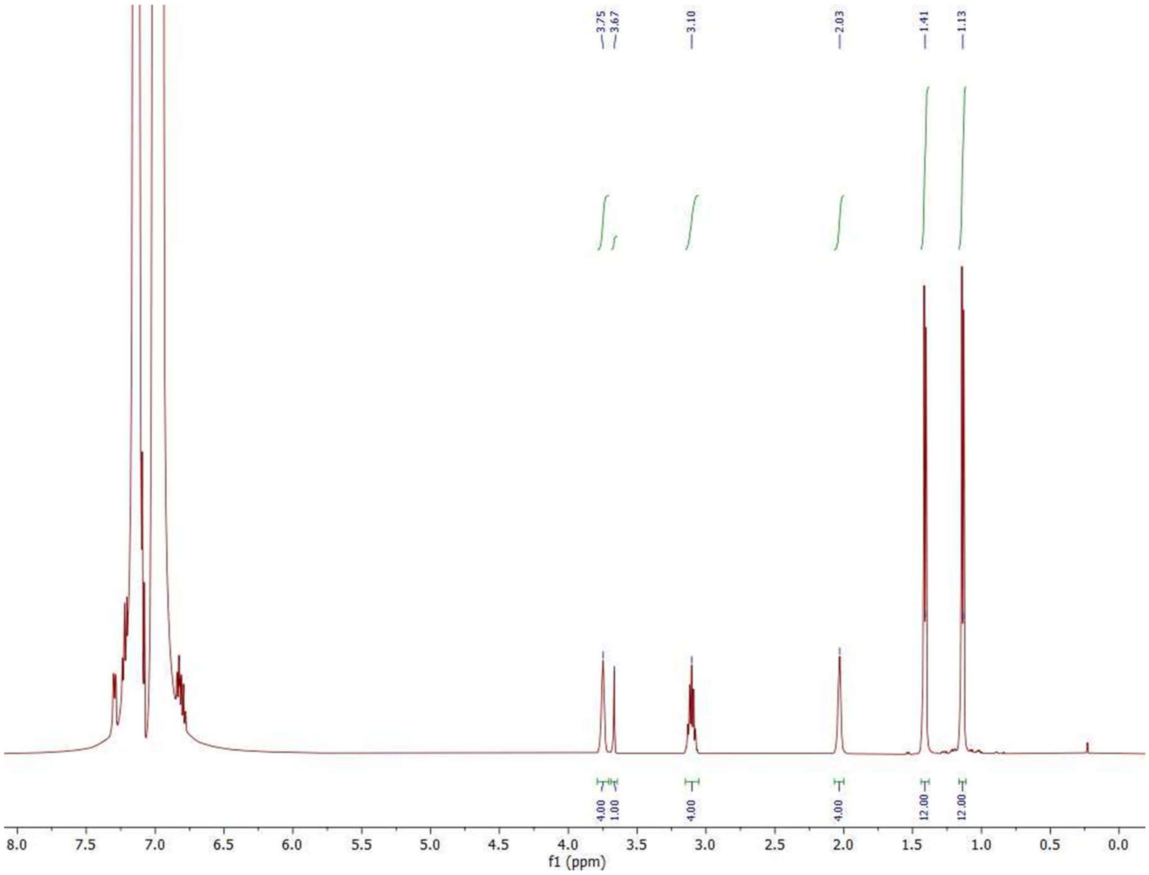

Figure S60 – ^{1}H NMR spectra of **1-H** in PhCl before addition of DBPin.

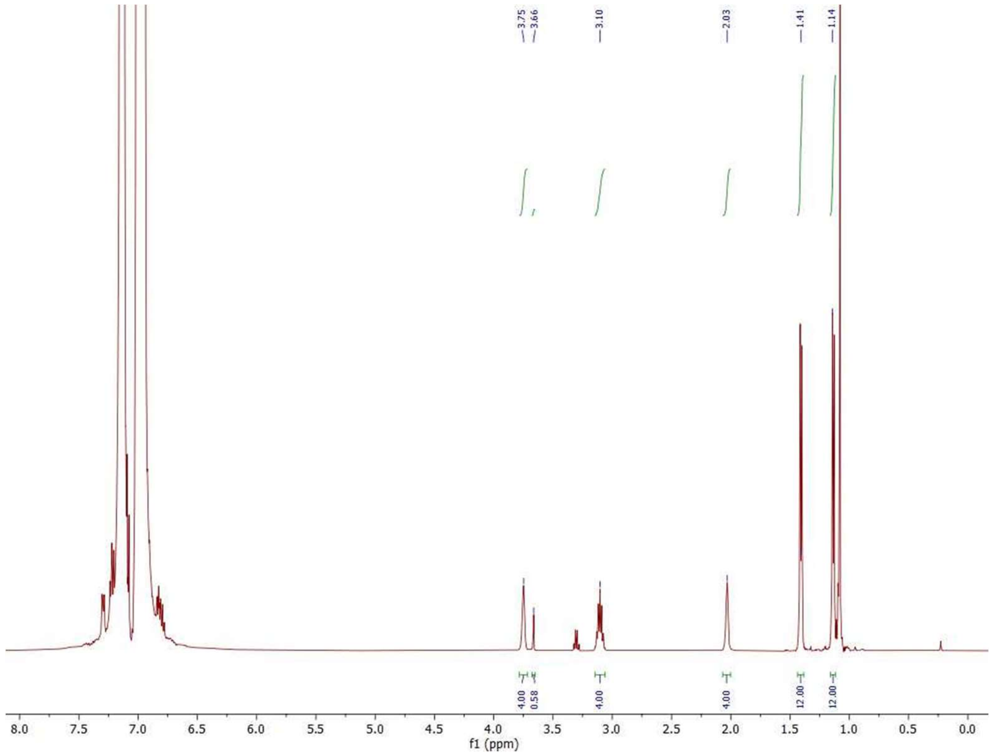

Figure S61 – ^{1}H NMR spectra of **1-H** in PhCl shortly after addition of DBPin.

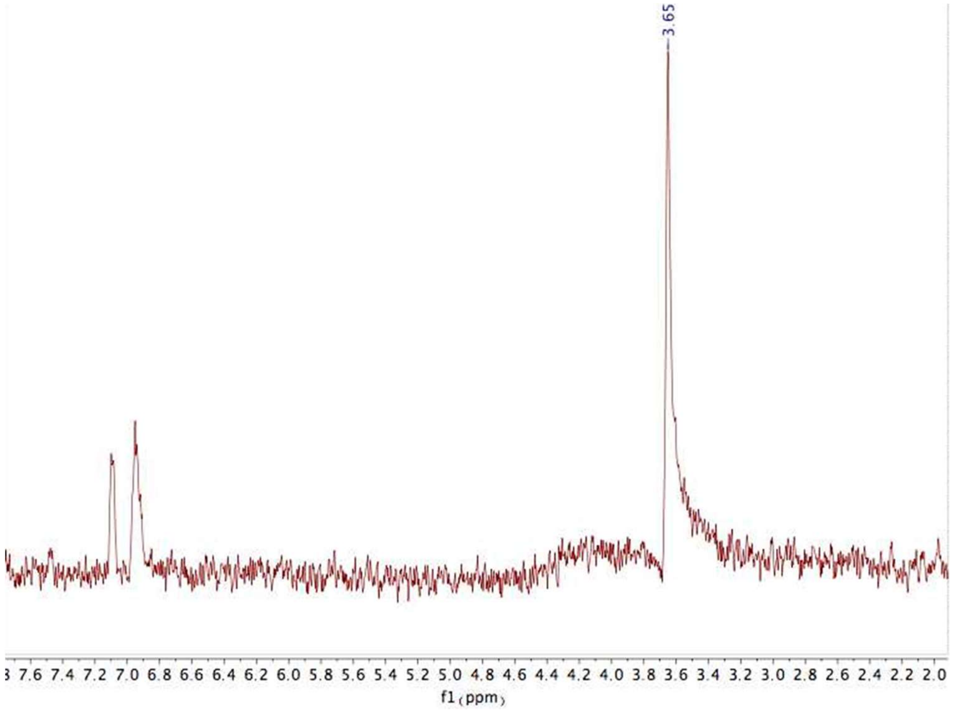

Figure S62 – ^{2}H NMR spectrum of **1-H** shortly after addition of DBPin in PhCl.

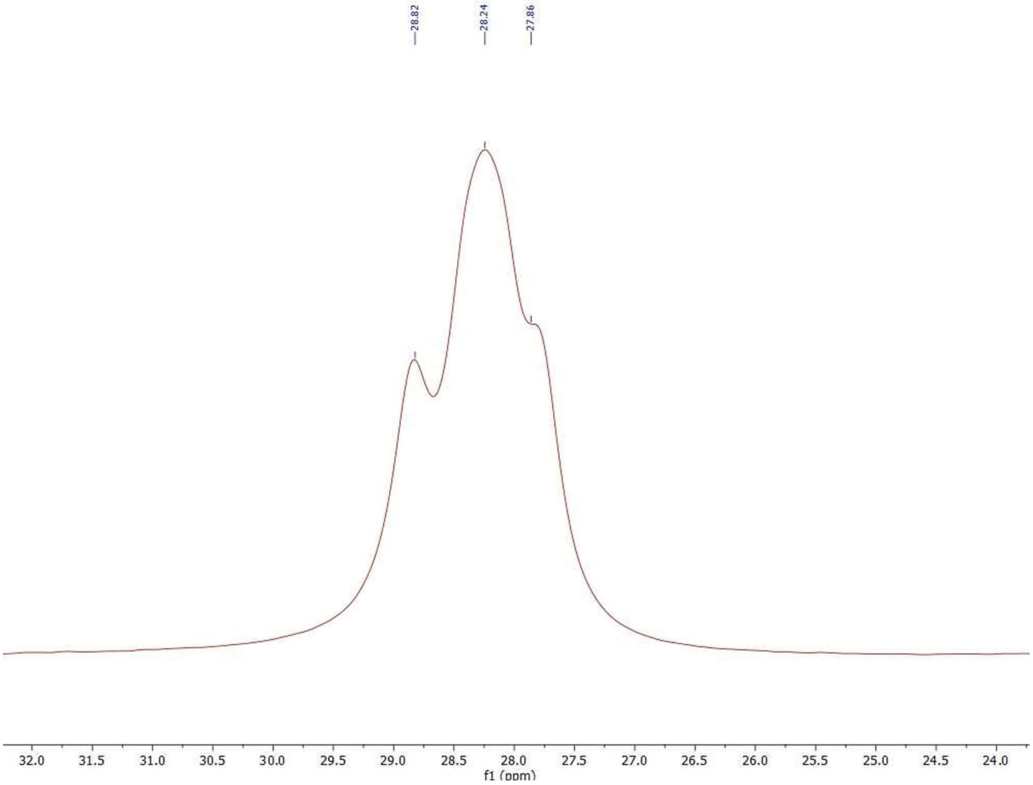

Figure S63 – ^{11}B NMR spectrum of **1-H** shortly after addition of DBPin in PhCl.

S9. Gutmann-Beckett Lewis Acidity tests

The electrophilicity of compounds **1-Ph** and **1-H** as well as the free diarylzinc precursor $ZnPh_2$ were measured by the Gutmann-Beckett method.[18-20] The $^{31}P\{^1H\}$ NMR spectrum of a 3:1 ratio of each compound with triethylphosphine oxide (Et_3PO) in DCM was measured (Table **S5**), data for $B(C_6F_5)_3$, [IDippZnMe][$B(C_6F_5)$] and **9** are as reported by Dagorne and co-workers (the measurements for $B(C_6F_5)_3$ and **9** were repeated as part of this study).

Table S5 - Upfield change in ^{31}P chemical shift of $Et_3\underline{P}O$ on addition of various electrophilic compounds.

	**$\Delta\delta^{31}P\{^1H\}$ / ppm**
**$ZnPh_2$**	12.2
**[IDippZnMe][$B(C_6F_5)_4$]**	17.2[a]
**7DippZnPhNTf$_2$ (1-Ph)**	19.7
**7DippZnHNTf$_2$ (1-H)**	22.0
**[IDippZnC_6F_5][$B(C_6F_5)_4$] (9)**	24.7
**$B(C_6F_5)_3$**	26.4

[a]: Data reported by Dagorne and co-workers.[6]

S10. Et_3PO-affinity competition experimentation

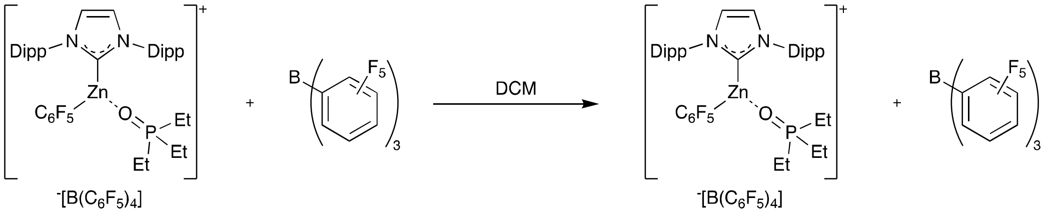

A J Youngs tube was charged with **9** (39 mg, 0.030 mmol, 3 eq.) and dissolved in DCM (500 μL) before addition of a 1 M solution of triethylphosphine oxide in DCM (10 μL, 0.010 mmol, 1 eq.). After analysis by ^{31}P NMR spectroscopy $B(C_6F_5)_3$ (15 mg, 0.030 mmol, 3eq.) was added, ^{31}P NMR spectroscopy was then used to analyse the reaction mixture immediately after addition of $B(C_6F_5)_3$ and again after a further 3 days.

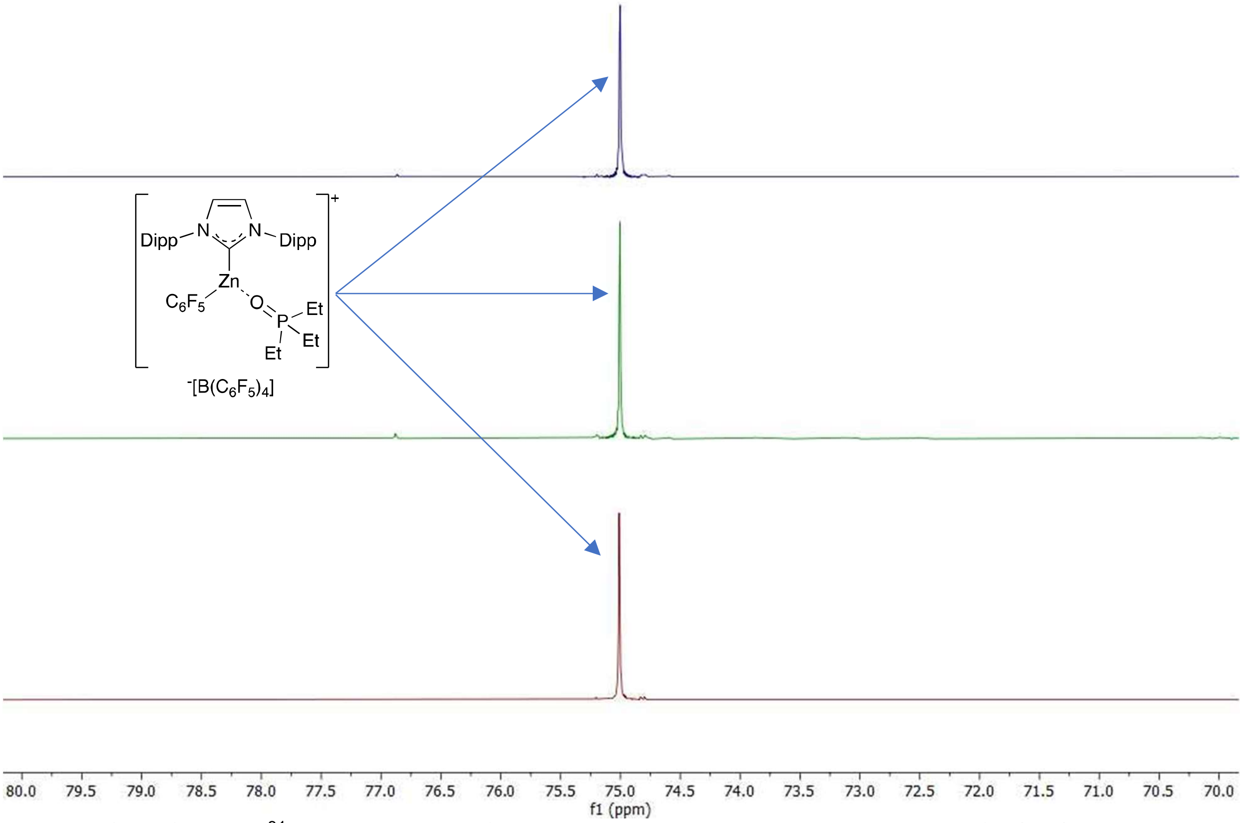

Figure S64: Stacked ^{31}P NMR spectra of triethylphosphine oxide. In the presence of **9** (red). Immediately after addition of $B(C_6F_5)_3$ (green). 3 days after addition of $B(C_6F_5)_3$ (blue).

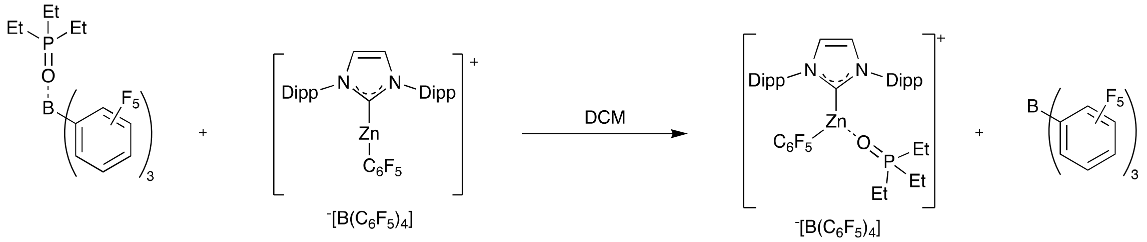

A J Youngs tube was charged with $B(C_6F_5)_3$ (15 mg, 0.030 mmol, 3eq.) and dissolved in DCM (500 μL) before addition of a 1 M solution of triethylphosphine oxide in DCM (10 μL, 0.010 mmol, 1 eq.). After analysis by ^{31}P NMR spectroscopy **9** (39 mg, 0.030 mmol, 3 eq.) was added, ^{31}P NMR spectroscopy was then used to analyse the reaction mixture immediately after addition of after $B(C_6F_5)_3$ and again after a further 3 days.

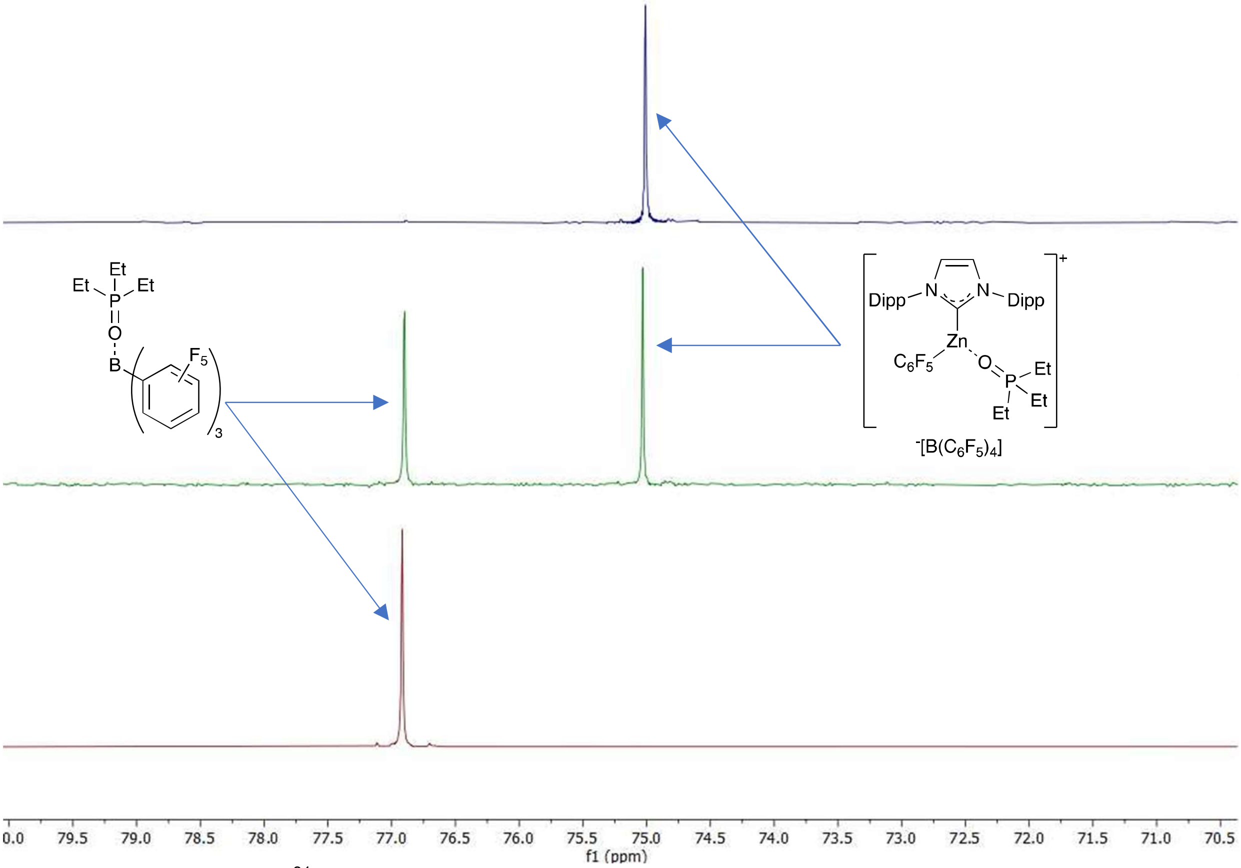

Figure S65: Stacked ^{31}P NMR spectra of triethylphosphine oxide. In the prescence of $B(C_6F_5)_3$ (red). Immediately after addition of **9** (green). 3 days after addition of **9** (blue).

S11. Hydride affinity competition experimentation

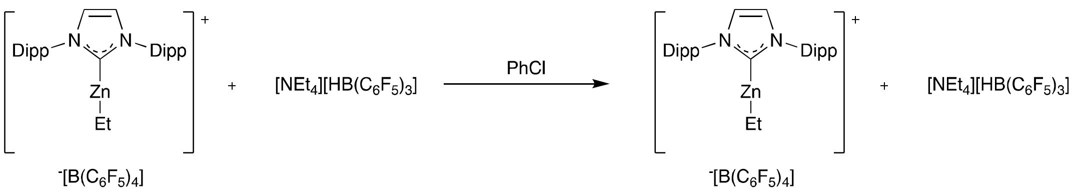

A J Youngs tube was charged with **5** (29 mg, 0.025 mmol, 1 eq.) and $[NEt_4][HB(C_6F_5)_3]$ (16 mg, 0.025 mmol, 1eq.), the solids were then dissolved in PhCl (500 μL). The reaction mixture was left at room temperature for 5 days, then heated to 80 °C for 18 hrs before analysis by ^1H and ^{11}B NMR spectroscopy.

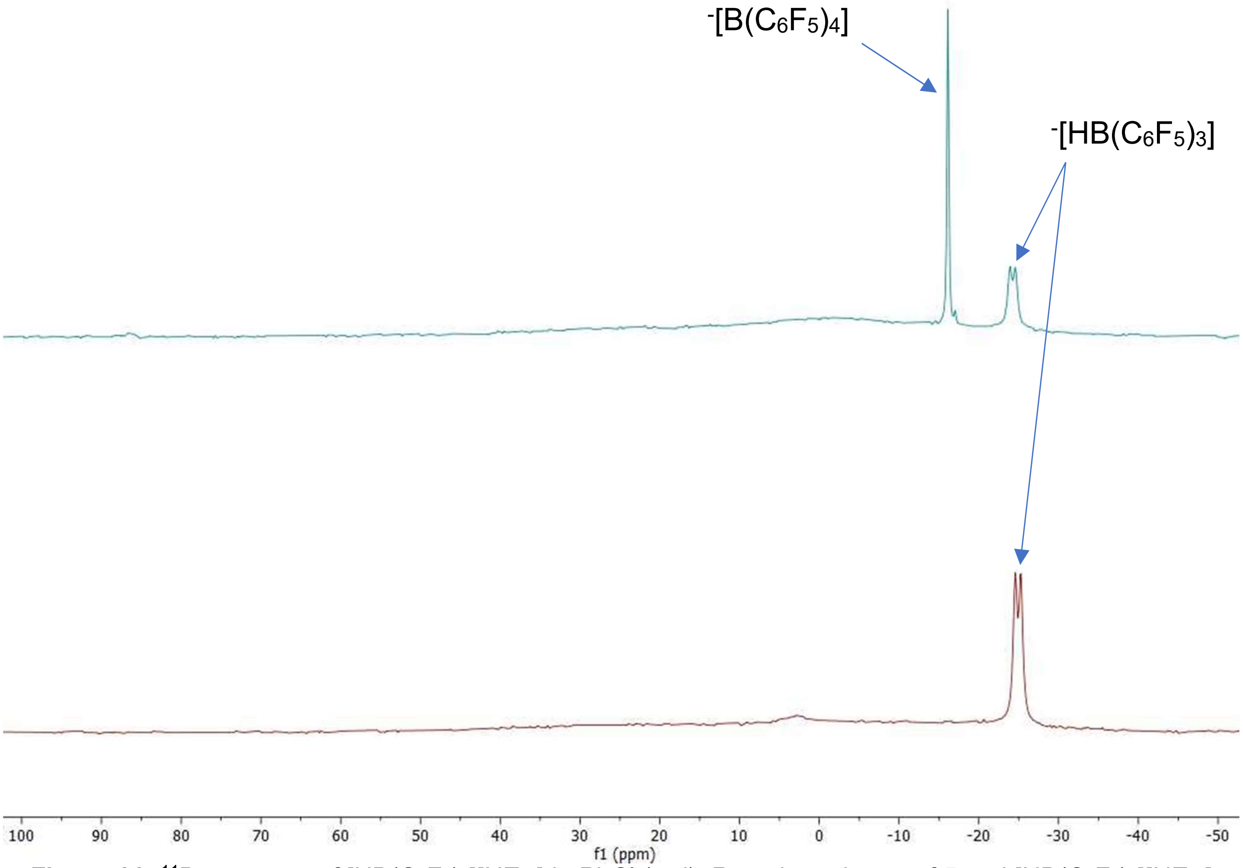

Figure 66: ^{11}B spectrum of $[HB(C_6F_5)_3][NEt_4]$ in PhCl (red). Reaction mixture of **5** and $[HB(C_6F_5)_3][NEt_4]$ after 18hrs at 80 °C (green).

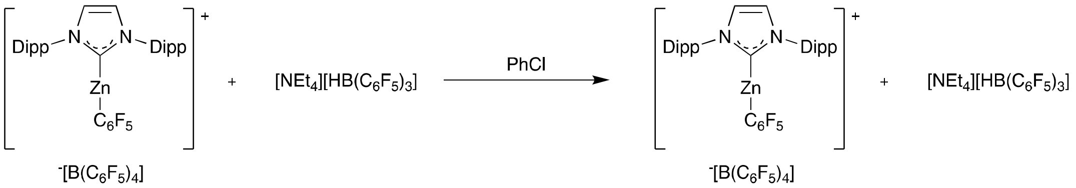

A J Youngs tube was charged with **9** (32 mg, 0.025 mmol, 1 eq.) and $[NEt_4][HB(C_6F_5)_3]$ (16 mg, 0.025mmol, 1eq.), the solids were then dissolved in PhCl (500 μL). The reaction mixture was left at room temperature for 5 days, then heated to 80 ºC for 18 hrs before analysis by ^1H and ^{11}B NMR spectroscopy.

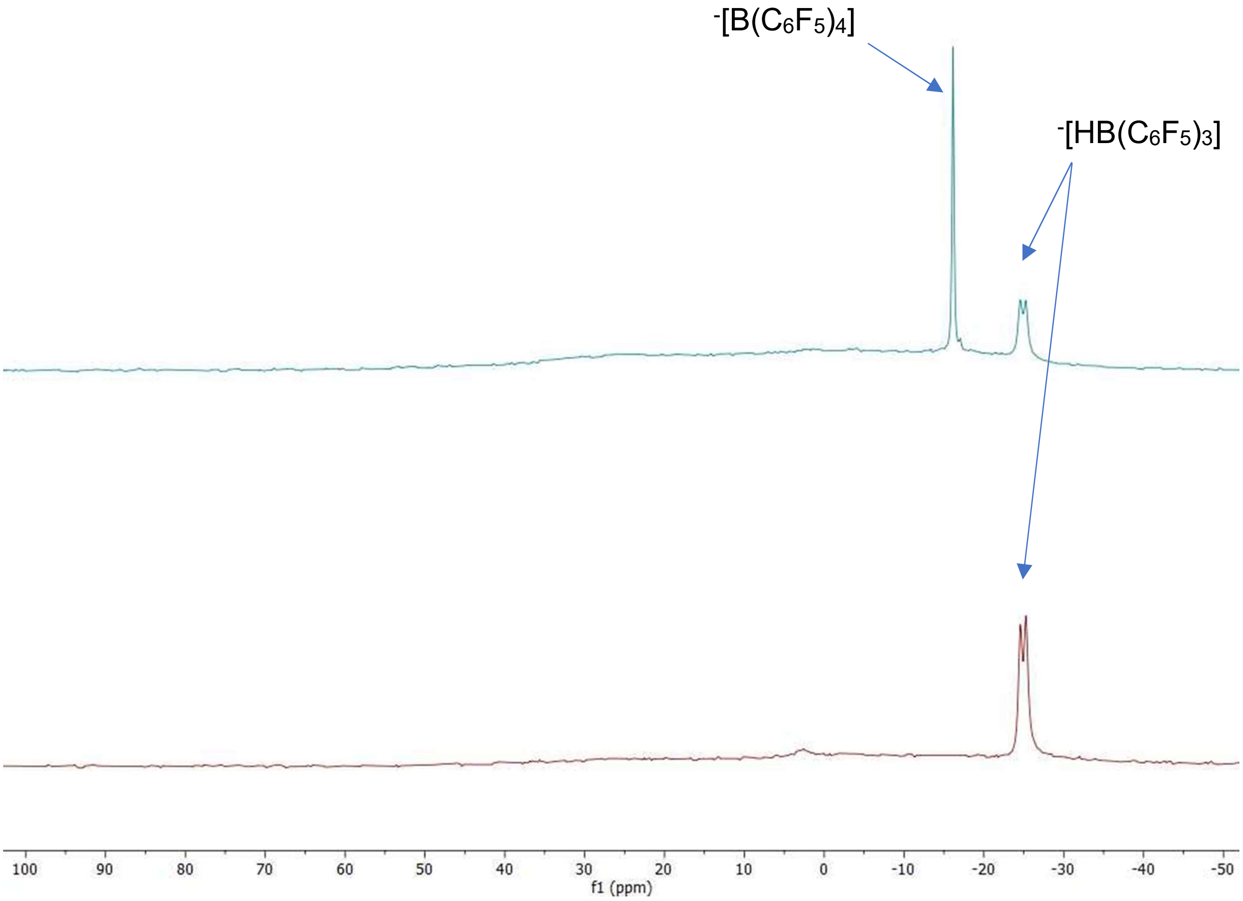

Figure 67: ^{11}B spectrum of $[HB(C_6F_5)_3][NEt_4]$ in PhCl (red). Reaction mixture of **9** and $[HB(C_6F_5)_3][NEt_4]$ after 18hrs at 80 °C (green).

S12. Isolation of borenium species [IDippBPin][NTf_2]

The use of IDippZnPhNTf_2 (in the presence of catalytic 2-dimethylaminopyridine) as a catalyst in the C-H borylation of **2a** was unsuccessful in achieving substrate conversion, however the complex [IDippBPin][NTf_2] (**8**) was isolated from this reaction mixture. A J Youngs tube was charged with IDippZnPh(NTf_2) (41 mg, 0.05 mmol) and dissolved in PhCl (500 μL), Pinacolborane (145 μL, 1.00 mmol, 2eq.) and 2-dimethylaminopyridine (6.2 μL, 0.05 mmol) were then added followed by N-Me-Indole (**2a**) (63 μL, 0.50 mmol, 1eq.) and the reaction mixture heated to 80 ºC for 18 hrs. Analysis by ^1H and ^{11}B NMR spectroscopy showed no borylation of **2a**. Storage of the reaction mixture at room temperature lead to the formation of crystalline material, analysis by single crystal X-ray crystallography showed the formation of NHC-borenium complex **8** (**Figure S68**).

S13. X-Ray Crystallography

S13.1 Crystal structure of [IDippBPin][NTf_2]

A suitable crystal with dimensions 0.39 × 0.12 × 0.03 mm^3 was selected and mounted on a Bruker D8 VENTURE diffractometer. The crystal was kept at a steady T = 100.0 K during data collection. The structure was solved and the space group $P2_1/c$ (# 14) determined by the ShelXT structure solution program using dual methods and refined by full matrix least squares minimisation on $\boldsymbol{F^2}$ using version 2018/3 of ShelXL 2018/3.[21-23] All non-hydrogen atoms were refined anisotropically. Hydrogen atom positions were calculated geometrically and refined using the riding model. The data set was truncated at 1 Å resolution during integration due to rapidly rising values of R(merge) at higher angle. Selected crystallographic data are presented in Table **S6** and full details of [IDippBPin][NTf_2] in cif format can be obtained free of charge from the Cambridge Crystallographic Data Centre via www.ccdc.cam.uk/data_request/cif.

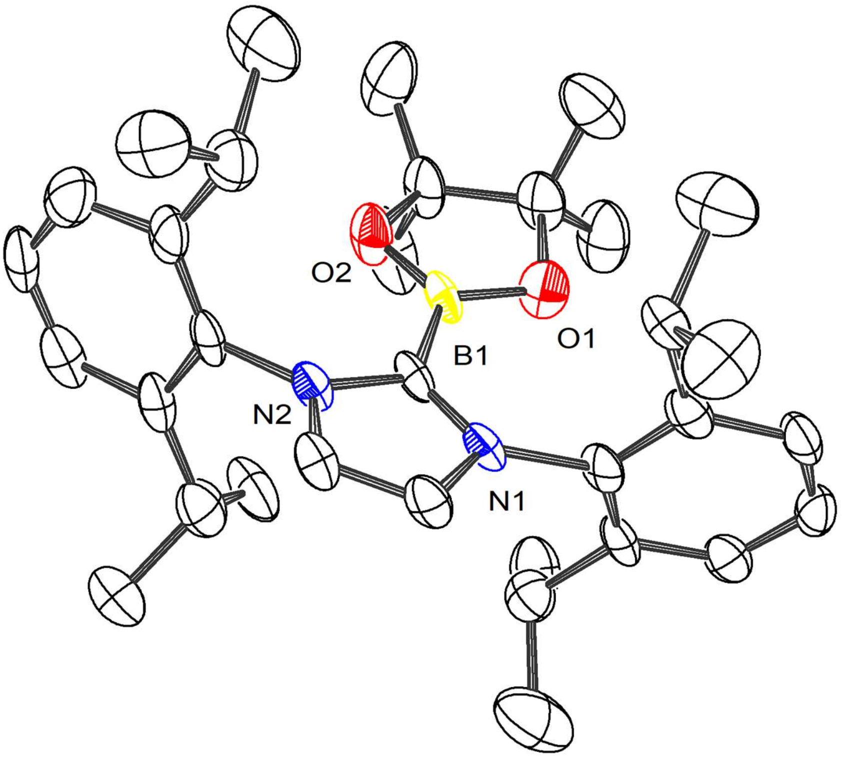

Figure S68: ORTEP plot for [IDippBPin][NTf_2] , counter anion and hydrogens are omitted for clarity (50% probability).

Table S6 – Crystal data and structure refinement for [IDippBPin][NTf_2].

**Identification code**	2074219
**Formula**	$C_{35}H_{48}BF_6N_3O_6S_2$
$D_{calc.}$**/ g cm^{-3}**	1.299
**μ/mm^{-1}**	0.203
**Formula Weight**	795.69
**Colour**	clear colourless
**Shape**	plate
**Size/mm^3**	0.39×0.12×0.03
***T*/K**	100.0
**Crystal System**	monoclinic
**Space Group**	$P2_1/c$
***a*/Å**	19.260(5)
***b*/Å**	12.118(3)
***c*/Å**	19.857(7)
**α/°**	90
**β/°**	118.607(5)
**γ/°**	90
**V/Å^3**	4069(2)
Z	4
Z'	1
**Wavelength/Å**	0.71073
**Radiation type**	MoK□
Θ_{min}**/°**	2.337
Θ_{max}**/°**	19.796
**Measured Refl's.**	37868
**Indep't Refl's**	3681
**Refl's I≥2 σ(I)**	2694
R_{int}	0.1146
**Parameters**	491
**Restraints**	0
**Largest Peak**	0.407
**Deepest Hole**	-0.257
**GooF**	1.097
wR_2 **(all data)**	0.1605
wR_2	0.1383
R_1 **(all data)**	0.0851

S13.2 Crystal structure of 11

A suitable crystal with dimensions 0.30 × 0.14 × 0.11 mm^3 was selected and mounted on a Xcalibur, Eos diffractometer. The crystal was kept at a steady *T* = 120.0 K during data collection. The structure was solved and the space group Pc determined by the ShelXT structure solution program using dual methods and refined by full matrix least squares minimisation on F^2 using version 2018/3 of ShelXL 2018/3.[21-23] All non-hydrogen atoms were refined anisotropically. Hydrogen atom positions were calculated geometrically and refined using the riding model. Selected crystallographic data are presented in Table **S7** and full details of **11** in cif format can be obtained free of charge from the Cambridge Crystallographic Data Centre via www.ccdc.cam.uk/data_request/cif.

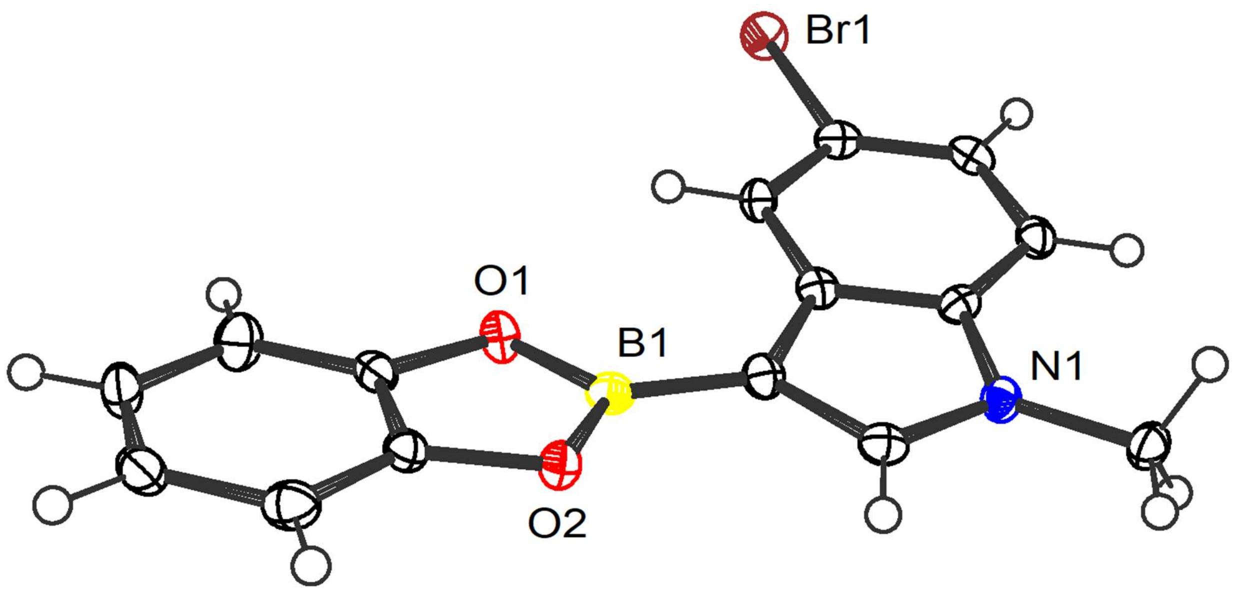

Figure S69: ORTEP plot of **11** (50% probability).

Table S7: Crystal data and structure refinement for **11**.

**Identification code**	2074218
**Empirical formula**	$C_{15}H_{11}BBrNO_2$
**Formula weight**	327.97
**Temperature/K**	119.99(11)
**Crystal system**	monoclinic
**Space group**	Pc
**a/Å**	11.8776(6)
**b/Å**	4.6452(2)
**c/Å**	12.9310(6)
**α/°**	90
**β/°**	113.246(6)
**γ/°**	90
**Volume/Å^3**	655.53(6)
**Z**	2
**ρ_{calc}g/cm^3**	1.662
**μ/mm^{-1}**	3.134
**F(000)**	328.0
**Crystal size/mm^3**	0.298 × 0.14 × 0.105
**Radiation**	Mo Kα (λ = 0.71073)
**2Θ range for data collection/°**	6.858 to 58.984
**Index ranges**	-14 ≤ h ≤ 15, -6 ≤ k ≤ 5, -16 ≤ l ≤ 17
**Reflections collected**	7683
**Independent reflections**	2641 [R_{int} = 0.0362, R_{sigma} = 0.0449]
**Data/restraints/parameters**	2641/2/182
**Goodness-of-fit on F^2**	1.041
**Final R indexes [I>=2σ (I)]**	R_1 = 0.0312, wR_2 = 0.0668
**Final R indexes [all data]**	R_1 = 0.0356, wR_2 = 0.0691
**Largest diff. peak/hole / e Å^{-3}**	0.43/-0.55
**Flack parameter**	-0.017(8)

S13.3 Crystal structure of 7DippZnEt$_2$

A suitable crystal with dimensions 0.45 × 0.25 × 0.14 mm^3 was selected and mounted on a Bruker D8 VENTURE diffractometer. The crystal was kept at a steady *T* = 120.0 K during data collection. The structure was solved and the space group P2$_1$ determined by the ShelXT structure solution program using dual methods and refined by full matrix least squares minimisation on ***F*²** using version 2018/3 of ShelXL 2018/3.[21-23] All non-hydrogen atoms were refined anisotropically. Hydrogen atom positions were calculated geometrically and refined using the riding model. Selected crystallographic data are presented in Table **S8** and full details of 7DippZnEt$_2$ in cif format can be obtained free of charge from the Cambridge Crystallographic Data Centre via www.ccdc.cam.uk/data_request/cif.

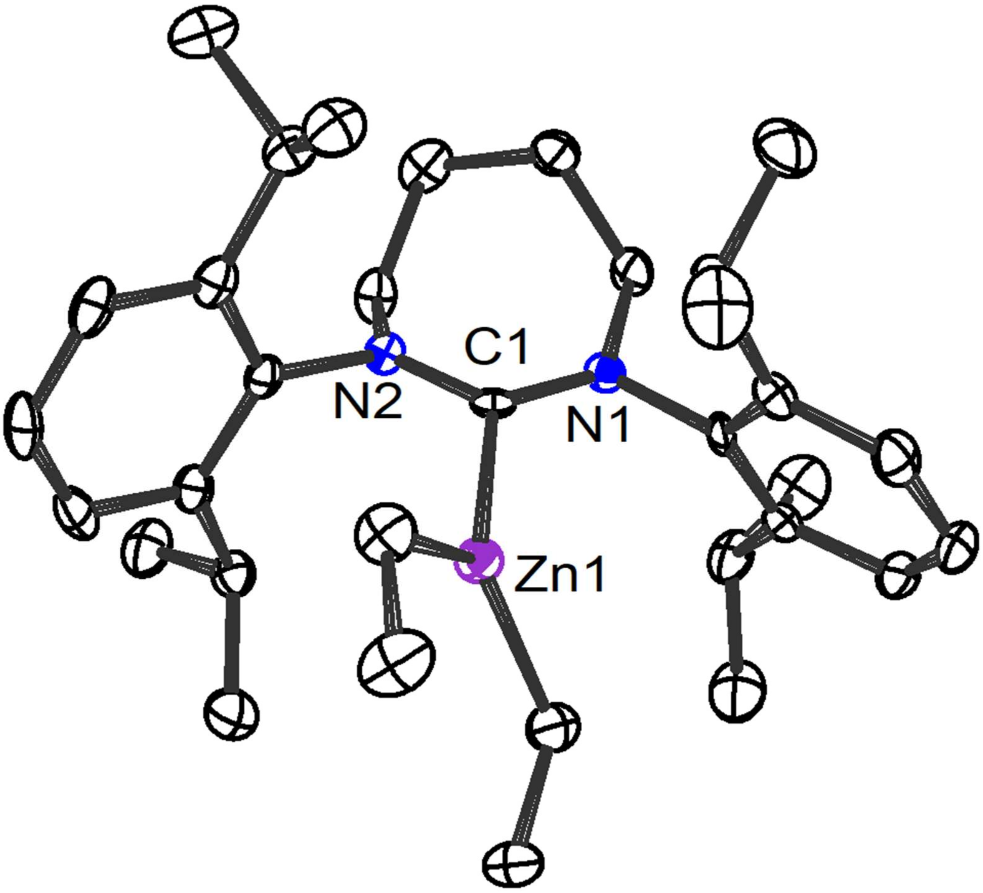

Figure S70: ORTEP plot of 7DippZnEt$_2$ (50% probability).

Table S8 - Crystal data and structure refinement for **7DippZnEt$_2$**.

**Identification code**	2074217
**Empirical formula**	$C_{33}H_{52}N_2Zn$
**Formula weight**	542.13
**Temperature/K**	120.0(5)
**Crystal system**	monoclinic
**Space group**	$P2_1$
**a/Å**	15.9444(3)
**b/Å**	19.6420(3)
**c/Å**	19.6889(3)
**α/°**	90
**β/°**	90.0090(10)
**γ/°**	90
**Volume/Å^3**	6166.17(18)
**Z**	8
**ρ_{calc}g/cm^3**	1.168
**μ/mm^{-1}**	0.819
**F(000)**	2352.0
**Crystal size/mm^3**	0.451 × 0.247 × 0.143
**Radiation**	MoKα (λ = 0.71073)
**2Θ range for data collection/°**	6.582 to 59.04
**Index ranges**	-21 ≤ h ≤ 21, -26 ≤ k ≤ 26, -25 ≤ l ≤ 25
**Reflections collected**	135687
**Independent reflections**	30273 [R_{int} = 0.0479, R_{sigma} = 0.0566]
**Data/restraints/parameters**	30273/34/1359
**Goodness-of-fit on F^2**	1.026
**Final R indexes [I>=2σ (I)]**	R_1 = 0.0389, wR_2 = 0.0647
**Final R indexes [all data]**	R_1 = 0.0474, wR_2 = 0.0675
**Largest diff. peak/hole / e Å^{-3}**	0.61/-0.51

S14. DFT calculations

S14.1 Experimental

All of the calculations were performed using the Gaussian09 series of programs.[24] For Et_3PO and hydride affinity calculations, geometry optimisations were carried out with three different functionals: B3PW91[25] , M06-2X[26] and B3LYP[25a,27]-D3BJ[28]. The rest of the optimisations were completed with the B3PW91 functional. Calculations were run with a 6-311G(d,p) (for H, B, C, N, O, F) basis set and a LANL2DZ (for Zn) basis set. All geometry optimizations were full, with no restrictions. Stationary points located in the potential energy surface were characterized as minima (no imaginary frequencies) by vibrational analysis. Solvent effects of chlorobenzene were introduced using the self consistent field approach, by means of the integral equation formalism polarizable continuum model (IEFPCM).[29]

S14.2 Energy profiles (Lewis acidity towards O=PEt_3)

B3PW91

	BCF	[DIPPZnC_6F_5-$OPEt_3$]$^+$	[DIPPZnC_6F_5]$^+$	BCF-$OPEt_3$	Δ (kcal/mol)
**E** (a.u.)	-2208.014054	-2607.38472	-1953.110479	-2862.287542	0.47
**H** (a.u.)	-2207.830083	-2606.502494	-1952.445131	-2861.885382	1.30
**G** (a.u.)	-2207.922291	-2606.650806	-1952.560523	-2862.002606	6.26

N.B. BCF = B(C_6F_5)$_3$

M06-2X

	BCF	[DIPPZnC_6F_5-$OPEt_3$]$^+$	[DIPPZnC_6F_5]$^+$	BCF-$OPEt_3$	Δ (kcal/mol)
**E** (a.u.)	-2208.161823	-2607.21323537	-1952.94101248	-2862.43853297	-2.82
**H** (a.u.)	-2207.975244	-2606.323785	-1952.27032100	-2862.031626	-1.83
**G** (a.u.)	-2208.064031	-2606.462424	-1952.38450000	-2862.143268	-0.82

B3LYP-D3BJ

	BCF	[DIPPZnC_6F_5-$OPEt_3$]$^+$	[DIPPZnC_6F_5]$^+$	BCF-$OPEt_3$	Δ (kcal/mol)
**E** (a.u.)	-2208.949068	-2608.536022	-1954.024284	-2863.457577	2.03
**H** (a.u.)	-2208.766029	-2607.65205	-1953.35803	-2863.055931	2.58
**G** (a.u.)	-2208.857431	-2607.792586	-1953.471727	-2863.171211	4.44

S14.2 Energy profiles (Hydride ion affinity)

B3PW91

	**BCF**	**DIPPZnC$_6$F$_5$(H)**	**[DIPPZnC$_6$F$_5$]$^+$**	**[HBCF]$^-$**	**Δ (kcal/mol)**
**E** (a.u.)	-2208.014054	-1953.82329790	-1953.1104791	-2208.765205	-24.05
**H** (a.u.)	-2207.830083	-1953.154888	-1952.445131	-2208.572601	-20.56
**G** (a.u.)	-2207.922291	-1953.279594	-1952.560523	-2208.665243	-14.99

N.B. [HBCF]$^-$ = [HB(C$_6$F$_5$)$_3$]$^-$

M06-2X

	**BCF**	**DIPPZnC$_6$F$_5$(H)**	**[DIPPZnC$_6$F$_5$]$^+$**	**[HBCF]$^-$**	**Δ (kcal/mol)**
**E** (a.u.)	-2208.161823	-1953.66754290	-1952.94101248	-2208.91374	-15.93
**H** (a.u.)	-2207.975244	-1952.993503	-1952.270321	-2208.718135	-12.37
**G** (a.u.)	-2208.064031	-1953.106644	-1952.3845	-2208.808863	-14.24

B3LYP-D3BJ

	**BCF**	**DIPPZnC$_6$F$_5$(H)**	**[DIPPZnC$_6$F$_5$]$^+$**	**[HBCF]$^-$**	**Δ (kcal/mol)**
**E** (a.u.)	-2208.949068	-1954.750477	-1954.024284	-2209.699723	-15.35
**H** (a.u.)	-2208.766029	-1954.081394	-1953.35803	-2209.508235	-11.82
**G** (a.u.)	-2208.857431	-1954.199564	-1953.471727	-2209.599746	-9.08

S14.3 Lowest energy unoccupied molecular orbitals with significant boron character

HBCat

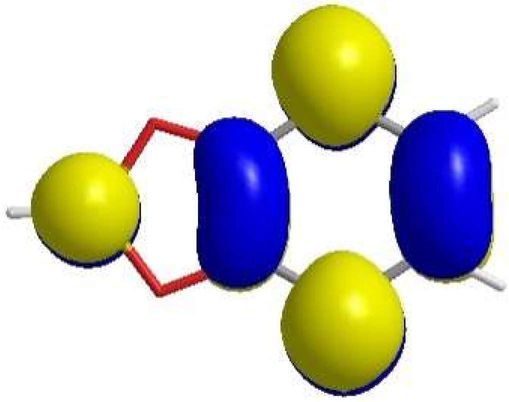

Figure S71: Representation of HBCat LUMO.

[DIPPZnH-HBCat]$^+$

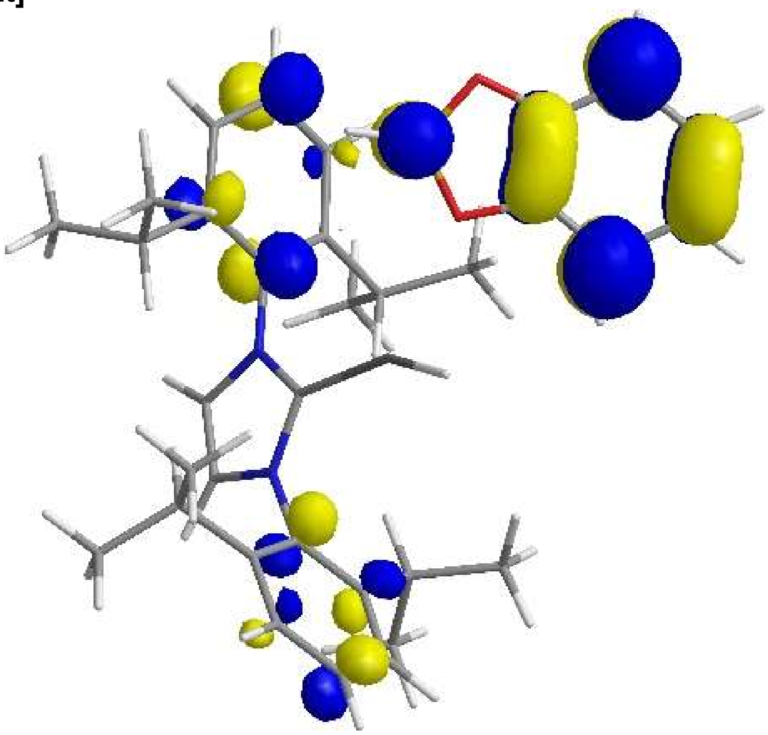

Figure S72: Representation of [DIPPZnH-HBCat]$^+$ LUMO+4.

HBPin

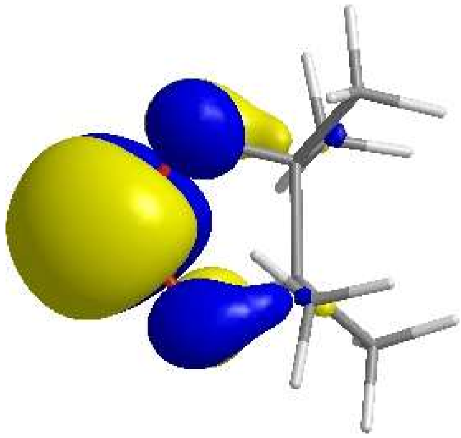

Figure S73: Representation of HBPin LUMO.

[DIPPZnH-HBPin]$^+$

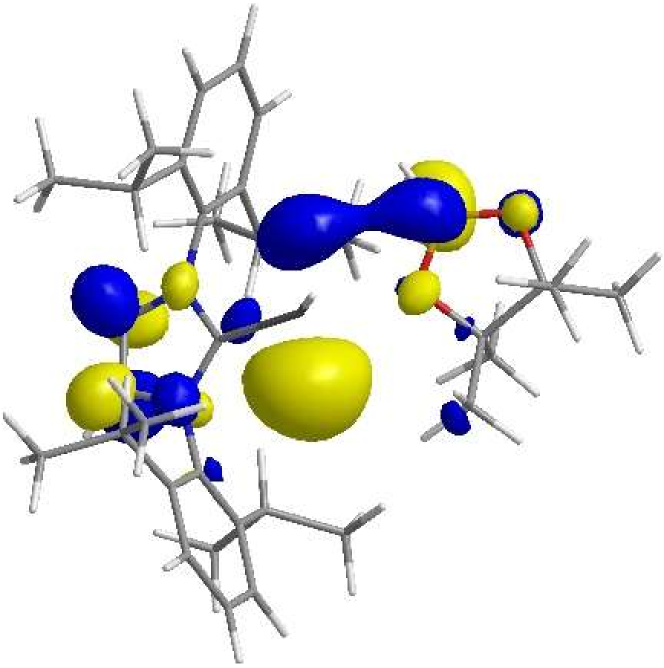

Figure S74: Representation of [DIPPZnH-HBPin]$^+$ LUMO.

S14.4 Coordinates of optimised structures

DIPPZnC6F5-B3PW91

Zn	3.1382090	6.5684810	9.0363540
N	3.2774630	7.1773140	5.9380310
N	2.9294820	8.9134030	7.1540770
C	3.1169350	7.5751710	7.2187400
C	3.5109520	5.8186860	5.5066940
C	2.4014030	5.0235320	5.1762530
C	1.4242680	10.0125250	8.7450510
C	3.1899990	8.2563200	5.0802280
H	3.2898220	8.1495690	4.0133790
C	2.9714250	9.3528910	5.8478280
H	2.8483490	10.3895570	5.5830310
C	2.7365600	9.7444590	8.3186470
C	0.9725790	5.5369070	5.2252250
H	0.9736130	6.5084790	5.7269100
C	6.0368540	6.2632140	5.6701110
H	5.6784620	7.1775290	6.1507540
C	4.8384430	5.3725670	5.3909770
C	0.1961180	9.5372010	7.9877700
H	0.5191120	8.8205340	7.2271190
C	3.8755850	10.2250250	8.9872770
C	1.2764020	10.7775150	9.9032750
H	0.2807770	11.0082380	10.2654100
C	2.6583200	3.7227840	4.7390130
H	1.8289490	3.0779330	4.4712320
C	3.6638200	10.9733810	10.1470030
H	4.5171810	11.3555710	10.6964110
C	5.0322680	4.0626560	4.9491540
H	6.0413540	3.6803050	4.8435410
C	0.0483840	4.6130090	6.0268760
H	-0.0702750	3.6401020	5.5423300
H	-0.9461730	5.0596920	6.1097500
H	0.4268350	4.4375050	7.0374790
C	3.9567280	3.2458250	4.6302430
H	4.1325520	2.2326280	4.2850230
C	5.2911690	9.9930640	8.4874100
H	5.2360790	9.4417530	7.5451980
C	2.3812680	11.2445390	10.6017030

H	2.2408730	11.8300900	11.5039900
C	-0.8079460	8.8177470	8.8960630
H	-0.3463250	7.9926770	9.4443160
H	-1.6264910	8.4103190	8.2965090
H	-1.2491920	9.4979010	9.6295960
C	7.0433440	5.6105190	6.6245000
H	6.5751030	5.3034140	7.5631440
H	7.8441930	6.3163290	6.8608040
H	7.5090570	4.7269650	6.1795970
C	0.4304220	5.7528930	3.8046190
H	1.0577040	6.4426480	3.2338220
H	-0.5813740	6.1662550	3.8435870
H	0.3872440	4.8077390	3.2554440
C	6.1084160	9.1452890	9.4691990
H	6.1924480	9.6318450	10.4451220
H	7.1207790	8.9920730	9.0856000
H	5.6589860	8.1607200	9.6268740
C	-0.4757970	10.7114790	7.2615380
H	-0.8383360	11.4550180	7.9774630
H	-1.3325290	10.3576090	6.6812390
H	0.2149730	11.2126380	6.5789460
C	6.7210090	6.6741720	4.3577970
H	7.1209680	5.8006110	3.8346280
H	7.5531770	7.3538620	4.5623030
H	6.0271930	7.1803450	3.6818170
C	5.9922930	11.3246740	8.1869880
H	5.4193730	11.9235260	7.4741110
H	6.9817460	11.1396560	7.7597440
H	6.1283910	11.9202840	9.0939840
C	3.1463900	5.6460860	10.8694520
C	1.9883840	5.2228520	11.4900870
C	4.3349280	5.3600140	11.5114170
C	1.9885890	4.5550130	12.7061920
C	4.3929720	4.6980540	12.7290130
C	3.2046960	4.2948610	13.3249940
F	0.7916770	5.4463400	10.9044170
F	0.8538710	4.1554690	13.2836340
F	3.2317820	3.6537640	14.4908350
F	5.5562070	4.4357560	13.3277870
F	5.5064070	5.7237920	10.9432640

DIPPZnC6F5H-B3PW91

Zn	0.3074560	-0.0165850	-1.6578350
N	2.3168920	0.0378280	0.8237300
N	0.4226860	0.7324630	1.5330680
C	1.0383270	0.2565650	0.4196280
C	3.3812130	-0.4655820	-0.0087340
C	4.2321090	0.4577600	-0.6378540
C	-1.3441170	2.3937840	1.1953970
C	2.4947080	0.3691610	2.1546930
H	3.4422390	0.2596910	2.6548400
C	1.2977300	0.8128160	2.6020390
H	0.9943730	1.1731630	3.5701960
C	-0.9661730	1.1087100	1.6198200
C	4.0658810	1.9610210	-0.4960440
H	3.1350050	2.1493300	0.0448930
C	2.6653840	-2.8577560	0.5969160
H	1.8352930	-2.3121740	1.0525260
C	3.5516070	-1.8547130	-0.1219680
C	-0.3332130	3.4195470	0.7102170
H	0.5646220	2.8820060	0.3931870
C	-1.8750290	0.1909810	2.1714700
C	-2.6938850	2.7324670	1.3016950
H	-3.0251760	3.7121460	0.9769990
C	5.2764950	-0.0527820	-1.4097590
H	5.9507890	0.6322410	-1.9125490
C	-3.2113500	0.5858280	2.2573380
H	-3.9428820	-0.0973160	2.6748390
C	4.6103200	-2.3091800	-0.9109930
H	4.7704240	-3.3760260	-1.0236610
C	3.9459000	2.6530460	-1.8590180
H	4.8685390	2.5601000	-2.4392470
H	3.7493780	3.7205350	-1.7215980
H	3.1312060	2.2239110	-2.4468620
C	5.4636650	-1.4205600	-1.5487020
H	6.2800860	-1.7961360	-2.1567850
C	-1.4482410	-1.1586530	2.7254800
H	-0.4229220	-1.3505100	2.3983670
C	-3.6192280	1.8375350	1.8202170
H	-4.6642190	2.1209390	1.8908070
C	-0.8251810	4.2292170	-0.4932000
H	-1.1775170	3.5802440	-1.2971360

H	-0.0105820	4.8469560	-0.8816900
H	-1.6413090	4.9053320	-0.2216120
C	2.0609100	-3.8873010	-0.3646510
H	1.5011400	-3.4063530	-1.1699050
H	1.3742180	-4.5459030	0.1747230
H	2.8323460	-4.5157130	-0.8189990
C	5.2109220	2.5639520	0.3292820
H	5.2780380	2.1058920	1.3198670
H	5.0586710	3.6392480	0.4622640
H	6.1735450	2.4213480	-0.1713370
C	-2.3115320	-2.3164120	2.2145190
H	-3.3538180	-2.2175970	2.5310100
H	-1.9368490	-3.2619470	2.6176020
H	-2.2892440	-2.3845410	1.1263690
C	0.0664320	4.3529640	1.8633140
H	-0.7993740	4.9218850	2.2164210
H	0.8273560	5.0657860	1.5314460
H	0.4711820	3.7967550	2.7129020
C	3.4386700	-3.5529940	1.7264190
H	4.2773160	-4.1328770	1.3285250
H	2.7828590	-4.2403830	2.2689510
H	3.8404870	-2.8305100	2.4419860
C	-1.4495570	-1.1254230	4.2615370
H	-0.8091480	-0.3301460	4.6518550
H	-1.0911770	-2.0777680	4.6635390
H	-2.4603840	-0.9566050	4.6453950
C	-1.7569530	-0.5420620	-1.8382330
C	-2.6986370	0.3672810	-2.2704410
C	-2.2348350	-1.8149180	-1.6157640
C	-4.0400960	0.0643350	-2.4593880
C	-3.5611970	-2.1868380	-1.7821890
C	-4.4712880	-1.2302990	-2.2082700
F	-2.3296230	1.6466090	-2.5429550
F	-4.9222510	0.9848770	-2.8750230
F	-5.7578530	-1.5548370	-2.3769300
F	-3.9818460	-3.4358510	-1.5328670
F	-1.3878350	-2.7937980	-1.1954910
H	1.3689300	0.2419950	-2.9136280

BCF-B3PW91

B	0.0001040	-0.0002740	0.0010290

C	1.2048280	-0.9984220	0.0010140
C	-1.4669590	-0.5443250	0.0008030
C	2.3476660	-0.7787720	-0.7717130
C	3.4172810	-1.6579390	-0.7932200
C	3.3749220	-2.7970050	-0.0008440
C	2.2643430	-3.0505280	0.7925610
C	1.2020600	-2.1625040	0.7731770
C	-1.8490690	-1.6421580	-0.7737800
C	-3.1454350	-2.1283750	-0.7949940
C	-4.1097070	-1.5233840	-0.0004490
C	-3.7729420	-0.4365220	0.7947930
C	-2.4726480	0.0391960	0.7748620
C	1.2708380	2.1217690	0.7737560
C	0.2620500	1.5422260	0.0006870
C	-0.4987250	2.4218670	-0.7730310
C	-0.2721560	3.7878020	-0.7943460
C	0.7345710	4.3207040	-0.0008470
C	1.5087140	3.4857490	0.7933260
F	2.2327810	-4.1383100	1.5586290
F	4.3952480	-3.6428900	-0.0018030
F	4.4796590	-1.4242640	-1.5599320
F	2.4303150	0.2970990	-1.5601890
F	0.1615550	-2.4436560	1.5632410
F	-3.4755100	-3.1634490	-1.5636420
F	-5.3525520	-1.9838400	-0.0010350
F	-4.6983780	0.1333750	1.5628670
F	-2.1947080	1.0796930	1.5662200
F	-0.9594190	-2.2501340	-1.5644130
F	2.0338730	1.3611040	1.5644230
F	2.4658210	4.0024450	1.5602120
F	0.9568300	5.6273110	-0.0015400
F	-1.0047400	4.5909870	-1.5620260
F	-1.4705860	1.9550960	-1.5627550

HBCF-B3PW91

B	-0.0514060	0.0650890	-0.7773790
C	-0.1967600	1.5831980	-0.1816030
C	1.4246610	-0.5938450	-0.5189490
C	-1.0506420	2.4858870	-0.8097130
C	-1.2076740	3.8061600	-0.4111490

C	-0.4867850	4.2786310	0.6733040
C	0.3700100	3.4183420	1.3393570
C	0.4921870	2.1040610	0.9076560
C	2.4806170	-0.2685200	-1.3656560
C	3.7538930	-0.8101710	-1.2549860
C	4.0167440	-1.7291770	-0.2523410
C	3.0022130	-2.0810510	0.6222310
C	1.7450100	-1.5097190	0.4765340
C	-2.0060560	-0.8298610	0.8506270
C	-1.2812500	-0.9194020	-0.3322280
C	-1.6997840	-1.9323200	-1.1905610
C	-2.7594530	-2.7865930	-0.9174310
C	-3.4568260	-2.6462020	0.2711220
C	-3.0748030	-1.6589210	1.1641450
F	1.0628410	3.8628780	2.3976400
F	-0.6216570	5.5472960	1.0756590
F	-2.0437990	4.6291140	-1.0601470
F	-1.7882520	2.0994060	-1.8673580
F	1.3239930	1.3288590	1.6289860
F	4.7299870	-0.4590410	-2.1043410
F	5.2361450	-2.2647910	-0.1271000
F	3.2496420	-2.9605970	1.6034180
F	0.8254850	-1.8839990	1.3859890
F	2.3041860	0.6192950	-2.3620580
F	-1.6821440	0.0832420	1.7855600
F	-3.7356460	-1.5231160	2.3227830
F	-4.4815920	-3.4577340	0.5554290
F	-3.1179240	-3.7421460	-1.7869050
F	-1.0701470	-2.1341350	-2.3632430
H	-0.1471060	0.1803120	-1.9768800

DIPPZnC6F5-OPEt3-B3PW91

Zn	3.3736520	6.4008600	8.8777360
N	3.2577180	7.2274760	5.9030800
N	2.9376160	8.9260340	7.1815880
C	3.1358490	7.5883360	7.1996560
C	3.4645450	5.8715160	5.4475420
C	2.3367340	5.0955770	5.1292650
C	1.4028930	10.0280530	8.7371060
C	3.1382450	8.3278540	5.0781160
H	3.2067610	8.2522920	4.0060700
C	2.9374430	9.3993380	5.8851990

H	2.7970010	10.4421840	5.6561140
C	2.7254170	9.7460510	8.3536180
C	0.9168640	5.6315430	5.1958470
H	0.9407480	6.6066510	5.6895330
C	5.9988260	6.2510270	5.6315930
H	5.6567620	7.2603680	5.8753270
C	4.7808720	5.3981060	5.3256160
C	0.1921220	9.5394270	7.9603400
H	0.5329290	8.8213580	7.2092400
C	3.8448090	10.2430100	9.0408730
C	1.2244320	10.8219140	9.8711330
H	0.2194400	11.0627930	10.1993380
C	2.5656990	3.7879630	4.6977640
H	1.7221600	3.1578940	4.4391670
C	3.6032500	11.0239530	10.1728890
H	4.4421050	11.4197200	10.7349090
C	4.9465910	4.0792780	4.8979110
H	5.9475770	3.6743030	4.7978920
C	-0.0098790	4.7300640	6.0202110
H	-0.1448540	3.7515400	5.5515900
H	-0.9980340	5.1903450	6.1054480
H	0.3745270	4.5661970	7.0305170
C	3.8536380	3.2823280	4.5896420
H	4.0065690	2.2619370	4.2548890
C	5.2734510	9.9796850	8.6004070
H	5.2446820	9.3697830	7.6935530
C	2.3100000	11.3093990	10.5852070
H	2.1463920	11.9214330	11.4657250
C	-0.8180170	8.8143990	8.8571420
H	-0.3552050	7.9906100	9.4064440
H	-1.6290670	8.4044380	8.2490810
H	-1.2682230	9.4915190	9.5879790
C	6.7565650	5.7123630	6.8512210
H	6.1100830	5.6608630	7.7322080
H	7.6047600	6.3588240	7.0923780
H	7.1446530	4.7064810	6.6673270
C	0.3551480	5.8437380	3.7823300
H	0.9858310	6.5153190	3.1939590
H	-0.6476770	6.2770090	3.8341490
H	0.2839820	4.8942630	3.2435640
C	6.0410930	9.1902710	9.6662360

H	6.1455550	9.7650260	10.5906280
H	7.0459220	8.9440830	9.3122680
H	5.5293630	8.2567730	9.9159190
C	-0.4775510	10.7025680	7.2141710
H	-0.8557060	11.4512160	7.9165080
H	-1.3232600	10.3373580	6.6247530
H	0.2190590	11.2021310	6.5358590
C	6.9228020	6.3706880	4.4131660
H	7.3455480	5.4018950	4.1327630
H	7.7556680	7.0426690	4.6382120
H	6.3893560	6.7687100	3.5460900
C	5.9971000	11.2844110	8.2443460
H	5.4649540	11.8345930	7.4638820
H	7.0058410	11.0680200	7.8815340
H	6.0904000	11.9391730	9.1153050
C	3.4069810	5.4877500	10.7156110
C	2.2400550	5.0887720	11.3357360
C	4.5859300	5.2347200	11.3874360
C	2.2219260	4.4717410	12.5780100
C	4.6257550	4.6227450	12.6317850
C	3.4290250	4.2411100	13.2254750
F	1.0517910	5.2915660	10.7242340
F	1.0788180	4.0965050	13.1554100
F	3.4393150	3.6503510	14.4180240
F	5.7794870	4.3916150	13.2611390
F	5.7632690	5.5872800	10.8260210

BCF-OPEt3-B3PW91

B	0.0664290	0.1343030	0.1366330
C	1.5913460	0.6315920	-0.2296820
C	2.0602210	1.9342070	-0.0839780
C	2.5645540	-0.2822450	-0.6236180
C	3.3704560	2.3132470	-0.3552880
C	3.8798100	0.0473300	-0.9055380
C	4.2872980	1.3654950	-0.7745130
C	-1.0161540	1.3709240	0.2409560
C	-1.2615440	2.1386400	-0.8958470
C	-1.7962390	1.7102390	1.3395570
C	-2.1899480	3.1639020	-0.9593570
C	-2.7413570	2.7309330	1.3209080
C	-2.9424100	3.4639300	0.1654720
C	-0.6135120	-0.9082670	-0.9539250

C	-0.2310860	-1.0869500	-2.2819280
C	-1.7753050	-1.5912820	-0.6029190
C	-0.9135120	-1.9074750	-3.1723140
C	-2.4847280	-2.4242000	-1.4537590
C	-2.0453790	-2.5878850	-2.7569590
F	1.2589600	2.9220880	0.3465660
F	3.7539060	3.5842560	-0.2052950
F	5.5475300	1.7112100	-1.0338590
F	4.7572080	-0.8884530	-1.2798160
F	2.2554030	-1.5914530	-0.7315240
F	-2.2811390	-1.4621720	0.6427340
F	-3.5754640	-3.0684080	-1.0279690
F	-2.7041340	-3.3834300	-3.5979540
F	-0.4872040	-2.0435190	-4.4307470
F	0.8341810	-0.4547700	-2.7938360
F	-0.5535910	1.9081680	-2.0165740
F	-1.6862680	1.0635020	2.5131560
F	-3.4606900	3.0069920	2.4143760
F	-3.8450950	4.4450980	0.1335280
F	-2.3646280	3.8641990	-2.0841660
P	0.4163580	-1.7799990	2.4020660
O	0.2059250	-0.5279860	1.5140380
C	0.5393460	-3.3346510	1.4430450
H	1.4239550	-3.8515550	1.8276440
H	0.7812830	-3.0302030	0.4224260
C	1.9517390	-1.5963040	3.3536610
H	1.8314390	-0.6902100	3.9545590
H	1.9594570	-2.4413410	4.0528570
C	-0.9279060	-1.9235720	3.6176380
H	-0.9113600	-2.9594060	3.9720770
H	-1.8545880	-1.7930870	3.0542490
C	3.2473780	-1.5342550	2.5499320
H	3.3798940	-2.4026430	1.9009800
H	3.2819900	-0.6357930	1.9332180
H	4.0966710	-1.5002600	3.2355630
C	-0.8549050	-0.9539960	4.7979570
H	-1.7615110	-1.0538300	5.3987420
H	-0.0046990	-1.1719130	5.4473370
H	-0.7878740	0.0827060	4.4683960
C	-0.6656930	-4.2766540	1.4665800
H	-1.5812700	-3.7933430	1.1258250

H	-0.4677200	-5.1202310	0.8017070
H	-0.8461820	-4.6825210	2.4639220

HBPin-B3PW91

C	-0.7843830	-0.1908260	-0.0480530
C	0.7844240	-0.1907320	0.0480570
B	-0.0001120	1.9406030	-0.0000290
H	-0.0001690	3.1299830	-0.0001030
C	-1.4723370	-0.4639680	1.2863100
H	-2.5394360	-0.2536630	1.1830620
H	-1.3553340	-1.5070040	1.5898950
H	-1.0752520	0.1762600	2.0777140
C	1.3627630	-1.0819600	1.1337540
H	1.1145850	-2.1303130	0.9458540
H	2.4514590	-0.9888660	1.1397030
H	0.9924170	-0.8068210	2.1218760
C	1.4723990	-0.4638810	-1.2862930
H	2.5394720	-0.2534390	-1.1830690
H	1.3555210	-1.5069520	-1.5898070
H	1.0752270	0.1762450	-2.0777370
C	-1.3626120	-1.0821840	-1.1337010
H	-1.1143250	-2.1305000	-0.9457330
H	-2.4513180	-0.9892060	-1.1396700
H	-0.9922830	-0.8070670	-2.1218350
O	1.0759540	1.1994170	0.3861650
O	-1.0760670	1.1992690	-0.3862470

DIPPZnH-HBCat-H-bound-B3PW91

Zn	2.2374310	5.9291960	9.4260120
N	3.2750880	7.1531790	6.8116530
N	2.6987640	8.6922350	8.2015250
C	2.7852440	7.3527790	8.0536810
C	3.5319090	5.8595490	6.2211300
C	2.5059870	5.2439540	5.4834880
C	0.8435640	9.6655870	9.4688940
C	3.4973220	8.3611410	6.1827970
H	3.8858060	8.4221470	5.1803810
C	3.1336210	9.3312890	7.0583460
H	3.1439960	10.4042220	6.9679970
C	2.2127610	9.3604220	9.3879050

C	1.1515680	5.8916280	5.2483640
C	5.9398550	6.0043340	7.1086990
H	5.5136610	6.8392540	7.6724740
C	4.8107640	5.2973160	6.3782830
C	-0.1439860	9.3558630	8.3568990
H	0.3644960	8.7467120	7.6046070
C	3.1377420	9.7005290	10.3900900
C	0.4051460	10.3176660	10.6228810
H	-0.6443160	10.5707540	10.7248710
C	2.7912830	4.0023500	4.9122610
H	2.0278760	3.4935720	4.3344880
C	2.6390520	10.3520980	11.5193760
H	3.3194750	10.6338750	12.3148240
C	5.0357120	4.0555960	5.7818560
H	6.0088380	3.5874930	5.8773060
C	4.0386050	3.4134140	5.0614540
H	4.2379620	2.4492010	4.6063450
C	4.6291250	9.4387220	10.2624990
H	4.7884610	8.7742540	9.4082370
C	1.2897130	10.6541340	11.6373460
H	0.9259900	11.1622390	12.5239510
H	3.0700900	4.7011640	10.6625690
H	1.0049800	4.9729910	10.3392140
B	1.9240950	4.3091880	11.0306660
O	1.7414490	2.8931030	10.7812650
O	1.7025310	4.5696360	12.4396940
C	1.3308980	2.3775070	11.9749950
C	1.3073880	3.3726070	12.9589960
C	0.9810100	1.0732850	12.2609030
H	1.0049190	0.3086100	11.4927590
C	0.9327740	3.0929480	14.2577170
C	0.5989940	0.7811380	13.5786720
H	0.3184590	-0.2347930	13.8347910
C	0.5751900	1.7697560	14.5560120
H	0.2762310	1.5183220	15.5678590
H	0.9199490	3.8699560	15.0136710
H	1.0690750	6.7622350	5.9054590
C	6.9418350	6.5907350	6.1024030
H	6.4567040	7.2678810	5.3947610
H	7.7228910	7.1489210	6.6262450
H	7.4242220	5.7948990	5.5272040

C	6.6557350	5.0941460	8.1130560
H	7.1911530	4.2818090	7.6143860
H	7.3940070	5.6711070	8.6765940
H	5.9600520	4.6447670	8.8264770
C	5.3804350	10.7471300	9.9764590
H	4.9975130	11.2457290	9.0825170
H	5.2834650	11.4429340	10.8150290
H	6.4451110	10.5475590	9.8261280
C	5.2110480	8.7378180	11.4952050
H	5.1569370	9.3705940	12.3850710
H	4.6888700	7.8037380	11.7186330
H	6.2655620	8.5034040	11.3263410
C	-1.3478150	8.5492840	8.8585690
H	-1.9474600	9.1199100	9.5726750
H	-1.9988130	8.2875350	8.0199540
H	-1.0391140	7.6223580	9.3496020
C	-0.6017110	10.6484660	7.6658660
H	0.2452680	11.2171260	7.2733290
H	-1.2709560	10.4158160	6.8328390
H	-1.1444090	11.2942950	8.3622410
C	-0.0155140	4.9559990	5.5836560
H	-0.9632960	5.4901360	5.4746570
H	-0.0497000	4.0930030	4.9133550
H	0.0442990	4.5789230	6.6080680
C	1.0447090	6.3969460	3.8019150
H	1.8459470	7.0998880	3.5598270
H	1.1032130	5.5657940	3.0931280
H	0.0884120	6.9045530	3.6476580

DIPPZnH-HBcat-O-bound- B3PW91

C	-4.5402180	1.7743630	0.3075510
C	-4.3097900	2.7455120	-0.6076340
C	-2.5289360	1.3616460	-0.6222240
C	-3.3373860	-0.2454170	1.1055800
C	-2.4846300	3.2764200	-2.2137090
C	-2.7647870	2.9414520	-3.5493960
C	-2.2033580	3.7480170	-4.5405170
C	-1.4085140	4.8377770	-4.2150920
C	-1.1581070	5.1483830	-2.8861730
C	-1.6921940	4.3780680	-1.8516250
C	-3.9147490	-1.4370120	0.6360420

C	-3.8478640	-2.5527280	1.4729240
C	-3.2379170	-2.4808830	2.7167270
C	-2.6887310	-1.2849590	3.1582420
C	-2.7343630	-0.1338390	2.3695270
C	-2.2118170	1.1825930	2.9188050
C	-4.6169160	-1.5480280	-0.7071610
C	-3.9555540	-2.5962260	-1.6100580
C	-6.1108790	-1.8468260	-0.5224390
C	-1.4232520	4.7648960	-0.4078310
C	0.0781460	4.8412260	-0.1063050
C	-2.1196860	6.0873230	-0.0568310
C	-3.6583040	1.7775480	-3.9422960
C	-4.9757140	2.2814190	-4.5483180
C	-2.9526240	0.8073650	-4.8973360
C	-3.1709430	1.7341350	3.9843230
C	-0.7897920	1.0675640	3.4783710
Zn	-0.6471400	0.6026670	-1.2324050
C	1.9230910	-2.2449400	1.6220010
C	1.6631120	-1.0209320	1.0199510
C	2.5682580	0.0193720	1.0488040
C	3.7685190	-0.2232180	1.7230490
C	4.0291200	-1.4513480	2.3313220
C	3.1027250	-2.4961310	2.2918740
H	-5.3777340	1.6022510	0.9621450
H	-4.9043430	3.5912350	-0.9088560
H	-2.3959690	3.5208480	-5.5832720
H	-0.9843590	5.4513390	-5.0027200
H	-0.5399840	6.0064830	-2.6461780
H	-4.2824960	-3.4909730	1.1455320
H	-3.1966740	-3.3602840	3.3505180
H	-2.2325300	-1.2397030	4.1409040
H	-2.1815840	1.9048040	2.0984060
H	-4.5384810	-0.5842680	-1.2172430
H	-4.4530760	-2.6227970	-2.5835200
H	-2.8981300	-2.3740900	-1.7768470
H	-4.0218780	-3.5981190	-1.1771330
H	-6.6149720	-1.8672090	-1.4928090
H	-6.5975290	-1.0896080	0.0977730
H	-6.2641430	-2.8193980	-0.0458990
H	-1.8468370	3.9908570	0.2376690
H	0.2381730	5.0460400	0.9559960

H	0.5874000	3.9066900	-0.3549990
H	0.5619190	5.6421310	-0.6721930
H	-1.9596330	6.3319560	0.9970710
H	-3.1970920	6.0340960	-0.2337780
H	-1.7230120	6.9120900	-0.6560660
H	-3.9092540	1.2177370	-3.0372920
H	-5.6322180	1.4382870	-4.7818120
H	-5.5059800	2.9448270	-3.8600900
H	-4.7969210	2.8335930	-5.4756920
H	-3.5975750	-0.0513620	-5.1035970
H	-2.0147730	0.4356450	-4.4761880
H	-2.7214740	1.2814730	-5.8552120
H	-2.8210240	2.7064340	4.3428740
H	-4.1829410	1.8609510	3.5912860
H	-3.2273380	1.0588300	4.8432860
H	-0.7505800	0.4199950	4.3586750
H	-0.4290000	2.0534860	3.7845710
H	-0.0952220	0.6660650	2.7370190
H	0.6565670	0.5725690	-2.1833660
H	2.3722350	0.9713880	0.5726880
H	4.5125020	0.5636450	1.7702290
H	4.9719070	-1.6012350	2.8448760
H	3.2942700	-3.4548080	2.7579820
H	-1.1022040	-2.7690660	0.3255340
N	-3.4419300	0.9367800	0.2832630
N	-3.0765990	2.4764600	-1.1659760
B	-0.0634950	-2.3629490	0.7022040
O	0.4038730	-1.0767950	0.4406860
O	0.8512070	-3.0872100	1.4262540

DIPPZnC6F5-M062X

Zn	3.1859940	6.4178490	8.8187410
N	3.1041780	7.2542420	5.8413230
N	2.8002960	8.9353110	7.1291250
C	2.9921970	7.6034360	7.1334170
C	3.4136230	5.9118730	5.4190060
C	2.3758170	5.1014030	4.9453230
C	1.4867680	9.9125230	8.9454400
C	2.9863300	8.3580360	5.0215230
H	3.0526970	8.2837820	3.9490210
C	2.7897020	9.4247570	5.8392030

H	2.6610740	10.4724540	5.6251830
C	2.7366700	9.7123810	8.3439730
C	0.9387700	5.5824240	4.8500300
H	0.8914570	6.6137150	5.2076420
C	5.8735200	6.3892900	6.0022700
H	5.4554710	7.3511720	6.3099000
C	4.7440730	5.4860880	5.5305660
C	0.2005970	9.4439550	8.2880890
H	0.4409770	8.6144990	7.6164230
C	3.9432390	10.1759370	8.8818020
C	1.4722270	10.6170310	10.1483640
H	0.5301500	10.8013740	10.6497170
C	2.7101150	3.8016950	4.5650260
H	1.9374150	3.1392210	4.1921600
C	3.8713070	10.8706400	10.0901290
H	4.7810660	11.2445670	10.5454550
C	5.0212770	4.1753150	5.1404010
H	6.0376460	3.8042600	5.2042410
C	0.0156920	4.7414820	5.7400370
H	0.0008560	3.6979150	5.4165300
H	-1.0057090	5.1238350	5.6862310
H	0.3357420	4.7664040	6.7845580
C	4.0172480	3.3437610	4.6624570
H	4.2560220	2.3302360	4.3632890
C	5.2859320	9.9503350	8.2070780
H	5.1140970	9.4928420	7.2300700
C	2.6512540	11.0875110	10.7147740
H	2.6165110	11.6304570	11.6515600
C	-0.8330880	8.9368890	9.2971630
H	-0.4122160	8.1776610	9.9602290
H	-1.6790060	8.4979820	8.7648480
H	-1.2229450	9.7499500	9.9130440
C	6.6132700	5.8018320	7.2103560
H	5.9626290	5.6840420	8.0817870
H	7.4331730	6.4619400	7.5016280
H	7.0405540	4.8237940	6.9776830
C	0.4549890	5.5721280	3.3950500
H	1.1012600	6.1809940	2.7594260
H	-0.5607950	5.9681460	3.3348200
H	0.4435750	4.5556890	2.9939440
C	6.1618870	8.9936540	9.0247820

H	6.3498090	9.3954820	10.0237950
H	7.1261810	8.8517450	8.5312550
H	5.6989690	8.0093390	9.1408460
C	-0.3862340	10.5791280	7.4368730
H	-0.6330080	11.4357100	8.0695750
H	-1.2989560	10.2459450	6.9382560
H	0.3198740	10.9148280	6.6743610
C	6.8474320	6.6602420	4.8482310
H	7.3312500	5.7358190	4.5230360
H	7.6262020	7.3549480	5.1699930
H	6.3293200	7.0935190	3.9902000
C	6.0095530	11.2794990	7.9626960
H	5.3891520	11.9624050	7.3788880
H	6.9377840	11.1022840	7.4158380
H	6.2652300	11.7702600	8.9043330
C	3.1960280	5.5304110	10.6741910
C	2.0411470	5.1802520	11.3423630
C	4.3863650	5.2935550	11.3301240
C	2.0483420	4.6150920	12.6069240
C	4.4501730	4.7312710	12.5945100
C	3.2660010	4.3925400	13.2318430
F	0.8407250	5.3795820	10.7631380
F	0.9176730	4.2835860	13.2265910
F	3.2977520	3.8488490	14.4427800
F	5.6136110	4.5113380	13.2028820
F	5.5509150	5.6148170	10.7315950

DIPPZnC6F5-OPEt3-M062X

Zn	3.1657670	4.8930520	10.0346560
N	4.5178250	7.5193610	8.9087050
N	4.2933590	7.6291190	11.0303420
C	4.0531240	6.8296600	9.9668250
C	4.5261490	7.0193190	7.5585150
C	3.4628830	7.3609380	6.7150390
C	2.7187520	7.3348460	12.8830400
C	5.0451240	8.7379170	9.2935250
H	5.4679090	9.4269960	8.5820780
C	4.8996100	8.8081310	10.6385420
H	5.1806090	9.5707590	11.3454100
C	4.0272520	7.2226040	12.3914330
C	2.2692640	8.1601160	7.2038300

H	2.5033000	8.5622690	8.1923960
C	6.7418320	5.8441860	8.0933160
H	6.5993160	6.3597440	9.0468140
C	5.6048470	6.2226900	7.1584270
C	1.6272740	8.0154320	12.0722310
H	1.7603880	7.7363240	11.0219890
C	5.0893440	6.6841450	13.1307250
C	2.4787580	6.8360430	14.1631970
H	1.4798760	6.8789540	14.5775470
C	3.5087450	6.8814610	5.4070770
H	2.7017160	7.1177150	4.7226440
C	4.7913660	6.1904240	14.4004790
H	5.5765560	5.7495090	15.0025350
C	5.6018300	5.7656330	5.8392910
H	6.4209770	5.1490090	5.4861650
C	1.0570320	7.2309580	7.3537540
H	0.7335520	6.8647440	6.3747160
H	0.2198160	7.7667450	7.8074860
H	1.3030320	6.3609180	7.9679860
C	4.5690420	6.0963090	4.9720740
H	4.5885390	5.7369960	3.9495970
C	6.5044580	6.6085040	12.5773760
H	6.6172880	7.3845880	11.8168080
C	3.5001230	6.2588110	14.9064930
H	3.2862510	5.8570480	15.8903880
C	0.2119210	7.6167520	12.4934530
H	0.0863930	6.5343550	12.5315260
H	-0.5068970	8.0276280	11.7817630
H	-0.0342320	8.0264870	13.4762600
C	6.7363510	4.3359810	8.3752220
H	5.8252090	4.0248320	8.8934580
H	7.5812930	4.0659610	9.0130630
H	6.8151100	3.7644410	7.4459430
C	1.9487190	9.3455820	6.2894330
H	2.8116580	10.0056970	6.1811760
H	1.1233410	9.9242420	6.7094800
H	1.6460790	9.0117900	5.2941930
C	6.7511130	5.2567680	11.8997550
H	6.6674250	4.4446310	12.6276290
H	7.7546930	5.2257510	11.4666960
H	6.0298250	5.0689510	11.1016690

C	1.7796100	9.5417910	12.1734330
H	1.6731930	9.8557360	13.2151800
H	1.0034570	10.0375370	11.5858610
H	2.7505660	9.8835600	11.8126040
C	8.0960480	6.2887360	7.5291880
H	8.3378680	5.7518900	6.6087230
H	8.8868600	6.0827460	8.2537690
H	8.0983430	7.3583260	7.3100730
C	7.5674940	6.8706260	13.6485400
H	7.3623370	7.7907410	14.1990820
H	8.5471630	6.9628110	13.1754040
H	7.6275000	6.0475650	14.3640180
C	3.3558010	3.6467760	11.7145190
C	2.4459490	3.8052240	12.7369610
C	4.4550590	2.8727010	12.0204750
C	2.6028970	3.2705620	14.0046180
C	4.6716330	2.3105920	13.2685130
C	3.7358960	2.5201760	14.2698810
F	1.3304260	4.5390650	12.5207390
F	1.7144060	3.4968460	14.9737590
F	3.9312250	2.0078010	15.4825390
F	5.7710940	1.6003850	13.5298000
F	5.4104310	2.6508500	11.0878350
P	2.5076010	2.7249520	7.6579350
C	3.6063960	1.5666560	8.5289020
H	4.4001280	2.1764900	8.9691550
H	3.0306300	1.1582740	9.3656690
C	0.8247270	2.0594680	7.6466000
H	0.5170050	1.9872480	8.6933460
H	0.2011350	2.8292390	7.1835440
C	3.0608220	2.9014240	5.9458370
H	4.0317520	3.4061740	6.0008500
H	3.2226580	1.9119680	5.5108060
O	2.4983470	4.1046960	8.3364560
C	2.0616230	3.7168270	5.1204380
H	1.8229060	4.6580740	5.6192960
H	2.4860340	3.9514910	4.1438510
H	1.1363510	3.1599610	4.9591210
C	4.2175180	0.4562350	7.6680540
H	4.8937690	0.8708340	6.9192060
H	4.7950870	-0.2169470	8.3016210

H	3.4608560	-0.1364640	7.1529510
C	0.6738760	0.7156130	6.9302360
H	1.2084590	-0.0784650	7.4532770
H	-0.3785860	0.4344120	6.8929590
H	1.0417410	0.7622870	5.9027520

BCF-OPEt3-M062X

B	-5.6365840	-1.0623280	-0.0417410
C	-5.8536680	-2.1717440	-1.1257530
C	-5.3651100	0.4157360	-0.4816730
C	-5.4173240	-3.4840710	-0.9435520
C	-5.5964730	-4.4734450	-1.8924580
C	-6.2523370	-4.1672630	-3.0737530
C	-6.7120470	-2.8796960	-3.2966670
C	-6.5000780	-1.9103690	-2.3340220
C	-4.6484600	0.7255080	-1.6382310
C	-4.3987120	2.0238580	-2.0410870
C	-4.8902700	3.0739490	-1.2830320
C	-5.6146420	2.8166570	-0.1304670
C	-5.8317180	1.5063280	0.2524010
C	-6.5459330	-2.4302000	1.9620800
C	-5.6971070	-1.4340690	1.4778360
C	-4.9106110	-0.7912650	2.4340680
C	-4.9506580	-1.1123240	3.7780620
C	-5.8166070	-2.1030400	4.2118170
C	-6.6237740	-2.7645260	3.3008840
F	-7.3432990	-2.5896410	-4.4286410
F	-6.4391170	-5.1036100	-3.9884040
F	-5.1539050	-5.7077120	-1.6820100
F	-4.7819230	-3.8350240	0.1749470
F	-6.9636120	-0.6892270	-2.6023660
F	-3.7017040	2.2736130	-3.1434760
F	-4.6690030	4.3217020	-1.6598840
F	-6.0893750	3.8255600	0.5906680
F	-6.5385510	1.3173710	1.3668410
F	-4.1522260	-0.2456950	-2.4051770
F	-7.3469550	-3.0976420	1.1311190
F	-7.4572750	-3.7094420	3.7203460
F	-5.8744370	-2.4164370	5.4947680
F	-4.1744410	-0.4827090	4.6524180
F	-4.0579840	0.1678500	2.0730220

BCF-OPEt3-M062X

B	0.0903120	0.1810700	0.1783730
C	1.6419400	0.6420570	-0.0664620
C	2.1470410	1.9291130	0.0371520
C	2.5996670	-0.3368190	-0.2946120
C	3.4965890	2.2289140	-0.1007740
C	3.9514820	-0.0900490	-0.4324220
C	4.4050520	1.2152150	-0.3369070
C	-0.9757920	1.4168080	0.2258240
C	-1.0439350	2.2633840	-0.8743440
C	-1.9186290	1.6589060	1.2107300
C	-1.9435840	3.3042390	-0.9922750
C	-2.8451210	2.6914840	1.1326580
C	-2.8555710	3.5207530	0.0284690
C	-0.5009590	-0.8115030	-0.9954580
C	0.0332600	-1.0239140	-2.2595610
C	-1.7168320	-1.4430610	-0.7710920
C	-0.5651920	-1.8438720	-3.2069100
C	-2.3453220	-2.2762990	-1.6774590
C	-1.7569580	-2.4811030	-2.9138540
F	1.3486830	2.9750010	0.2913250
F	3.9237940	3.4867960	0.0055170
F	5.7004480	1.4874970	-0.4616440
F	4.8143800	-1.0859620	-0.6337110
F	2.2197780	-1.6282450	-0.3732480
F	-2.3398670	-1.2707410	0.4132970
F	-3.4920420	-2.8814250	-1.3702080
F	-2.3351690	-3.2740490	-3.8092480
F	-0.0005000	-2.0234170	-4.3993120
F	1.1762190	-0.4415420	-2.6386100
F	-0.1856410	2.0807130	-1.8909810
F	-2.0027820	0.8907430	2.3089830
F	-3.7300200	2.8825740	2.1124340
F	-3.7374160	4.5131780	-0.0598260
F	-1.9499640	4.0938730	-2.0659140
P	0.0533960	-1.8606990	2.2938930
O	0.1278300	-0.5103520	1.5328760
C	0.0895600	-3.3174910	1.1938600

H	0.9274090	-3.9308950	1.5372530
H	0.3616670	-2.9516470	0.2030040
C	1.4911980	-1.9496430	3.3861440
H	1.3726410	-1.1619440	4.1333040
H	1.4155430	-2.9093880	3.9082940
C	-1.4190590	-1.8987480	3.3463050
H	-1.5572920	-2.9328700	3.6732490
H	-2.2618940	-1.6365310	2.7056810
C	2.8304520	-1.7982760	2.6641550
H	2.9446850	-2.5112520	1.8456910
H	2.9270590	-0.7895300	2.2612250
H	3.6442440	-1.9591970	3.3709760
C	-1.3201940	-0.9537200	4.5489540
H	-2.2997620	-0.8633930	5.0183930
H	-0.6240230	-1.3354430	5.2962230
H	-1.0024550	0.0436400	4.2449810
C	-1.1925790	-4.1576700	1.1472750
H	-2.0714290	-3.5662050	0.8905220
H	-1.0777890	-4.9342660	0.3903690
H	-1.3819330	-4.6503080	2.1015020

HBCF-M062X

B	-0.0656590	0.0904780	-0.8694220
C	-0.2104440	1.5908570	-0.2361220
C	1.4045710	-0.5660150	-0.5813760
C	-0.9859890	2.5462510	-0.8791980
C	-1.1417000	3.8455150	-0.4195030
C	-0.5018730	4.2348850	0.7423310
C	0.2749200	3.3167690	1.4261950
C	0.3992540	2.0283380	0.9316130
C	2.4517340	-0.3500630	-1.4668670
C	3.7159190	-0.8997380	-1.3109700
C	3.9735250	-1.7073920	-0.2192750
C	2.9665410	-1.9427540	0.7001080
C	1.7196390	-1.3704040	0.5058620
C	-1.9243820	-0.8427540	0.8351400
C	-1.2726520	-0.9031910	-0.3889220
C	-1.7232170	-1.9056490	-1.2379720
C	-2.7523910	-2.7782740	-0.9188480
C	-3.3781420	-2.6675760	0.3090900
C	-2.9586860	-1.6918310	1.1955510

F	0.8829230	3.6810080	2.5597570
F	-0.6388020	5.4796490	1.2030640
F	-1.9014230	4.7241460	-1.0817360
F	-1.6447880	2.2411240	-2.0085610
F	1.1386830	1.1857780	1.6693240
F	4.6848470	-0.6602650	-2.2005710
F	5.1811300	-2.2484220	-0.0484940
F	3.2134810	-2.7096500	1.7669960
F	0.8065670	-1.6165860	1.4579410
F	2.2815260	0.4307150	-2.5454490
F	-1.5549510	0.0552600	1.7617290
F	-3.5491060	-1.5897910	2.3907420
F	-4.3680650	-3.4992560	0.6388970
F	-3.1444080	-3.7243630	-1.7784690
F	-1.1544470	-2.0827740	-2.4411470
H	-0.1662240	0.2064040	-2.0610270

DIPPZnC6F5H-M062X

Zn	0.4698520	-0.2571400	-1.7003720
N	2.3003200	-0.1744860	0.9313380
N	0.3575350	0.5186100	1.4628480
C	1.0816070	0.0782240	0.4065440
C	3.4544730	-0.4879400	0.1326270
C	4.1846180	0.5868120	-0.3899690
C	-1.2603170	2.2064380	0.7483980
C	2.3452570	0.0922580	2.2877640
H	3.2411290	-0.0440420	2.8693610
C	1.1106370	0.5298580	2.6254120
H	0.7055980	0.8588520	3.5673840
C	-1.0076670	0.9751890	1.3794070
C	3.7822940	2.0337410	-0.1563530
H	2.9336530	2.0545730	0.5317990
C	2.9464720	-2.9703490	0.4412930
H	2.1641230	-2.5528240	1.0791820
C	3.7821060	-1.8259200	-0.0999380
C	-0.1326180	3.0683620	0.1921270
H	0.5612830	2.4104500	-0.3396420
C	-2.0131170	0.1799260	1.9412260
C	-2.5885920	2.6213240	0.6780790
H	-2.8342720	3.5510530	0.1820280
C	5.2941030	0.2811640	-1.1767810

H	5.8802930	1.0842410	-1.6092070
C	-3.3264640	0.6440360	1.8399000
H	-4.1340220	0.0565250	2.2620870
C	4.9011860	-2.0802250	-0.8939910
H	5.1833690	-3.1059140	-1.1043690
C	3.3239620	2.6834290	-1.4675310
H	4.1508530	2.7447030	-2.1805170
H	2.9620940	3.6982020	-1.2794290
H	2.5238350	2.1039970	-1.9342670
C	5.6492300	-1.0387750	-1.4252610
H	6.5124780	-1.2574120	-2.0427310
C	-1.7189770	-1.0936240	2.7197390
H	-0.6721290	-1.3643980	2.5649220
C	-3.6112950	1.8484400	1.2158180
H	-4.6380940	2.1859860	1.1366880
C	-0.6030400	4.1314120	-0.8016880
H	-1.2173040	3.7025100	-1.5932230
H	0.2683620	4.6052060	-1.2587850
H	-1.1761650	4.9164070	-0.3006280
C	2.2631270	-3.7180870	-0.7101430
H	1.6858470	-3.0343530	-1.3370170
H	1.5871050	-4.4827510	-0.3211870
H	3.0041970	-4.2076270	-1.3476900
C	4.9145220	2.8362120	0.4924950
H	5.2297440	2.3824120	1.4347930
H	4.5799750	3.8563020	0.6962300
H	5.7852780	2.8949880	-0.1654200
C	-2.5650970	-2.2901630	2.2733810
H	-3.6351830	-2.0722280	2.3117260
H	-2.3761800	-3.1357000	2.9391680
H	-2.3145090	-2.6016800	1.2609300
C	0.6457610	3.7496800	1.3294800
H	-0.0128310	4.4323690	1.8736850
H	1.4751820	4.3318150	0.9176740
H	1.0577770	3.0344460	2.0426460
C	3.7860060	-3.9254470	1.2960490
H	4.5658940	-4.4054540	0.6995060
H	3.1516020	-4.7114330	1.7117730
H	4.2670230	-3.3967790	2.1218780
C	-1.9272640	-0.8282340	4.2187750
H	-1.3420220	0.0272090	4.5632520

H	-1.6387330	-1.7042060	4.8040580
H	-2.9799410	-0.6136980	4.4228910
C	-1.6453580	-0.6076340	-1.6004980
C	-2.5214170	0.4352750	-1.8085640
C	-2.2361370	-1.7789080	-1.1857280
C	-3.8880870	0.3593950	-1.5993790
C	-3.5971980	-1.9279770	-0.9662500
C	-4.4280990	-0.8391590	-1.1656740
F	-2.0466200	1.6374890	-2.2182460
F	-4.6835260	1.4223740	-1.7603170
F	-5.7367530	-0.9376590	-0.9210250
F	-4.1146300	-3.0816330	-0.5277650
F	-1.4715430	-2.8708770	-0.9319000
H	1.5362700	-0.1179610	-2.9899030

DIPPZnC6F5-B3LYPD3BJ

Zn	3.1921010	6.4395430	8.7895690
N	3.1383460	7.2587190	5.8215840
N	2.8406350	8.9455340	7.1169970
C	3.0222500	7.6083030	7.1183170
C	3.4277460	5.9087320	5.4044530
C	2.3714200	5.1023000	4.9589350
C	1.4875040	9.9006650	8.9172290
C	3.0296340	8.3711890	5.0019120
H	3.0989510	8.3003460	3.9310440
C	2.8373080	9.4386540	5.8220680
H	2.7103360	10.4851770	5.6093640
C	2.7506850	9.7138060	8.3366520
C	0.9414350	5.6095750	4.8711620
H	0.9219630	6.6420810	5.2238330
C	5.8975130	6.3640150	5.9503080
H	5.4846440	7.3342500	6.2292840
C	4.7550840	5.4640260	5.5030930
C	0.2163610	9.4050280	8.2476990
H	0.4972100	8.6600720	7.5000200
C	3.9440410	10.1840280	8.9039130
C	1.4418040	10.6049870	10.1222620
H	0.4881220	10.7796360	10.6034000
C	2.6818670	3.7892230	4.5978960
H	1.8953760	3.1321850	4.2482560
C	3.8419790	10.8763580	10.1132060

H	4.7384690	11.2538040	10.5888530
C	5.0089420	4.1402180	5.1343290
H	6.0196420	3.7561030	5.1926740
C	0.0011500	4.8001590	5.7785030
H	-0.0470360	3.7530280	5.4702030
H	-1.0108460	5.2095480	5.7323030
H	0.3305660	4.8275690	6.8203930
C	3.9850830	3.3131910	4.6867770
H	4.2045180	2.2905220	4.4040790
C	5.3014720	9.9568610	8.2572650
H	5.1441950	9.4850110	7.2861870
C	2.6057770	11.0853470	10.7132020
H	2.5479430	11.6271040	11.6496900
C	-0.7443030	8.7216980	9.2309250
H	-0.2538680	7.9135430	9.7785620
H	-1.5913140	8.2988230	8.6856120
H	-1.1458880	9.4272230	9.9618170
C	6.6302800	5.8047700	7.1804720
H	5.9728770	5.7059370	8.0478930
H	7.4465240	6.4723850	7.4648200
H	7.0599530	4.8215200	6.9755070
C	0.4466520	5.6141680	3.4155430
H	1.1037820	6.2105080	2.7782420
H	-0.5599360	6.0356890	3.3603240
H	0.4096280	4.6011920	3.0070360
C	6.1644650	9.0015270	9.0965750
H	6.3427580	9.4038790	10.0967270
H	7.1335090	8.8470960	8.6164550
H	5.6920350	8.0234600	9.2080890
C	-0.4776670	10.5631410	7.5087200
H	-0.7868230	11.3388600	8.2141290
H	-1.3670800	10.2025900	6.9861020
H	0.1882740	11.0225900	6.7746550
C	6.8758750	6.6085300	4.7888830
H	7.3571580	5.6786210	4.4760030
H	7.6582040	7.3067670	5.0962230
H	6.3594450	7.0299090	3.9234300
C	6.0284090	11.2856700	7.9991380
H	5.4161900	11.9564900	7.3920810
H	6.9662480	11.1027820	7.4692580
H	6.2678010	11.7985690	8.9335560

C	3.1969790	5.5622800	10.6372190
C	2.0303210	5.2443140	11.3029200
C	4.3832340	5.3315530	11.3051190
C	2.0200700	4.7160740	12.5863320
C	4.4310320	4.8053450	12.5885620
C	3.2344590	4.4987050	13.2280710
F	0.8315300	5.4431350	10.6953860
F	0.8743450	4.4142310	13.2120490
F	3.2518130	3.9908590	14.4641360
F	5.5940360	4.5911350	13.2183320
F	5.5616830	5.6286980	10.6981410

DIPPZnC6F5-OPEt3-B3LYPD3BJ

Zn	3.3042030	4.9339630	9.9203080
N	4.5934700	7.5202440	8.7084870
N	4.3739190	7.6915880	10.8342610
C	4.1555330	6.8512350	9.7958230
C	4.5538550	6.9720850	7.3762090
C	3.4360390	7.2443320	6.5769200
C	2.7155820	7.4144230	12.6080520
C	5.0840880	8.7716820	9.0546650
H	5.4820960	9.4510150	8.3221290
C	4.9405150	8.8813980	10.4002770
H	5.1929950	9.6771250	11.0783310
C	4.0539140	7.3247160	12.1941000
C	2.2606780	8.0644890	7.0807050
H	2.5132740	8.4509440	8.0692990
C	6.8167180	5.8693060	7.8495050
H	6.6973150	6.4253890	8.7805300
C	5.6220860	6.1683450	6.9577800
C	1.6583390	8.0558980	11.7189980
H	1.9172320	7.8380920	10.6793350
C	5.0852160	6.8310070	13.0059490
C	2.4131780	6.9387610	13.8850600
H	1.3929660	6.9702790	14.2416340
C	3.4156630	6.6896420	5.2959830
H	2.5698380	6.8781240	4.6466200
C	4.7246780	6.3523100	14.2670640
H	5.4827570	5.9387380	14.9190530
C	5.5478030	5.6254880	5.6722620
H	6.3529190	4.9971650	5.3117410

C	1.0129140	7.1788970	7.2407050
H	0.6624390	6.8194380	6.2692050
H	0.2008690	7.7490050	7.6995330
H	1.2271640	6.3045540	7.8581380
C	4.4588950	5.8870430	4.8488990
H	4.4214110	5.4604540	3.8534310
C	6.5318480	6.7953250	12.5322580
H	6.6447140	7.5450330	11.7468470
C	3.4033060	6.3957620	14.6958110
H	3.1417800	6.0048580	15.6718750
C	0.2412510	7.5209510	11.9527970
H	0.2056780	6.4336120	11.8854710
H	-0.4339730	7.9383620	11.2020310
H	-0.1436840	7.8137480	12.9328480
C	6.8766260	4.3775940	8.2150080
H	5.9827310	4.0635890	8.7560760
H	7.7361400	4.1794620	8.8594520
H	6.9719030	3.7537890	7.3222560
C	1.9797200	9.2746950	6.1781390
H	2.8631700	9.9109780	6.0873770
H	1.1665550	9.8745630	6.5944000
H	1.6834260	8.9631230	5.1733000
C	6.8839370	5.4350500	11.9134420
H	6.7999680	4.6367740	12.6544570
H	7.9110060	5.4435460	11.5393260
H	6.2258640	5.1892650	11.0810200
C	1.6894930	9.5868600	11.8875660
H	1.4466550	9.8605240	12.9177920
H	0.9564460	10.0566390	11.2266620
H	2.6718450	9.9992130	11.6510000
C	8.1299920	6.3397050	7.2051580
H	8.3438390	5.7915440	6.2840300
H	8.9645150	6.1770830	7.8917960
H	8.0888880	7.4040670	6.9620060
C	7.5218950	7.1625190	13.6461770
H	7.2605120	8.1142560	14.1144120
H	8.5285710	7.2499100	13.2306250
H	7.5557180	6.3975690	14.4254680
C	3.4753750	3.6827620	11.5724050
C	2.5316290	3.7669930	12.5724360
C	4.6071070	2.9575600	11.8798140

C	2.6981860	3.2283960	13.8385680
C	4.8352380	2.3905930	13.1256080
C	3.8714650	2.5393790	14.1158460
F	1.3647320	4.4256680	12.3286030
F	1.7718790	3.3820380	14.7991630
F	4.0735320	2.0191410	15.3337550
F	5.9726640	1.7287130	13.3986070
F	5.5828700	2.7928900	10.9428950
P	2.4655090	2.7073290	7.6883250
C	3.6783140	1.5854130	8.4649780
H	4.5694790	2.1929560	8.6369020
H	3.2781660	1.3443640	9.4536130
C	0.7827200	2.1245560	8.0645390
H	0.6872340	2.1874310	9.1525650
H	0.1099780	2.8780890	7.6465220
C	2.7066210	2.6958350	5.8892860
H	3.7129480	3.0914490	5.7239080
H	2.7003530	1.6608760	5.5394390
O	2.6286120	4.1453980	8.2188280
C	1.6608550	3.5385080	5.1507680
H	1.6217190	4.5503130	5.5531050
H	1.9184880	3.6062480	4.0927020
H	0.6660230	3.0949620	5.2245300
C	4.0442330	0.3170220	7.6850820
H	4.5276060	0.5590340	6.7373510
H	4.7456740	-0.2781820	8.2721130
H	3.1756900	-0.3075730	7.4746020
C	0.4231440	0.7235580	7.5601570
H	1.0277910	-0.0452560	8.0426300
H	-0.6226960	0.5093430	7.7858790
H	0.5527080	0.6349320	6.4798000

BCF-B3LYPD3BJ

B	0.0000470	0.0009610	-0.0004540
C	1.4871220	0.4740940	0.0006390
C	-1.1537310	1.0518300	-0.0008550
C	2.4752910	-0.1885500	0.7343760
C	3.7977720	0.2227860	0.7534870
C	4.1760690	1.3272750	0.0004800
C	3.2300470	2.0116590	-0.7524440
C	1.9122870	1.5854760	-0.7328350

C	-1.0717860	2.2429790	0.7262120
C	-2.0906880	3.1810420	0.7464560
C	-3.2401300	2.9509160	0.0008670
C	-3.3621060	1.7855250	-0.7455240
C	-2.3324700	0.8592350	-0.7273180
C	0.4218760	-2.4476330	-0.7285820
C	-0.3331670	-1.5238110	-0.0002770
C	-1.4050550	-2.0486760	0.7274700
C	-1.7086690	-3.3999500	0.7462630
C	-0.9357650	-4.2797690	-0.0012060
C	0.1338250	-3.8023420	-0.7482360
F	3.5997330	3.0684950	-1.4822540
F	5.4445700	1.7296760	0.0003920
F	4.7092510	-0.4276490	1.4830840
F	2.1588210	-1.2556760	1.4851400
F	1.0382030	2.2752250	-1.4830230
F	-1.9809450	4.2994230	1.4698580
F	-4.2243520	3.8466580	0.0017490
F	-4.4661270	1.5717500	-1.4677440
F	-2.4956340	-0.2464140	-1.4711880
F	0.0142870	2.5083710	1.4694980
F	1.4599120	-2.0351780	-1.4735630
F	0.8695250	-4.6509010	-1.4726940
F	-1.2199460	-5.5798820	-0.0016210
F	-2.7312830	-3.8645780	1.4705390
F	-2.1765210	-1.2412440	1.4727600

BCF-OPEt3-B3LYPD3BJ

B	0.0745300	0.1698690	0.1462630
C	1.6049280	0.6480300	-0.1877460
C	2.0858350	1.9472910	-0.0609500
C	2.5765270	-0.2946710	-0.5077670
C	3.4165260	2.2941590	-0.2747410
C	3.9113120	-0.0002350	-0.7249540
C	4.3369010	1.3154500	-0.6119370
C	-1.0058810	1.3970020	0.2214690
C	-1.1917960	2.1851860	-0.9123100
C	-1.8513540	1.6957920	1.2819740
C	-2.1194030	3.2088600	-1.0020180
C	-2.7989720	2.7144340	1.2358360
C	-2.9347880	3.4776430	0.0889650

C	-0.5822750	-0.8737450	-0.9469630
C	-0.1547630	-1.0770370	-2.2569650
C	-1.7569420	-1.5438000	-0.6181320
C	-0.8061950	-1.9216750	-3.1492390
C	-2.4338080	-2.4033130	-1.4680720
C	-1.9493660	-2.5978780	-2.7525700
F	1.2741800	2.9642570	0.2960710
F	3.8182600	3.5677930	-0.1466690
F	5.6217430	1.6309360	-0.8124910
F	4.7934050	-0.9692250	-1.0143480
F	2.2409120	-1.6068910	-0.5911640
F	-2.2990210	-1.3845220	0.6165250
F	-3.5352630	-3.0500890	-1.0570690
F	-2.5792450	-3.4215920	-3.5972500
F	-0.3384300	-2.0877180	-4.3947850
F	0.9325390	-0.4503670	-2.7440440
F	-0.4202100	1.9699600	-2.0016220
F	-1.8114900	1.0026810	2.4410310
F	-3.5876040	2.9587830	2.2952460
F	-3.8423500	4.4611130	0.0307190
F	-2.2386050	3.9390340	-2.1216690
P	0.3130960	-1.7916640	2.3461790
O	0.1991320	-0.4785520	1.5278220
C	0.3844230	-3.2960010	1.3000310
H	1.2769010	-3.8407270	1.6181230
H	0.5813050	-2.9475700	0.2873040
C	1.8367230	-1.7303490	3.3326710
H	1.7189210	-0.9099190	4.0435130
H	1.8469560	-2.6591010	3.9128930
C	-1.0717330	-1.9135930	3.5187020
H	-1.0905570	-2.9450730	3.8801010
H	-1.9769610	-1.7561210	2.9319080
C	3.1275510	-1.5553310	2.5286370
H	3.2332590	-2.3036010	1.7418500
H	3.1601050	-0.5687410	2.0680280
H	3.9864670	-1.6442640	3.1952750
C	-0.9941270	-0.9285120	4.6919850
H	-1.9259690	-0.9708050	5.2581730
H	-0.1802480	-1.1796050	5.3734270
H	-0.8605010	0.0952290	4.3455730
C	-0.8396830	-4.2198280	1.3391840

H	-1.7593170	-3.7005210	1.0749690
H	-0.6950900	-5.0293650	0.6219260
H	-0.9746540	-4.6704360	2.3234990

DIPPZnC6F5H-B3LYPD3BJ

Zn	0.4244560	-0.1218690	-1.6485680
N	2.3299400	0.0301580	0.9098820
N	0.3982640	0.7081870	1.5228620
C	1.0696820	0.2193190	0.4471780
C	3.4163810	-0.4461730	0.0961730
C	4.2303640	0.4995710	-0.5421910
C	-1.3419890	2.3089110	0.9091530
C	2.4506070	0.3893890	2.2455150
H	3.3806660	0.3061850	2.7791190
C	1.2275510	0.8225940	2.6324230
H	0.8721140	1.1956940	3.5763470
C	-0.9932950	1.0835860	1.4986180
C	3.9996240	1.9950820	-0.3959170
H	3.1544900	2.1434690	0.2776180
C	2.7047600	-2.8331460	0.6770740
H	2.0221830	-2.2820470	1.3256320
C	3.5999150	-1.8290210	-0.0310530
C	-0.2759800	3.2790160	0.4155720
H	0.5346730	2.6872060	-0.0166640
C	-1.9295240	0.2072490	2.0696830
C	-2.7015170	2.6203030	0.8453960
H	-3.0186870	3.5418080	0.3774360
C	5.2647630	0.0161480	-1.3456780
H	5.9113640	0.7155410	-1.8617230
C	-3.2760510	0.5601640	1.9648350
H	-4.0358110	-0.0958340	2.3679160
C	4.6433610	-2.2629830	-0.8526580
H	4.8118130	-3.3246540	-0.9855540
C	3.6240810	2.6340870	-1.7423210
H	4.4518710	2.5716080	-2.4540040
H	3.3797270	3.6908320	-1.6028720
H	2.7632180	2.1298460	-2.1852940
C	5.4663790	-1.3505770	-1.5027660
H	6.2696810	-1.7068930	-2.1371200
C	-1.4918600	-1.0657650	2.7854910
H	-0.5238590	-0.8619540	3.2483670

C	-3.6569350	1.7472800	1.3500060
H	-4.7083500	1.9939250	1.2636900
C	-0.7624600	4.2479540	-0.6676780
H	-1.2302300	3.7212250	-1.4980380
H	0.0874230	4.8167730	-1.0525020
H	-1.4808140	4.9691850	-0.2681140
C	1.8479490	-3.6184370	-0.3274440
H	1.2075600	-2.9541590	-0.9096470
H	1.2009200	-4.3257630	0.1977510
H	2.4755400	-4.1823090	-1.0226330
C	5.2146600	2.6897490	0.2381230
H	5.4551740	2.2525740	1.2104740
H	5.0090990	3.7537510	0.3822950
H	6.0989470	2.6026450	-0.3986890
C	-1.2844080	-2.2328430	1.8097930
H	-2.2327420	-2.5353920	1.3616940
H	-0.8713210	-3.0960080	2.3390160
H	-0.6019810	-1.9691590	1.0036710
C	0.3085710	4.0729660	1.5999280
H	-0.4703860	4.6823240	2.0670920
H	1.1016370	4.7410170	1.2529790
H	0.7303260	3.4187780	2.3634850
C	3.5198520	-3.7774130	1.5748160
H	4.1958780	-4.4052390	0.9883800
H	2.8497700	-4.4384660	2.1305640
H	4.1210370	-3.2155560	2.2939420
C	-2.4444670	-1.4749350	3.9160950
H	-2.6259420	-0.6501440	4.6094080
H	-2.0095440	-2.3049930	4.4779330
H	-3.4087230	-1.8145550	3.5296600
C	-1.6638320	-0.5522240	-1.6332250
C	-2.5820150	0.4652640	-1.7650000
C	-2.1940900	-1.7870360	-1.3328930
C	-3.9447090	0.3122080	-1.5594510
C	-3.5442410	-2.0092370	-1.1013580
C	-4.4252610	-0.9413670	-1.2083230
F	-2.1535940	1.7179630	-2.1017380
F	-4.8000840	1.3489390	-1.6541790
F	-5.7352560	-1.1183300	-0.9650720
F	-4.0121410	-3.2225160	-0.7431620
F	-1.3699990	-2.8702400	-1.2136670

H	1.4866590	0.0759620	-2.9109190

HBCF-B3LYPD3BJ

B	0.0433020	-0.0692580	-0.8943780
C	-0.1626980	1.4692680	-0.3699310
C	1.5033060	-0.6897100	-0.5070740
C	-1.0933240	2.2825580	-1.0109020
C	-1.3575050	3.5939350	-0.6395090
C	-0.6720910	4.1468850	0.4317800
C	0.2588640	3.3749490	1.1086350
C	0.4888360	2.0657770	0.7032700
C	2.6487170	-0.0246880	-0.9418750
C	3.9406470	-0.4705880	-0.7131820
C	4.1364270	-1.6544550	-0.0163250
C	3.0324440	-2.3595470	0.4311200
C	1.7535080	-1.8718310	0.1794860
C	-1.6371600	-0.9808290	0.9475670
C	-1.2367030	-0.9449270	-0.3836550
C	-2.0424010	-1.6639770	-1.2574130
C	-3.1735250	-2.3649520	-0.8522220
C	-3.5341890	-2.3634380	0.4854210
C	-2.7549840	-1.6659290	1.3981140
F	0.9229140	3.9012800	2.1551750
F	-0.9107020	5.4138100	0.8111690
F	-2.2675710	4.3341420	-1.3005480
F	-1.8054060	1.8030240	-2.0567260
F	1.4004990	1.3787950	1.4281820
F	5.0047070	0.2251050	-1.1561540
F	5.3791430	-2.1091660	0.2178310
F	3.2081480	-3.5112150	1.1062410
F	0.7410400	-2.6358640	0.6547150
F	2.5307400	1.1374500	-1.6260640
F	-0.9021010	-0.3430930	1.8873050
F	-3.0923380	-1.6703920	2.7015060
F	-4.6235220	-3.0341060	0.8965660
F	-3.9227210	-3.0442140	-1.7413790
F	-1.7530730	-1.7225460	-2.5764960
H	0.0066800	-0.0318220	-2.0998400

DIPPZnH-HBCat-B3PW91

C	-4.5402180	1.7743630	0.3075510
C	-4.3097900	2.7455120	-0.6076340
C	-2.5289360	1.3616460	-0.6222240
C	-3.3373860	-0.2454170	1.1055800
C	-2.4846300	3.2764200	-2.2137090
C	-2.7647870	2.9414520	-3.5493960
C	-2.2033580	3.7480170	-4.5405170
C	-1.4085140	4.8377770	-4.2150920
C	-1.1581070	5.1483830	-2.8861730
C	-1.6921940	4.3780680	-1.8516250
C	-3.9147490	-1.4370120	0.6360420
C	-3.8478640	-2.5527280	1.4729240
C	-3.2379170	-2.4808830	2.7167270
C	-2.6887310	-1.2849590	3.1582420
C	-2.7343630	-0.1338390	2.3695270
C	-2.2118170	1.1825930	2.9188050
C	-4.6169160	-1.5480280	-0.7071610
C	-3.9555540	-2.5962260	-1.6100580
C	-6.1108790	-1.8468260	-0.5224390
C	-1.4232520	4.7648960	-0.4078310
C	0.0781460	4.8412260	-0.1063050
C	-2.1196860	6.0873230	-0.0568310
C	-3.6583040	1.7775480	-3.9422960
C	-4.9757140	2.2814190	-4.5483180
C	-2.9526240	0.8073650	-4.8973360
C	-3.1709430	1.7341350	3.9843230
C	-0.7897920	1.0675640	3.4783710
Zn	-0.6471400	0.6026670	-1.2324050
C	1.9230910	-2.2449400	1.6220010
C	1.6631120	-1.0209320	1.0199510
C	2.5682580	0.0193720	1.0488040
C	3.7685190	-0.2232180	1.7230490
C	4.0291200	-1.4513480	2.3313220
C	3.1027250	-2.4961310	2.2918740
H	-5.3777340	1.6022510	0.9621450
H	-4.9043430	3.5912350	-0.9088560
H	-2.3959690	3.5208480	-5.5832720
H	-0.9843590	5.4513390	-5.0027200
H	-0.5399840	6.0064830	-2.6461780
H	-4.2824960	-3.4909730	1.1455320
H	-3.1966740	-3.3602840	3.3505180

H	-2.2325300	-1.2397030	4.1409040
H	-2.1815840	1.9048040	2.0984060
H	-4.5384810	-0.5842680	-1.2172430
H	-4.4530760	-2.6227970	-2.5835200
H	-2.8981300	-2.3740900	-1.7768470
H	-4.0218780	-3.5981190	-1.1771330
H	-6.6149720	-1.8672090	-1.4928090
H	-6.5975290	-1.0896080	0.0977730
H	-6.2641430	-2.8193980	-0.0458990
H	-1.8468370	3.9908570	0.2376690
H	0.2381730	5.0460400	0.9559960
H	0.5874000	3.9066900	-0.3549990
H	0.5619190	5.6421310	-0.6721930
H	-1.9596330	6.3319560	0.9970710
H	-3.1970920	6.0340960	-0.2337780
H	-1.7230120	6.9120900	-0.6560660
H	-3.9092540	1.2177370	-3.0372920
H	-5.6322180	1.4382870	-4.7818120
H	-5.5059800	2.9448270	-3.8600900
H	-4.7969210	2.8335930	-5.4756920
H	-3.5975750	-0.0513620	-5.1035970
H	-2.0147730	0.4356450	-4.4761880
H	-2.7214740	1.2814730	-5.8552120
H	-2.8210240	2.7064340	4.3428740
H	-4.1829410	1.8609510	3.5912860
H	-3.2273380	1.0588300	4.8432860
H	-0.7505800	0.4199950	4.3586750
H	-0.4290000	2.0534860	3.7845710
H	-0.0952220	0.6660650	2.7370190
H	0.6565670	0.5725690	-2.1833660
H	2.3722350	0.9713880	0.5726880
H	4.5125020	0.5636450	1.7702290
H	4.9719070	-1.6012350	2.8448760
H	3.2942700	-3.4548080	2.7579820
H	-1.1022040	-2.7690660	0.3255340
N	-3.4419300	0.9367800	0.2832630
N	-3.0765990	2.4764600	-1.1659760
B	-0.0634950	-2.3629490	0.7022040
O	0.4038730	-1.0767950	0.4406860
O	0.8512070	-3.0872100	1.4262540

HBCat-B3PW91

C	-0.2713900	0.6954550	0.0000340
C	-0.2713910	-0.6954550	-0.0000970
C	0.8978170	-1.4288730	-0.0000290
C	2.0894620	-0.6979490	0.0001760
C	2.0894630	0.6979490	0.0003060
C	0.8978170	1.4288730	0.0002390
H	0.8858900	-2.5121820	-0.0001260
H	3.0343310	-1.2299030	0.0002380
H	3.0343310	1.2299030	0.0004660
H	0.8858900	2.5121820	0.0003400
B	-2.3486670	0.0000000	-0.0003000
H	-3.5294400	0.0000000	-0.0004080
O	-1.5723960	1.1428770	-0.0000710
O	-1.5723960	-1.1428770	-0.0002780

DIPPZnH-HBPin-B3PW91

Zn	2.5039470	6.0843190	9.3440170
N	3.3590320	7.3809710	6.6562620
N	2.6639610	8.9365180	7.9605510
C	2.7515780	7.5902960	7.8500250
C	3.6803730	6.0832870	6.1080670
C	2.8433250	5.5551140	5.1118020
C	0.7903690	10.1090750	9.0144170
C	3.6437360	8.5786040	6.0290120
H	4.1244980	8.6250870	5.0667050
C	3.2056440	9.5601680	6.8520500
H	3.2333810	10.6318230	6.7522300
C	2.1166630	9.6496250	9.0898690
C	1.6296260	6.2913570	4.5703860
H	1.4725340	7.1866410	5.1772040
C	5.8071340	6.0632130	7.5441000
H	5.2452800	6.7757870	8.1562390
C	4.8397160	5.4245010	6.5610060
C	-0.0796250	9.9074700	7.7849720
H	0.3828580	9.1324010	7.1674160
C	2.9523450	9.9050540	10.1908510
C	0.2974770	10.8209900	10.1093870
H	-0.7211430	11.1914510	10.0897930
C	3.1818920	4.3063920	4.5861700

H	2.5582110	3.8675570	3.8153140
C	2.4028350	10.6191660	11.2574800
H	3.0152420	10.8350560	12.1254850
C	5.1202350	4.1753830	6.0057220
H	5.9996110	3.6334270	6.3323070
C	0.3532950	5.4480040	4.6656900
H	0.4101340	4.5552110	4.0367230
H	-0.5076220	6.0325170	4.3290930
H	0.1651050	5.1257860	5.6920330
C	4.3006720	3.6200450	5.0320130
H	4.5423640	2.6490260	4.6130850
C	4.4156860	9.4976020	10.2244870
H	4.5814480	8.7620990	9.4322890
C	1.0909010	11.0686560	11.2205660
H	0.6866210	11.6241790	12.0601020
C	-1.4942360	9.4310860	8.1300250
H	-1.4781970	8.5338160	8.7530630
H	-2.0417420	9.1981120	7.2125910
H	-2.0639950	10.1982720	8.6612550
C	6.4590680	5.0612360	8.5012070
H	5.7198340	4.4445450	9.0184000
H	7.0367560	5.5989700	9.2574910
H	7.1529530	4.3945720	7.9814440
C	1.8727080	6.7494120	3.1250000
H	2.7615900	7.3805180	3.0431660
H	1.0150910	7.3219340	2.7603040
H	2.0104830	5.8918250	2.4597530
C	4.8219680	8.8400290	11.5476920
H	4.7686710	9.5427370	12.3838470
H	5.8559830	8.4900350	11.4846090
H	4.1898630	7.9805170	11.7859710
C	-0.1388840	11.1961460	6.9503510
H	-0.6051400	12.0055340	7.5200110
H	-0.7318310	11.0349510	6.0455580
H	0.8556900	11.5321790	6.6471930
C	6.8863000	6.8498180	6.7830570
H	7.4859160	6.1741300	6.1656330
H	7.5577400	7.3516570	7.4857550
H	6.4531970	7.6087430	6.1271520
C	5.3103190	10.7094520	9.9231240
H	5.0668520	11.1608390	8.9578570

H	6.3618780	10.4092260	9.9022670
H	5.1940340	11.4782780	10.6928950
H	3.1632580	5.4218070	10.6692830
C	-0.7037700	4.6897680	9.5001160
C	-0.9063320	3.1868170	9.8920340
O	0.6665600	4.6642350	8.9301040
O	-0.0528880	2.4951650	8.9245820
B	0.8925010	3.3641350	8.5121570
H	1.8362700	3.0667530	7.8584500
C	-0.3606230	2.8409730	11.2730700
H	-0.9891950	3.2569650	12.0635850
H	-0.3466950	1.7547000	11.3836370
H	0.6595480	3.2095360	11.4060210
C	-2.3261100	2.6742870	9.7314480
H	-3.0077700	3.2187530	10.3901220
H	-2.6785020	2.7695950	8.7042590
H	-2.3617280	1.6183900	10.0077960
C	-1.6219560	5.1439460	8.3739690
H	-1.2907980	6.1166960	8.0053620
H	-1.6121960	4.4399690	7.5392370
H	-2.6501010	5.2459340	8.7273440
C	-0.7430920	5.6657220	10.6585160
H	-1.7311650	5.6501150	11.1259560
H	0.0006990	5.4328920	11.4205800
H	-0.5715730	6.6831210	10.2980640

HBPin-B3PW91

C	-0.7843830	-0.1908260	-0.0480530
C	0.7844240	-0.1907320	0.0480570
B	-0.0001120	1.9406030	-0.0000290
H	-0.0001690	3.1299830	-0.0001030
C	-1.4723370	-0.4639680	1.2863100
H	-2.5394360	-0.2536630	1.1830620
H	-1.3553340	-1.5070040	1.5898950
H	-1.0752520	0.1762600	2.0777140
C	1.3627630	-1.0819600	1.1337540
H	1.1145850	-2.1303130	0.9458540
H	2.4514590	-0.9888660	1.1397030
H	0.9924170	-0.8068210	2.1218760
C	1.4723990	-0.4638810	-1.2862930
H	2.5394720	-0.2534390	-1.1830690

H	1.3555210	-1.5069520	-1.5898070
H	1.0752270	0.1762450	-2.0777370
C	-1.3626120	-1.0821840	-1.1337010
H	-1.1143250	-2.1305000	-0.9457330
H	-2.4513180	-0.9892060	-1.1396700
H	-0.9922830	-0.8070670	-2.1218350
O	1.0759540	1.1994170	0.3861650
O	-1.0760670	1.1992690	-0.3862470

Optimised structures of O-bound CatBH-[IDippZnH]$^+$ (left) and PinBH-[IDippZnH]$^+$ (right) at the B3PW91/6-311G(d,p)/lanl2dz(Zn)/PCM (PhCl) level.

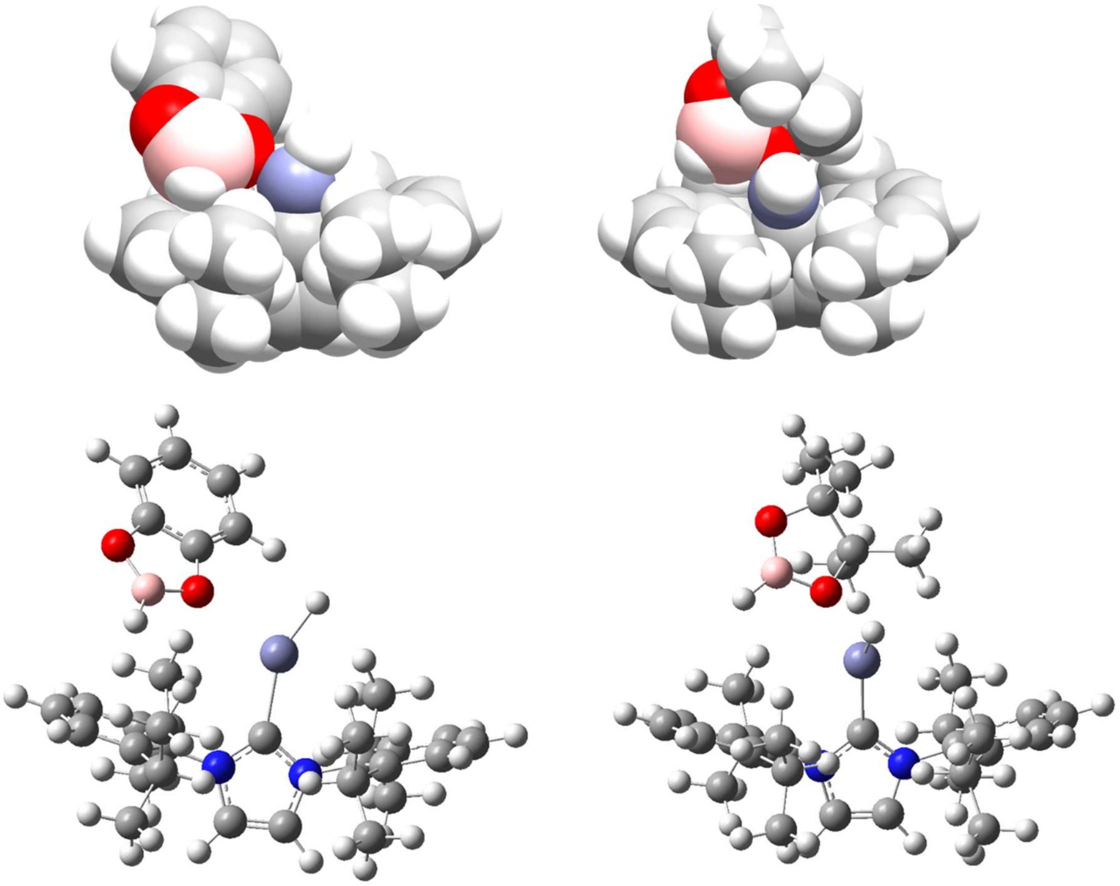

Figure S75: Top space filling diagram at 100% van der Waals radii of O-bound adducts.

S15. Copies of NMR for new Zinc compounds

S15.1 7DippZnEt$_2$

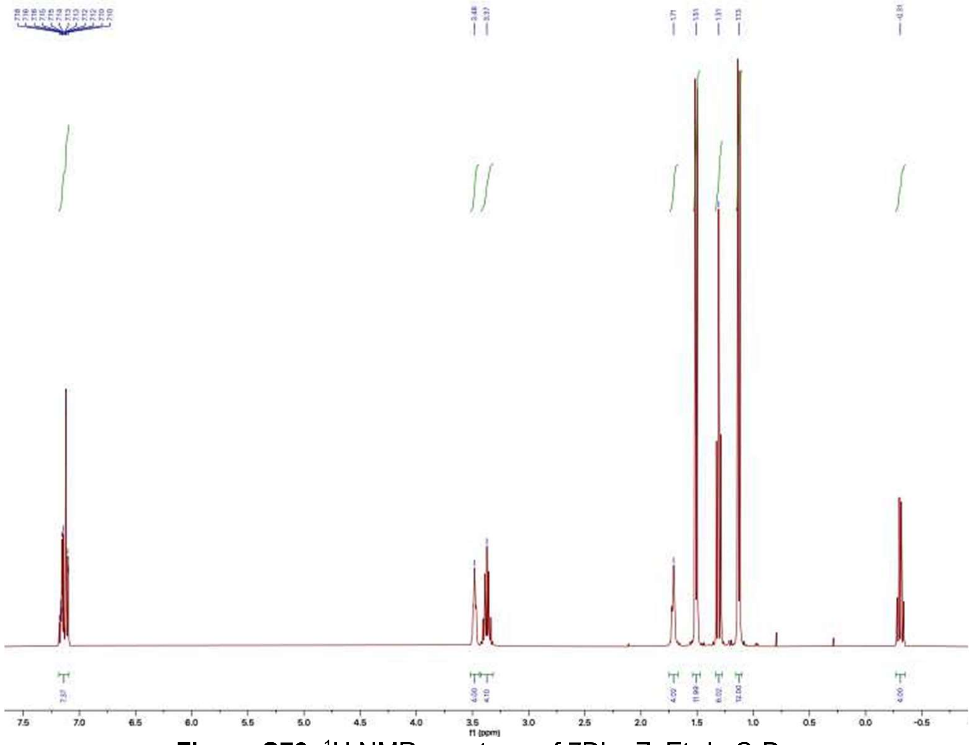

Figure S76: ^{1}H NMR spectrum of 7DippZnEt$_2$ in C_6D_6.

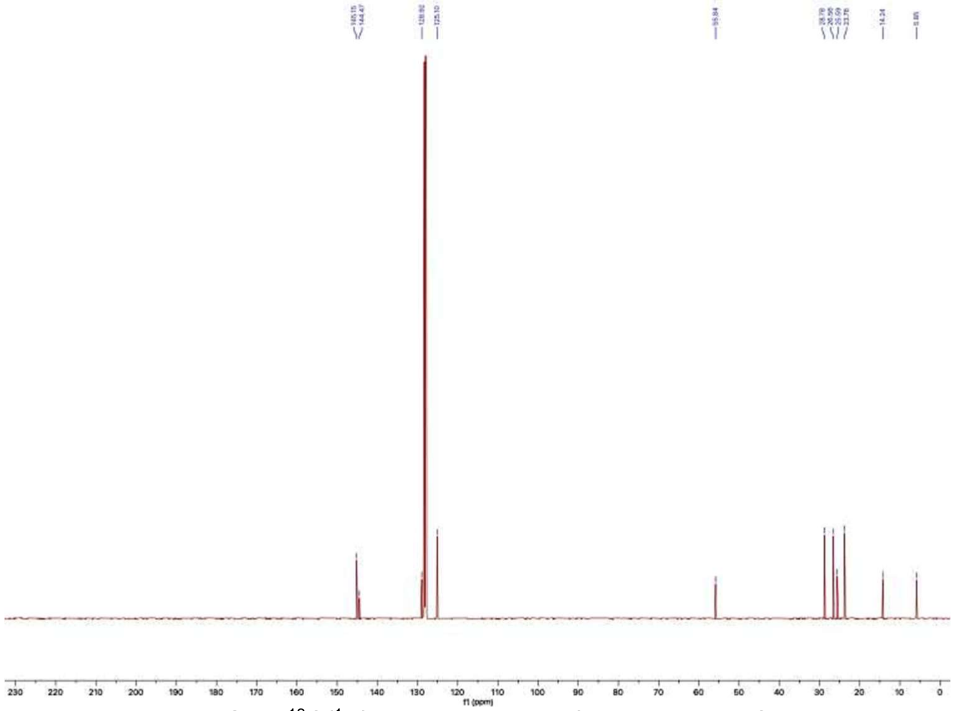

Figure S77: $^{13}C\{^1H\}$ NMR spectrum of 7DippZnEt$_2$ in C_6D_6.

S15.2 [7DippZnEt][B(C_6F_5)$_4$] (4)

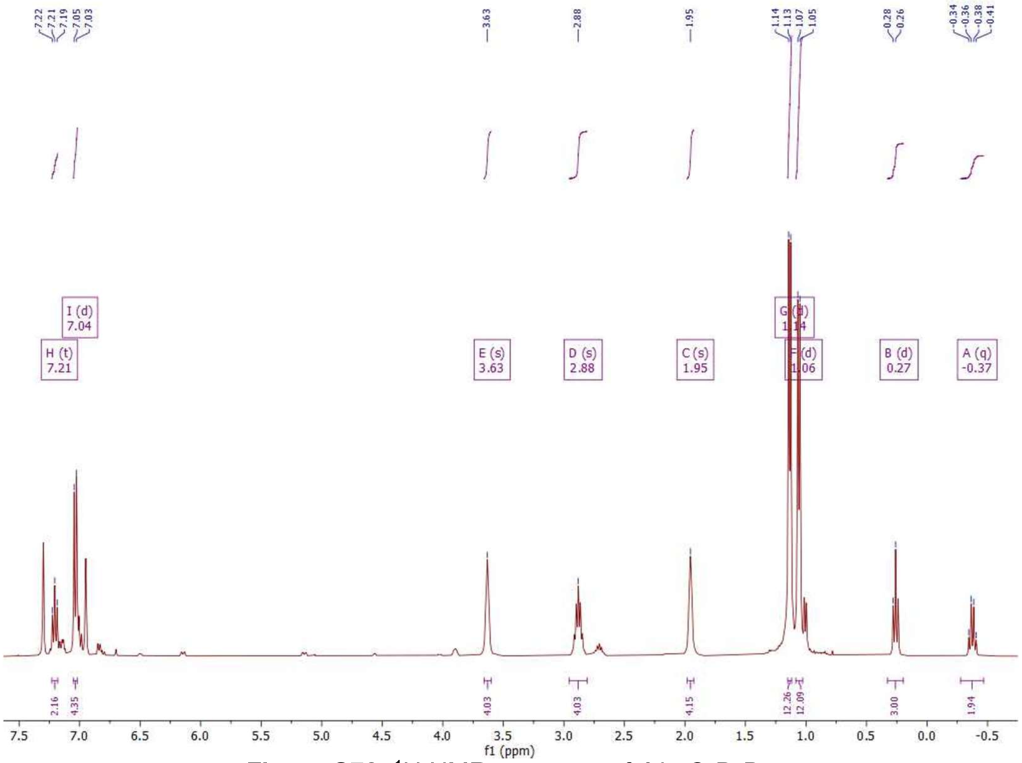

Figure S78: ^{1}H NMR spectrum of **4** in C_6D_5Br.

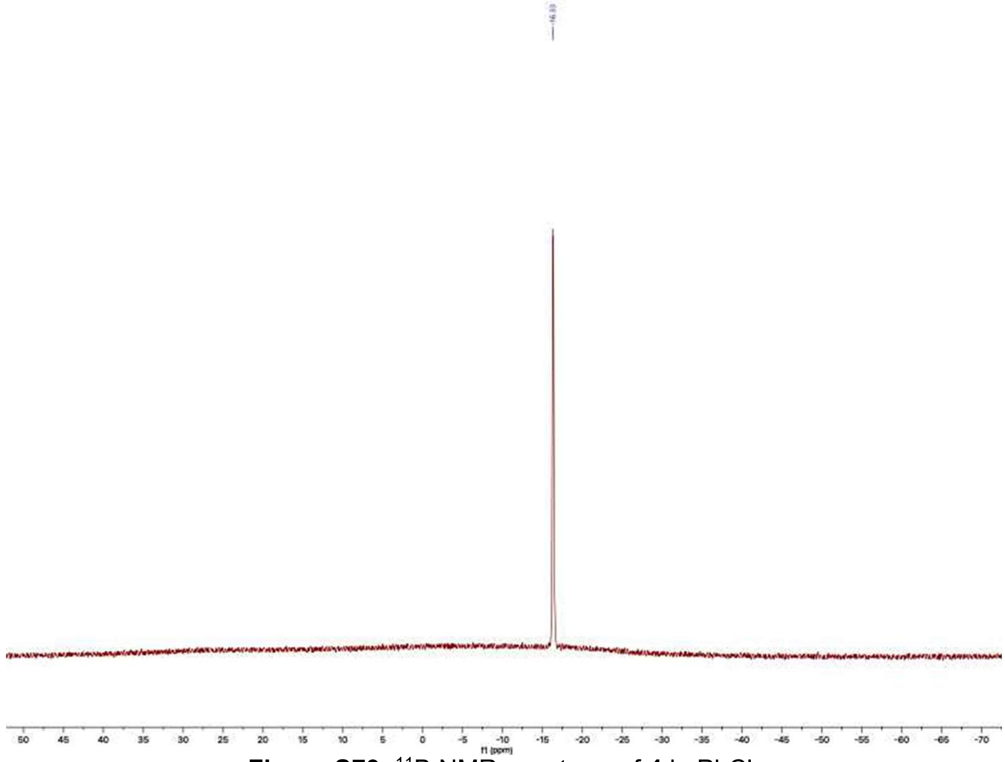

Figure S79: ^{11}B NMR spectrum of **4** in PhCl.

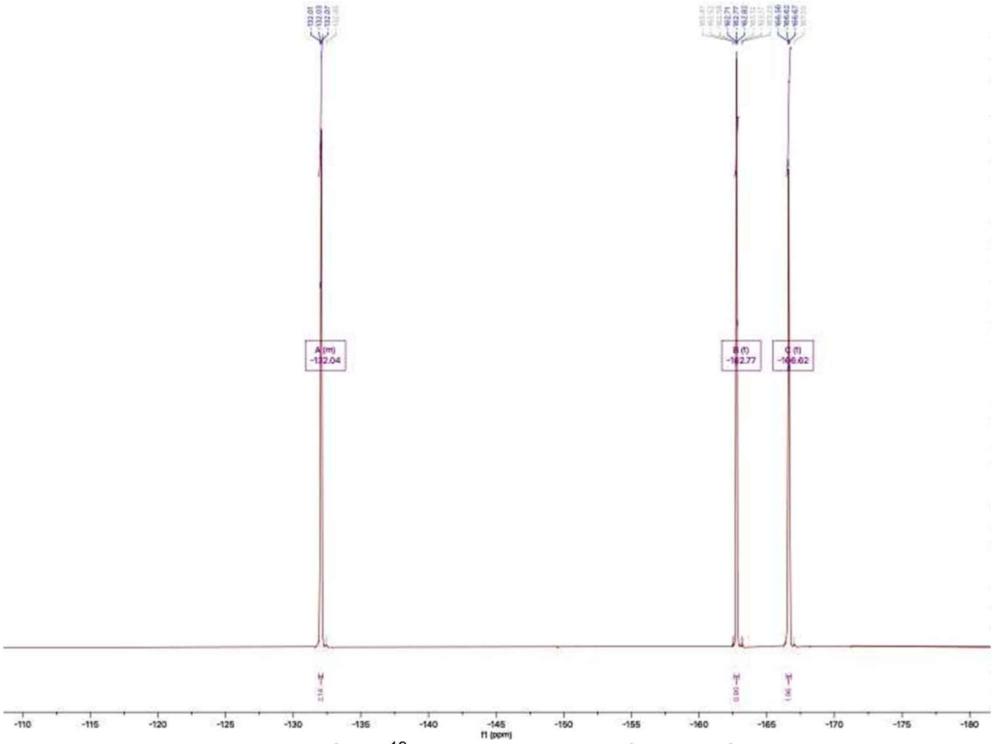

Figure S80: ^{19}F NMR spectrum of **4** in PhCl.

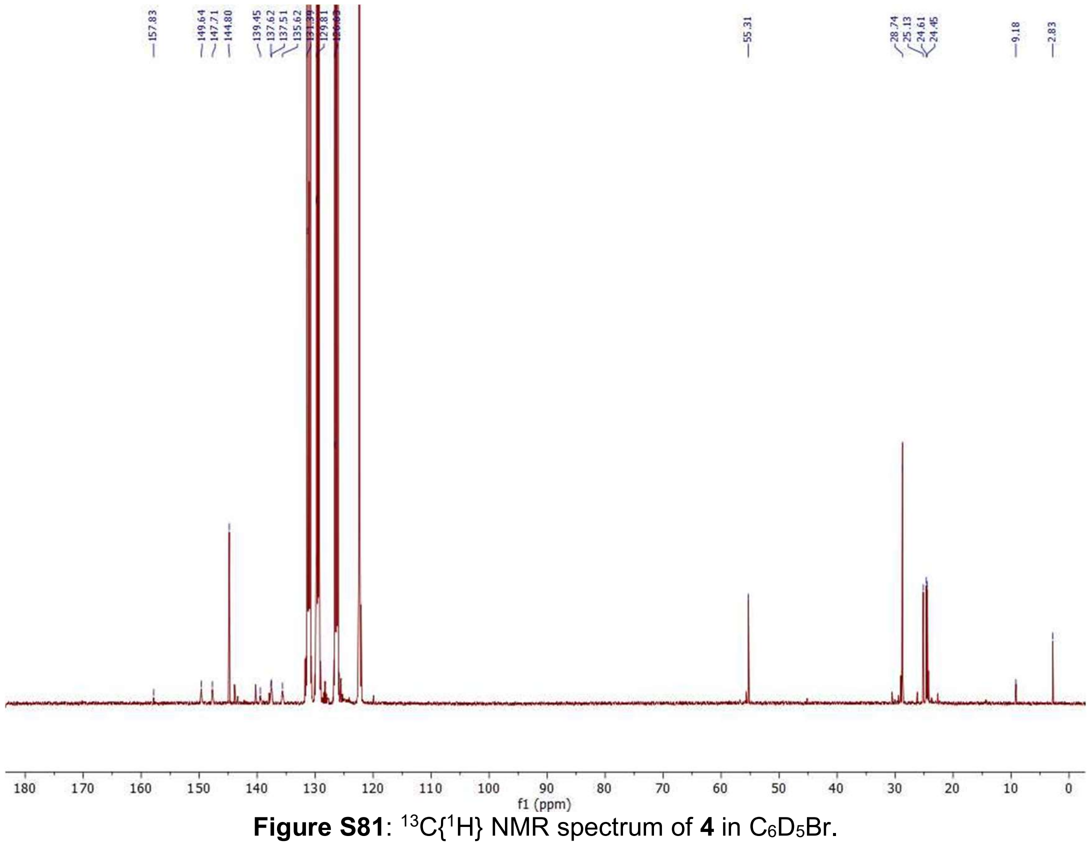

Figure S81: $^{13}C\{^{1}H\}$ NMR spectrum of **4** in C_6D_5Br.

S16. References

1. E. Ihara, V. G. Young and R. F. Jordan, *J. Am. Chem. Soc.*, 1998, **120**, 8277-8278.
2. A. J. Arduengo, R. Krafczyk, R. Schmutzler, H. A. Craig, J. R. Goerlich, W. J. Marshall and M. Unverzagt, *Tetrahedron*, 1999, **55**, 14523-14534.
3. M. Iglesias, D. Beetstra, J. Knight, L.-L. Ooi, A. Stasch, S. Coles, L. Male, M. Hursthouse, K. Cavell, A. Dervisi and I. Fallis, *Organometallics*, 2008, **27**, 3279-3289.
4. R. J. Procter, M. Uzelac, J. Cid, P. J. Rushworth and M. J. Ingleson, *ACS. Catal.*, 2019, **9**, 5760-5771.
5. T. R. Jensen, C. P. Schaller, M. A. Hillmyer and W. B. Tolman, *J. Organomet. Chem.*, 2005, **690**, 5881-5891.
6. D. Specklin, F. Hild, C. Fliedel, C. Gourlaouen, L. F. Veiros and S. Dagorne, *Chem. Eur. J.*, 2017, **23**, 15908-15912.
7. H. Huo, C. Fu, K. Harms, E. Meggers., J. Am. Chem. Soc., 2014, **136**, 8, 2990-2993.
8. K. Nemoto, S. Tanaka, M. Konno, S. Onozawa, M. Chiba, Y. Tanaka, Y. Sasaki, R. Okubo, T. Hattori, Tetrehedron, 2016, **72**, 5, 734-745.
9. L. Salvi, N.R. Davis, S.Z. Ali, S.L. Buchwald, Org. Lett., **14**, 1, 170-173.
10. T-H. Ding, J-P. Qu, Y-B. Kang, Org. Lett., 2020, **22**, 6, 3084-3088.
11. E. Rochette, V. Desroisiers, Y. Yashar, F-G. Fontaine., J. Am. Chem. Soc., 2019, **141**, 31, 12305-12311.
12. Y-L. Liu, G. Kehr, C.G. Daniliuc, G. Erker, Chem. Eur. J., 2017, **23**, 12141-12144.
13. S. Tanaka, S. Yuki, Y. Tanaka, T. Hattori, Org. Lett., 2018, **20**, 7, 1828-1831.
14. V. Bagutski, A. Del Grosso, J. A. Carrillo, I. A. Cade, M. D. Helm, J. R. Lawson, P. J. Singleton, S. A. Solomon, T. Marcelli, M. J. Ingleson, J. Am. Chem. Soc., 2013, **135**, 1, 474-487.
15. H. Ochiai, Y. Uetake, T. Niwa, T. Hosoya, Angew. Chemie. Int. Ed., 2017, **40**, 3, 2333-2342.
16. S. Wurtemberger-Pietsch, H. Schneider, T. B. Marder, U. Radius, Chem. Eur. J., 2016, **22**, 13032-13036.
17. D. C. H. Do, S. Muthaiah, R. Ganguly, D. Vidovic, Organometallics, 2014, **33**, 16, 4165-4168.
18. G. C. Welch, L. Cabrera, P. A. Chase, E. Hollink, J. D. Masuda, P. Wei and D. W. Stephan, *Dalton Trans.*, 2007, 3407-3414.
19. U. Mayer, V. Gutmann and W. Gerger, *Monatsh. Chem.*, 1975, **106**, 1235-1257.
20. M. A. Beckett, G. C. Strickland, J. R. Holland and K. Sukumar Varma, *Polymer*, 1996, **37**, 4629-4631.
21. O. V. Dolomanov, L. J. Bourhis, R. J. Gildea, J. A. K. Howard and H. Puschmann, *J. Appl. Crystallogr.*, 2009, **42**, 339-341.
22. G. Sheldrick, *Acta Crystallog A*, 2015, **71**, 3-8.
23. G. Sheldrick, *Acta Crystallogr. A*, 2008, **64**, 112-122.
24. Gaussian 09, Revision C1, Frisch, M. J., Trucks, G. W., Schleg:el, H. B., Scuseria, G. E., Robb, M. A., Cheeseman, J. R., Scalmani, G., Barone, V., Mennucci, B., Petersson,G. A., Nakatsuji, H., Caricato, M., Li, X., Hratchian, H. P., Izmaylov, A. F., Bloino, J.,Zheng, G., Sonnenberg, J. L., Hada, M., Ehara, M., Toyota, K., Fukuda, R., Hasegawa,J., Ishida, M., Nakajima, T., Honda, Y., Kitao, O., Nakai, H., Vreven, T., Montgomery,Jr., J. A., Peralta, J. E., Ogliaro, F., Bearpark, M., Heyd, J. J.,

Brothers, E., Kudin, K.N., Staroverov, V. N., Kobayashi, R., Normand, J., Raghavachari, K., Rendell, A.,Burant, J. C., Iyengar, S. S., Tomasi, J., Cossi, M., Rega, N., Millam, J. M., Klene, M.,Knox, J. E., Cross, J. B., Bakken, V., Adamo, C., Jaramillo, J., Gomperts, R.,Stratmann, R. E., Yazyev, O., Austin, A. J., Cammi, R., Pomelli, C., Ochterski, J. W.,Martin, R. L., Morokuma, K., Zakrzewski, V. G., Voth, G. A., Salvador, P., Dannenberg,J. J., Dapprich, S., Daniels, A. D., Farkas, Ö., Foresman, J. B., Ortiz, J. V., Cioslowski,J., Fox, D. J. Gaussian, Inc., Wallingford CT, 2009.

25. (a) Becke, A. D. *J. Chem. Phys.* 1993, *98*, 5648-5652, (b) Burke, K, Perdew, J. P. , Yang, W. Electronic Density Functional Theory: Recent Progress and New Directions, (Ed: J. F. Dobson, G. Vignale, M. P. Das), Springer, Heildelberg, **1998**.
26. Zhao, Y., Truhlar, D. G. *Theor. Chem. Acc.* **2008**, *120*, 215-241.
27. Lee, C., Yang, W., Parr, R.G. *Phys. Rev. B* 1988, *37*, 785-789.
28. Grimme, S., Ehrlich, S., Goerigk, L. *J. Comput. Chem.* **2011**, *32*, 1456– 1465
29. Mennucci B., Cancès E., Tomasi J., *J. Phys. Chem. B* **1997**, *101*, 10506-10517.
